# Membrane-based TBADT recovery as a strategy to increase the sustainability of continuous-flow photocatalytic HAT transformations

**DOI:** 10.1038/s41467-022-33821-9

**Published:** 2022-10-18

**Authors:** Zhenghui Wen, Diego Pintossi, Manuel Nuño, Timothy Noël

**Affiliations:** 1 Flow Chemistry Group, Van ‘t Hoff Institute for Molecular Sciences, Faculty of Science, University of Amsterdam, Science Park 904, 1098 XH Amsterdam, The Netherlands; 2 Vapourtec Ltd., Park Farm Business Centre, Fornham St Genevieve, Bury St Edmunds, Suffolk, IP28 6TS UK

**Keywords:** Chemical engineering, Synthetic chemistry methodology

## Abstract

Photocatalytic hydrogen atom transfer (HAT) processes have been the object of numerous studies showcasing the potential of the homogeneous photocatalyst tetrabutylammonium decatungstate (TBADT) for the functionalization of C(sp^3^)–H bonds. However, to translate these studies into large-scale industrial processes, careful considerations of catalyst loading, cost, and removal are required. This work presents organic solvent nanofiltration (OSN) as an answer to reduce TBADT consumption, increase its turnover number and lower its concentration in the product solution, thus enabling large-scale photocatalytic HAT-based transformations. The operating parameters for a suitable membrane for TBADT recovery in acetonitrile were optimized. Continuous photocatalytic C(sp^3^)-H alkylation and amination reactions were carried out with in-line TBADT recovery via two OSN steps. Promisingly, the observed product yields for the reactions with in-line catalyst recycling are comparable to those of reactions performed with pristine TBADT, therefore highlighting that not only catalyst recovery (>99%, TON > 8400) is a possibility, but also that it does not happen at the expense of reaction performance.

## Abstract (web summary)

Decatungstate-enabled Hydrogen Atom Transfer (HAT) photocatalysis of hydrocarbon scaffolds enabled rapid access to new C(sp3)-enriched chemical space. Here, the authors present in-line TBADT recovery with organic solvent nanofiltration (OSN) as an answer to the concerns surrounding the scale-up of decatungstate HAT reactions, i.e., catalyst loading and cost.

## Introduction

**Fig. 1:**
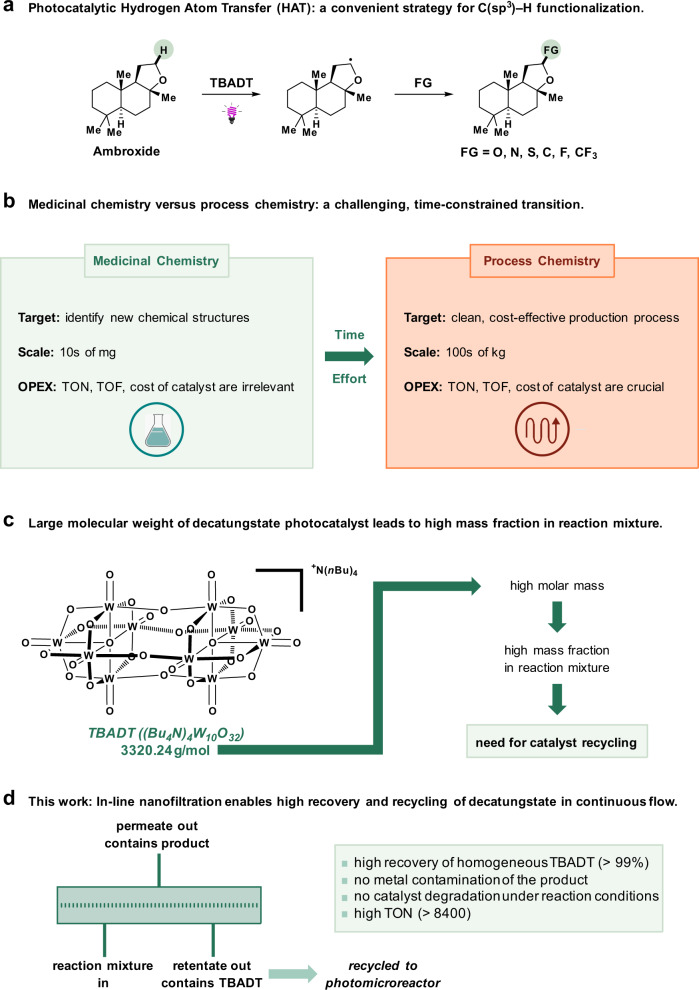
Overview detailing the importance of in-line TBADT recovery. **a** Photocatalytic Hydrogen Atom Transfer enables both early- and late-stage functionalization of hydroalkanes and biologically active compounds. **b** Small-scale versus large-scale synthetic organic chemistry requires different approaches: catalyst lifetime and cost are only relevant on a process chemistry level. **c** TBADT is a high molecular weight molecule resulting in a large mass fraction and thus high associated cost when discarded. This warrants the need for catalyst recycling. **d** We disclose a general and efficient approach for decatungstate recycling using in-line nanofiltration.

In recent years, light-induced hydrogen atom transfer (HAT) presented itself as a versatile strategy for the late-stage functionalization of C(sp^3^)–H bonds without involving transition-metal catalysis or strong oxidants^1,2^. In HAT, the photoexcited catalyst abstracts a hydrogen atom resulting in the formation of reactive radical species, which are exploited for highly selective functionalization. To achieve this selectivity, careful tuning of the steric and electric properties of the HAT photocatalyst and substrate has to take place. Among the limited selection of photocatalysts promoting HAT, arguably the most versatile HAT photocatalyst is the decatungstate anion, which found application in a wide range of transformations, including alkylation^3–8^, arylation^9^, acylation^10^, amination^11–14^, fluorination^15–17^, trifluoromethylation^18^, sulfinylation^19^ and oxygenation (Fig. 1a)^20,21^.

Despite the potential of tetrabutylammonium decatungstate (TBADT) for synthetic applications, the adoption of photocatalytic HAT in industrial processes is still limited. To understand this, one has to consider that while catalyst cost and removal or recycling are minor concerns in demonstrating a new synthetic method, these aspects become major focal points in the transition from small-scale (e.g., required in medicinal chemistry and academia) to large-scale production (Fig. 1b, c)^22,23^. Therefore, among the main hurdles hindering its adoption, the primary concerns are the questions surrounding the ability to scale-up photocatalytic HAT reactions paired with the need to recycle the catalyst^24^ and the requirement to purify the product^25^. The former concern about scalability has been addressed in recent years when continuous-flow chemistry systems proved to be the answer to the scale-up needs of photochemical reactions, as was highlighted in several examples of large-scale photochemical reactions carried out in continuous-flow systems^11,26–35^. For the removal of the catalyst, organic solvent nanofiltration (OSN) could be a suitable strategy for catalyst recovery in continuous-flow systems. OSN is a membrane-based process where selective separation of the species present in the solution is obtained based on their size^36–39^. Molecules smaller than the so-called molecular weight cut-off (MWCO) of the membrane will pass through the membrane, while species larger than the MWCO are selectively retained. Typical MWCO values for commercial OSN membranes range from 150 to 1000 Da, thus making them ideal for the selective separation between homogeneous catalysts having a large molecular weight, such as TBADT (3320 Da), and smaller reaction products^38,40^. Additionally, OSN has the distinct advantage of low energy requirements since phase transitions such as evaporation are avoided in this separation technique. The absence of thermal transitions also removes the issue of thermal stress for the recovered catalyst, which is present in other recovery techniques. The work of Livingston and co-workers highlighted the performance of OSN with commercial membranes for the recovery of a homogeneous catalyst in Heck couplings performed in continuous flow^41^. Additionally, recent studies from Guerra et al. and O’Neal and Jensen demonstrated the suitability of coupling OSN for catalyst recovery with flow microreactors. Similarly, O’Neal and Jensen implemented a small-scale custom OSN unit in an automated setup performing ring-closing metathesis with a recycled second-generation Hoveyda-Grubbs catalyst^42^. Some other reports focused on a combination of photocatalysts with classical polymers to synthesize recyclable photocatalysts^43^. Kappe et al. developed a macromolecular Ru(bpy)_3_^2+^-based dendrimeric catalyst as a photocatalyst, and proved its facile recycling with OSN technology in continuous-flow condition^40^. Although recent works showcased the potential of OSN in flow chemistry, they also highlighted the challenges of catalyst synthesis, deactivation, and membrane fouling.

**Fig. 2:**
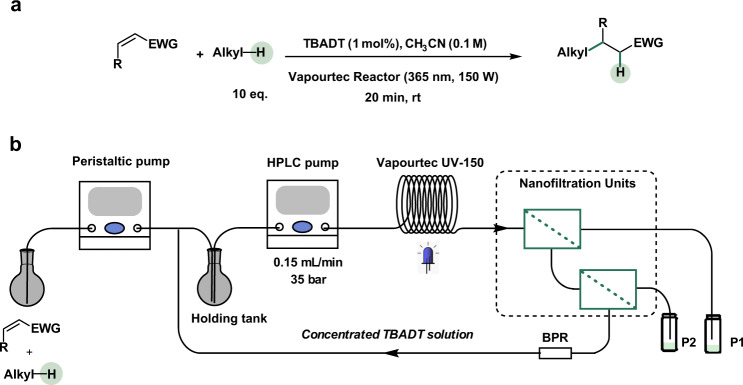
C(sp^3^)–H alkylation with in-line TBADT recovery via nanofiltration. **a** TBADT-catalyzed C(sp^3^)–H alkylation investigated in the present work. **b** flow chart of the multi-stage nanofiltration-based continuous-flow system employed for the recovery of TBADT. EWG electron withdrawing group, BPR back pressure regulator.

Herein, we describe a continuous-flow system coupling a micro-flow photoreactor with OSN-based in-line TBADT recovery (Fig. 1d). The performance of photocatalytic HAT in the form of various photocatalytic C(sp^3^)–H alkylation and amination reactions carried out with recycled TBADT (Fig. 2a) were investigated in conjunction with a two-stage OSN unit for the in-line recovery of TBADT (Fig. 2b), proving the concept of scalable TBADT-based HAT transformations with low catalyst loading and high turnover number (TON).

## Results and discussion

### OSN membrane screening

To achieve a successful C(sp^3^)–H functionalization reaction in continuous flow with in-line catalyst recycling via nanofiltration, suitable commercial membranes were initially evaluated. While the large molecular weight and stability of TBADT make it an ideal candidate for membrane-based recovery, its poor solubility in solvents other than acetonitrile and acetone^20^ represents a significant challenge due to the limited compatibility of commercial OSN membranes with such solvents. Five commercial membranes were evaluated for potential use for TBADT recovery (see Section 2.5 of Supplementary Information for membrane specifications): three membranes from the Evonik PuraMem product line (Selective, Flux, and Performance), and two membranes from SolSep BV (NF080105 and NF030306). Commercial membranes were screened using a Zaiput 10 cross-flow cell and by measuring the flux and selectivity during filtration of 1 mM TBADT solutions in acetonitrile (see Supplementary Information, section 2.1, Supplementary Figs 1-2). Of the membrane pool taken into consideration, only NF080105 and NF030306 from SolSep BV could withstand filtration experiments in acetonitrile. PuraMem Flux and Performance failed to reach the operating pressure, thus indicating damage from the exposure to the solvent, while PuraMem Selective achieved the desired pressure, but exhibited poor TBADT rejection (i.e., high catalyst concentration in the permeate solution, see Supplementary Fig. 7 in the Supplementary Information). Therefore, NF080105 and NF030306 were chosen for further testing involving longer experiment times and higher pressures, which highlighted the unsuitability of NF080105 due to a high failure rate during prolonged filtration experiments paired with issues of catalyst accumulation in the membrane, which negatively affected the membrane flux and mass balance during the filtration (Supplementary Information, Section 2.5, Supplementary Fig. 10). Therefore, the outcome of the membrane screening was that NF030306 is a suitable membrane for OSN of acetonitrile solutions containing TBADT, owing to its resistance to acetonitrile and MWCO in the range of 500–800 Da (Supplementary Table 1 in the Supplementary Information). An important consideration is that the high chemical resistance of this membrane comes at the expense of a relatively low membrane flux, resulting in a limited permeate-to-retentate ratio. However, this issue can be obviated with an appropriate design of the filtration stages, as shown in the discussion below.

### Nanofiltration process conditions screening

Once a membrane candidate was secured, the following step was optimizing filtration performance as a function of operating pressure and input flow rate. As the Zaiput cell was limited to 20 bar maximum pressure, a new cross-flow cell was designed in-house to achieve pressures up to 40 bar (operating limit for the NF030306, according to its specifications). The cell design is detailed in Supplementary Information, Section 2.6. Supplementary Fig. 17 in Supplementary Information presents the results of the screening of operating pressures of TBADT recovery with nanofiltration. As expected, higher pressures resulted in an increased flux without significantly affecting TBADT recovery. However, at 40 bar, undesired TBADT precipitation occurred (Supplementary Fig. 18 in the Supplementary Information), leading to decreased catalyst concentration in the retentate solution and, thus, reduced catalyst recovery. For this reason, an operating pressure of 35 bar was selected for further experiments. Supplementary Fig. 19 in Supplementary Information details the screening of flow rates. Longer residence times in the cell resulted in increased transport across the membrane. However, a decreased flux was observed for the lowest flow rate, which can be explained by reduced mixing in the cell and concentration polarization across the membrane. Therefore, the flow rate for catalyst recovery was maintained at 0.15 mL/min. The result of the membrane screening and operating parameters optimization proved the potential of OSN for TBADT recovery, with an achieved recovery exceeding 98%.

The potential negative impact on catalyst activity of the recycling process was taken into consideration and tested by performing alkylation reactions with TBADT that were obtained either via OSN or via non-solvent extraction and filtration. In both cases, product yields comparable to those achieved with pristine TBADT were measured (Supplementary Fig. 20 and Supplementary Table 2 in Supplementary Information). This result highlights that catalyst recycling has no observable detrimental effects on the stability and catalytic activity of TBADT. We believe this is due to the molecular structure of TBADT which is not prone to radical attack. This is in contrast to organic dyes and Ru- or Ir-based photocatalysts where the ligands, often polypyridyl moieties, are excellent acceptors for radicals and are the main cause of catalyst degradation^44^.

### OSN performance investigation during the photocatalytic C(sp^3^)–H alkylation reaction

When moving from TBADT solution to the reaction mixture, undesired interactions of substances in the reaction mixture, such as starting materials, products, or byproducts, with the membrane may negatively affect filtration performance. Therefore, to evaluate potentially undesired effects of chemicals present in the reaction mixture, filtration experiments were performed with mixtures containing an increasing number of substances: TBADT, TBADT and cyclohexane, and the complete reaction mixture. For all experiments, comparable fluxes were observed (Supplementary Fig. 21 in Supplementary Information). Therefore, a negative influence of reaction components, particularly cyclohexane, was ruled out.

The model reaction chosen to investigate in-line catalyst recovery was the same alkylation reaction optimized by Wen et al.^4^, albeit with a different substrate (dimethyl maleate). The starting material was changed to avoid any UV-vis absorbance of the substrate and product in the same wavelength range of the TBADT peak, thus enabling the determination of the TBADT concentration from the measurement of UV-vis spectra of the retentate and permeate streams obtained during filtration experiments. The validity of the previously identified optimal reaction conditions was confirmed by varying the catalyst loading, the number of cyclohexane equivalents, and the residence time of the reaction involving the new substrate (Supplementary Tables 3–5 in Supplementary Information).

**Fig. 3:**
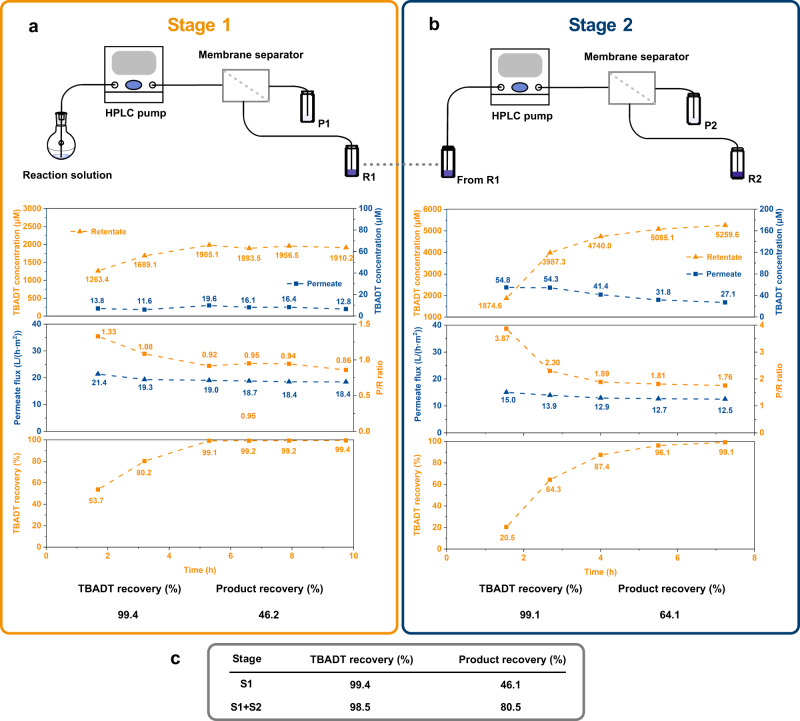
OSN performance investigation with a photocatalytic hydroalkylation reaction. **a** Results of the nanofiltration experiment of the reacted mixture (Stage 1): concentration of TBADT in the retentate (R) and permeate (P); permeate flux; catalyst and product recovery. **b** Results of the nanofiltration experiment of the retentate solution from Stage 1 (Stage 2): concentration of TBADT in the retentate and permeate; permeate flux; catalyst and product recovery. **c** Summary of the overall recovery process for the two NF stages.

To design the in-line continuous-flow catalyst recycling, detailed knowledge of the OSN performance with the solution collected at the outlet of the flow reactor was key. Therefore, a batch study was performed by collecting and filtering the solution resulting from the model alkylation reaction (Fig. 2a). Encouraging results were obtained when performing single-stage filtration, with catalyst retention in the retentate side approaching total rejection (99.4% recovery) after the initial transient period when membrane compaction and equilibration with the surrounding environment resulted in slowly improving filtration performance that stabilized over a few hours (Fig. 3a). Despite the high catalyst recovery, limited flux through the membrane resulted in a relatively low permeate volume, which in turn affected the overall product recovery for a single stage (48.1%), since a large fraction of the product remained in the retentate volume. It should be noted that the product transport through the membrane is not selective, resulting in equal concentrations in permeate and retentate. For this reason, the product recovery is only dependent on the permeate-to-retentate volumetric ratio. Therefore, to increase the overall permeate volume and in turn product recovery, a second filtration stage was added to the process, where the retentate solution still containing a relatively large amount of product was filtered through a second OSN unit (Fig. 3b). Given the smaller input volume, the second filtration had a lower flow rate, which resulted in equilibrium flux values lower than for the first stage, in line with the results of the flow rate screening for filtration of TBADT solutions. For the second stage, catalyst retention exceeded 99%, resulting in a combined catalyst recovery of 98.4% over the two stages paired with a satisfactory product recovery exceeding 80%. The results of these experiments highlighted the trade-off between catalyst recovery and product recovery for a membrane with limited flux and high chemical resistance. Nevertheless, the two-stage filtration design enabled overcoming the trade-off and delivered high catalyst recovery coupled with suitable product recovery.

### Photocatalytic C(sp^3^)–H alkylation reaction with in-line TBADT recovery

**Fig. 4:**
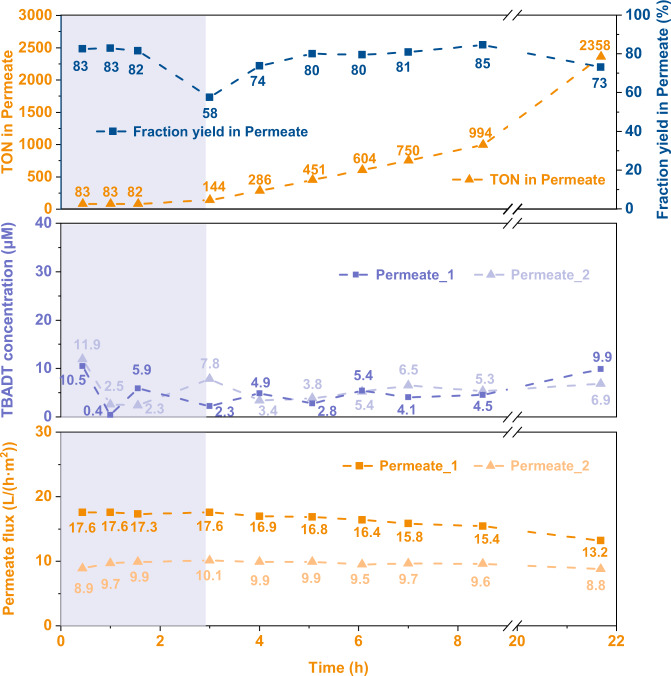
Results of the in-line catalyst recovery experiment. Data about turnover number and measured reaction yield, residual TBADT concentration in the permeate solution, and permeate flux is included. The equilibration phase of the experiment is reported with a shaded background.

Given the results of the batch study, a continuous-flow alkylation reaction with in-line catalyst recovery was designed (Fig. 2b), with optimized conditions for both the reaction and filtration processes enabled by the choice of the reactor volume to achieve the desired residence time in the reactor and the optimal flow rate in the first separation unit. After equilibrating the system during the start-up phase, in-line catalyst recycling was initiated by feeding back the retentate from the second OSN stage to the mixture of dimethyl maleate and cyclohexane in acetonitrile. The first run of 19 h was performed (Fig. 4), achieving total TONs of 2659. The product yield measured throughout the experiment was close to that of the optimized reaction performed with pristine TBADT and only decreased toward the end of the experiments. This is explained by a decrease in the catalyst concentration towards the end of the experiment, which was the consequence of the peristaltic pump used to deliver the substrate solution slowly deviating from its calibrated values and increasing the effective flow rate delivered to the holding tank. This resulted in extra dilution of the catalyst in the dimethyl maleate solution, leading to a decreased fraction yield.

**Fig. 5:**
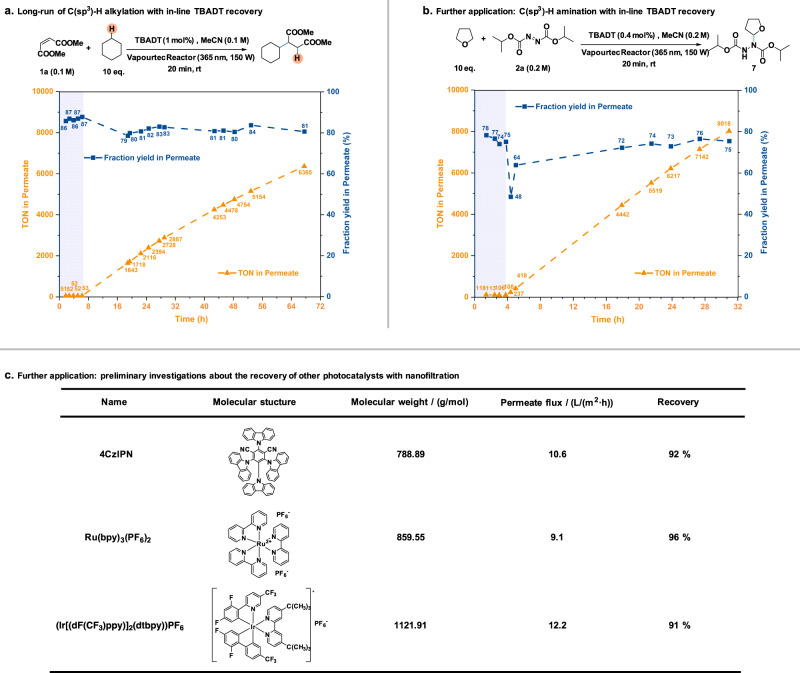
Practicality investigations of the nanofiltration process. **a** Fraction yield and TON in permeate of a 60-h run of C(sp^3^)–H alkylation executed with in-line TBADT recovery. The equilibration phase of the experiment is reported with a shaded background. **b** Fraction yield and TON in permeate of a 27-h run of C(sp^3^)–H amination executed with in-line TBADT recovery. The equilibration phase of the experiment is reported with a shaded background. **c** Preliminary investigations of the recovery of other commercially available photocatalysts with nanofiltration. For more experimental details see Supplementary Information.

Given the promising results of the 19 h run, a 3-day catalyst recycling experiment was performed (Fig. 5a). This replica of the previous experiment, albeit on a longer time scale, confirmed that the dip in fraction yield was due to an issue with the pump, feeding the substrate solution, and a short decline during the start of the recirculation mode. Throughout the 3-day period, the fraction yield of the model alkylation oscillated in a narrow range of 79–84%, and a total TON of 6738 (including additional 373 TONs remaining in the system) was achieved by the end of the 3-day catalyst recycling experiment. Notably, for this longer experimental run, some precipitation of TBADT on the membrane surface was detected during disassembly. The presence of fouling on the membrane surface explains the observed membrane flux decrease. Nevertheless, the deposited TBADT on the membrane surface could be easily washed away with pure acetonitrile. Therefore, to address the issue of catalyst deposition on the membrane surface, periodical (back)washing of the membrane with pure solvent could be implemented (e.g., 10 min wash with acetonitrile for every 8 h of operation).

### Scope of the photocatalytic C(sp^3^)–H alkylation in combination with in-line TBADT recovery

**Fig. 6:**
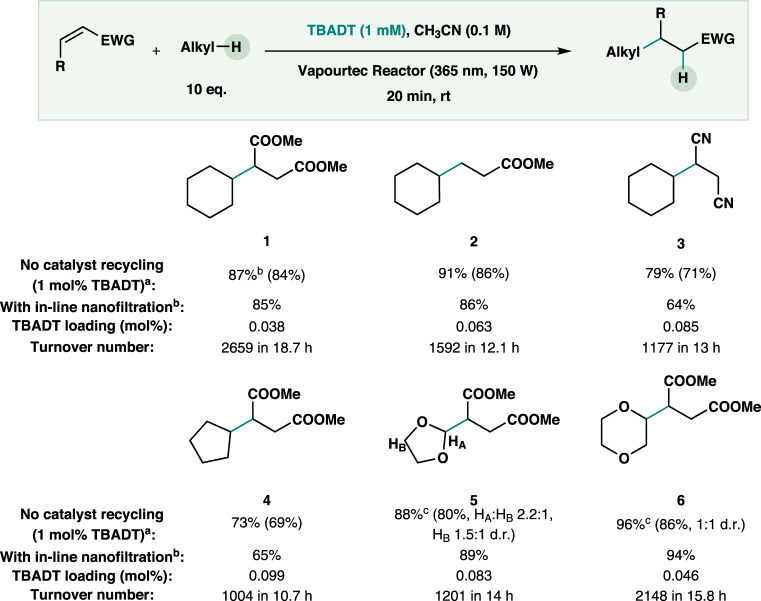
Scope of the photocatalytic C(sp^3^)–H alkylation executed with in-line TBADT recovery. ^a^ Reaction conditions for single run without catalyst recycling: alkene (0.1 M), H-donor (10 equiv), TBADT (1 mol%) in CH_3_CN (5 mL). Irradiation source: *λ* = 365 nm (150 W), residence time: 5 min (see the Supplementary Information for additional details). Isolated yields are given in parentheses. ^b^ The yield was determined by ^1^H-NMR using 1,2,4,5-tetramethylbenzene as external standard. ^c^ 1.2 mol% TBADT.

The in-line TBADT recovery proves the potential of OSN for catalyst recovery, thus helping bridge the gap between small-scale HAT transformation and their scaled-up counterparts. Another advantage of the use of OSN for catalyst recovery is the flexibility to either scale up by numbering up, with the possibility to explore different configurations between stages, or to size up. To further highlight the potential of this technique, a small scope for the model photocatalytic hydroalkylation was performed (Fig. 6), reporting both the product yield for the reaction carried out with pristine TBADT and the product yield for the reaction performed with the in-line catalyst recovery configuration depicted in Fig. 2b. Different electron-deficient alkenes, including esters and fumaronitrile were all good Michael acceptors, delivering good yields of the targeted products (**1** to **3**). Cyclopentane underwent the C–C bond-forming reaction promptly, transforming the unactivated alkyl substrate into a targeted hydroalkylated compound (**4**). Esters (1,3-dioxolane and 1,4-dioxane) were used as H-donors for the radical addition of dimethyl maleate, which afforded the targeted products (**5**, **6**) in excellent yields. Promisingly, for all entries in the scope, the product yields are similar to those obtained without catalyst recycling, proving the generality of our in-line catalyst recycling strategy.

### Generality of the nanofiltration process for the recovery of other photocatalysts

To further highlight the generality of our in-line catalyst recycling strategy, a second TBADT-based transformation was investigated and three commercial photocatalysts were screened for compatibility with the in-line filtration process. The new transformation investigated is a decatungstate-mediated C(sp^3^)–H amination. Using the same in-line catalyst recycling strategy, within 27 h, a total TON of 8423 was obtained (Fig. 5b), including TON of 8018 from the product in permeate solution and additional 405 TONs remaining in the system. Additionally, three commercial photocatalysts (4CzIPN, Ru(bpy)_3_(PF_6_)_2_, (Ir[(dF(CF_3_)ppy)]_2_(dtbpy))PF_6_)) (Fig. 5c) were screened with filtration tests, and all achieved catalyst recovery exceeding 90% (Section 6 in Supplementary Information).

To conclude, this work presents in-line TBADT recovery as an answer to the concerns surrounding the scale-up of TBADT-catalyzed photocatalytic HAT reactions, where catalyst loading, cost, and removal are major concerns. A suitable OSN membrane was identified, and the operating conditions and experimental design were optimized to achieve suitable catalyst and product recovery. Notably, the product yield of reactions performed with in-line catalyst recovery is comparable to those carried out with pristine catalyst, proving that not only TBADT recovery is technically feasible, but it also does not compromise reaction performance. Considering the widespread interest in decatungstate-enabled HAT photocatalysis, we expect this in-line catalyst recovery protocol will be particularly useful for process chemists to reduce the overall operational cost of the scaled-up process.

## Methods

### General procedure for C(sp^3^)-H alkylation with in-line TBADT recovery

A 50 mL volumetric flask was charged with olefin (0.1 M, 5 mmol, 1 equiv.), H-donors (1 M, 50 mmol, 10 equiv.) and TBADT (1 mM, 50 μmol, 1 mol%). Next, CH_3_CN was added to acquire a total volume of 50 mL. The prepared solution was transferred to the holding tank and delivered to a Vapourtec UV-150 Reactor (PFA tubing, 3.06 mL, 750 μm inner diameter) via HPLC pump. The outlet solution of photoreactor is directly injected into the two-stage flow cells. Samples from permeate and retentate solutions were collected and analyzed until reaching the equilibrium of the membrane. Another solution with olefin and H-donors in acetonitrile was prepared, which was delivered to the holding tank by the peristaltic pump. Meanwhile, the retentate feed was put into the holding tank, and mixing with the fresh starting material solution. The samples were collected from two permeate feeds and analyzed with ^1^H-NMR. After a certain experimental time, pure acetonitrile was used to push out the solution left inside the system. A total TON was calculated by combining solutions in permeate feeds and remaining in the system. A detailed description of the experimental setup and protocols is available in Section 3.3 of the Supplementary Information.

## Supplementary information

Supplementary Information

## Data Availability

All relevant data generated in this study are provided in the article and the supplementary Information file. FAIR data (primary NMR FID files) for compounds **1**–**7** can be found at 10.6084/m9.figshare.20407098. Source data are provided with this paper.

## Source data

Source Data
