# Supplementary material for: Membrane-based TBADT recovery as a strategy to increase the sustainability of continuous-flow photocatalytic HAT transformations
Source: Nat Commun. 2022 Oct 18;13:6147. doi: 10.1038/s41467-022-33821-9 (PMC9579200; doi:10.1038/s41467-022-33821-9)
Supplement: Supplementary file 1 — Supplementary Information [file 41467_2022_33821_MOESM1_ESM.pdf]

Supplementary Information

Membrane-based TBADT recovery as a strategy to increase the sustainability of continuous-flow photocatalytic HAT transformations

Zhenghui Wen[1,+], Diego Pintossi[1,+], Manuel Nuño,[2] Timothy Noël[1]*

[1.] Flow Chemistry Group, van't Hoff Institute for Molecular Sciences (HIMS), University of Amsterdam (UvA), Science Park 904, 1098 XH, Amsterdam, The Netherlands.

[2] Vapourtec Ltd., Park Farm Business Centre, Fornham St Genevieve, Bury St Edmunds, Suffolk IP28 6TS, United Kingdom.

[+] These authors equally contributed to the manuscript.

*Corresponding author: t.noel@uva.nl

Table of Contents

Supplementary Methods 3

1. General information 3
2. Nanofiltration process 4
   - 2.1 General procedure for membrane screening 4
   - 2.2 Sample preparation for nanofiltration membranes 5
   - 2.3 Inline UV/Vis measurement 6
   - 2.4 Definitions 7
   - 2.5. Membrane screening 9
   - 2.6 High-pressure membrane separator design 14
   - 2.7 Operating pressure screening 17
   - 2.8 Input flow rate screening 18
   - 2.9 TBADT stability test 19
   - 2.10 Potentially undesired effect of membrane-reactant interactions 21
   - 2.11 Estimation of how many NF stages needed to reach 95% product recovery 22
3. General reaction procedure 23
   - 3.1 General procedure 1 (GP1) for single run of C(sp^3)-H alkylation: 23
   - 3.2 General procedure 2 (GP2) for single run of C(sp^3)-H amination: 23
   - 3.3 General procedure 3 (GP3) for inline nanofiltration: 23
4. Reaction Optimization 26
   - 4.1 General experimental procedure for screening alkylation reaction: 26
   - 4.2 TBADT loading screening 27
   - 4.3 Cyclohexane equivalent screening 28
   - 4.4 Residence time screening 29
   - 4.5 Pressure screening 30
   - 4.6 General experimental procedure for screening C(sp^3)-H amination reaction: 31
   - 4.7 C(sp^3)-H Amination reaction conditions screening 32
5. Long-term experiment of C(sp^3)-H alkylation 33
6. Preliminary investigations of other photocatalysts recovery with Nanofiltration 35
   - 6.1 4CzIPN 35
   - 6.2 $Ru(bpy)_3(PF_6)_2$ 37
   - 6.3 $(Ir[(dF(CF_3)ppy)]_2(dtbpy))PF_6$ 38
7. Characterization data 39
8. NMR spectra 44

Supplementary reference 54

Supplementary Methods

1. General information

All reagents and solvents were used as received without further purification, unless stated otherwise. Reagents and solvents were bought from Sigma Aldrich, TCI and Fluorochem. Technical solvents were bought from Biosolve and were used as received. The catalyst TBADT (tetrabutylammonium decatungstate, $(n\text{-}Bu_4N)_4W_{10}O_{32}$) was prepared according to a published procedure.[1] All the membranes mentioned in this paper are commercially available. NF080105 and NF030306 were bought from SolSep BV. PuraMem® membranes were purchased from modular process solutions AG, which is a distributor of EVONIK Industries in Europe. For membrane screening, a Zaiput SEP-10 separator was used, which was bought from Zaiput Flow Technologies. For flow experiments, a Vapourtec UV-150 device was used, equipped with 150 W 365 nm LEDs. The capillary tubing was purchased from APT Tubing. All microfluidic fittings were purchased from IDEX Health & Science. Disposable syringes were from BD Discardit II® or NORM-JECT®, purchased from VWR Scientific. HPLC pump was purchased by Shimadzu model LC-20AD. Peristaltic pump was from Masterflex Ismatec™, purchased from Fisher Scientific. Product isolation was performed automatically by a Biotage® Isolera Four, with Biotage® SNAP KP-Sil 10 or 25 g flash chromatography cartridges. TLC analysis was performed using Silica on aluminium foils TLC plates (F254, Supelco Sigma-Aldrich™) with visualization under ultraviolet light (254 nm and 365 nm). ^1H (300 MHz) and ^{13}C (75 MHz) spectra were recorded on ambient temperature using a Bruker-Avance 300. ^1H NMR spectra are reported in parts per million (ppm) downfield relative to $CDCl_3$ (7.26 ppm) and all ^{13}C NMR spectra are reported in ppm relative to $CDCl_3$ (77.16 ppm), unless stated otherwise. In the NMR spectra the following abbreviations were used to describe the multiplicity: s = singlet, d = doublet, t = triplet, q = quartet, p = pentet, h = hextet, hept = heptet, m = multiplet, dd = double doublet, td = triple doublet. NMR data was processed using the MestReNova 12.0 software package. Known products were characterized by comparing to the corresponding ^1H NMR and ^{13}C NMR from literature. GC-MS data was recorded on a Shimadzu GC2010 Plus gas chromatograph coupled with GCMS-QP2010-SE mass spectrometer using a SH-Rtx-5 Amine column (30 m × 0.25 mm × 0.25 µm). High-resolution mass spectra (HRMS) were collected on an AccuTOF GC v 4g, JMS-T100GCV Mass spectrometer (JEOL, Japan). UV-Vis spectra were

recorded on an inline UV-Vis spectrometers from AvaSpec StarLine family of Avantes. The names of all products were generated using the PerkinElmer ChemDraw 20.1 software package.

2.Nanofiltration process

2.1 General procedure for membrane screening

In this paper, five organic solvent nanofiltration membranes were selected to perform the TBADT recovery. The general procedure for membrane screening was as shown in **Supplementary Figure 1**. After preparation of the membrane sample (**section 2.2**), each nanofiltration membrane was placed in the middle of Zaiput SEP-10 separator (**Supplementary Figure 2**). The TBADT solution or reaction solution was delivered to the membrane separator by HPLC pump. The outflows of the separator, retentate and permeate, were collected in two individual bottles. The collected solution was weighted and then analysed with an inline UV-Vis spectrophotometer. Based on the weights and TBADT concentrations of each solution, the TBADT recovery and performance of membrane was evaluated.

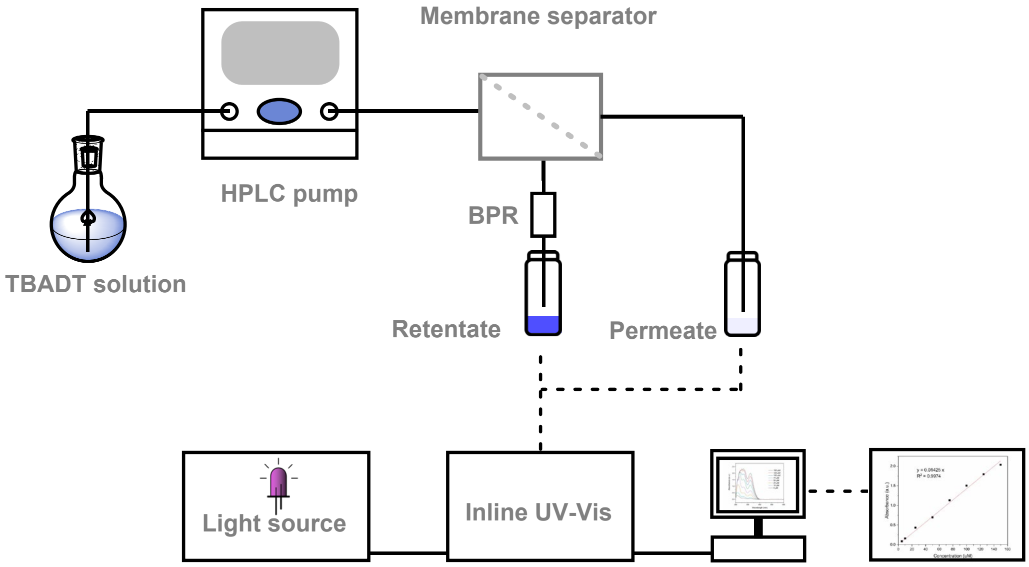

Supplementary Figure 1. General procedure for membrane screening.

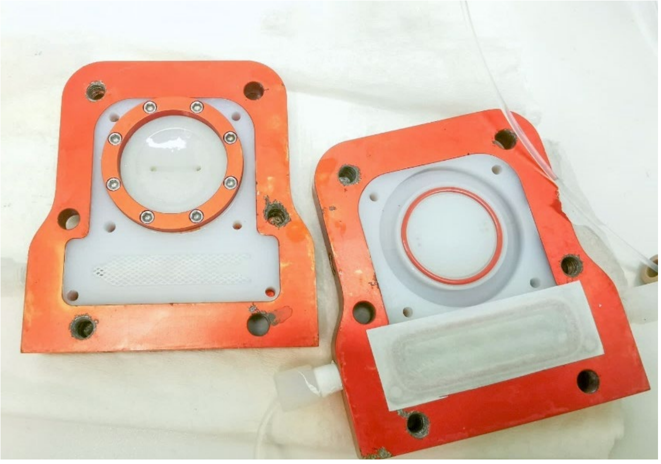

Supplementary Figure 2. Picture of Zaiput SEP-10 used for membrane screening.

2.2 Sample preparation for nanofiltration membranes

The processing of each nanofiltration membrane follows those steps below.

1. Always place the membrane between two protecting papers before you cut it.
2. Mark the area that you want to cut on one of the papers before you cover the membrane with it.
3. Keep the membrane between the paper while cutting
4. Check the membrane piece by holding it against the light. If you see deep scratches take another sample.
5. Pre-swelling often gives more reliable results. This can be done in the cell or just wet the sample with the solvent for 1-2 hours.
6. Keep the o-ring of the separator at the shiny side of the membrane.
7. Pump the CH_3CN with HPLC pump to separator at the needed flow rate until reaching the desired pressure.

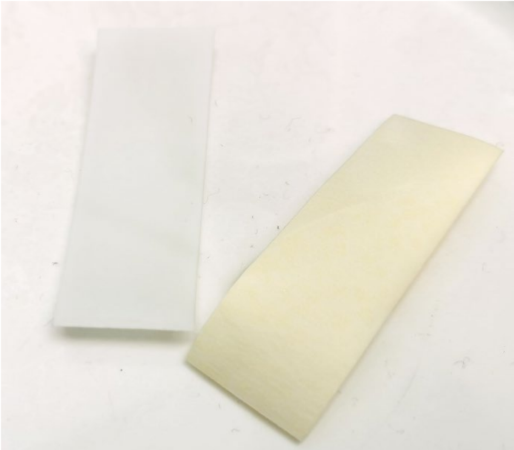

Supplementary Figure 3. Samples of nanofiltration membrane in Acetonitrile. White one is NF080105 from Solsep and yellow one is NF030306 from Solsep.

2.3 Inline UV/Vis measurement

A series of TBADT solutions with different concentrations were prepared with volumetric flasks. The absorbance of the TBADT solution was measured with Avantes® AvaSpec-2048 inline UV/Vis spectrometer (**Supplementary Figure 4**). As shown in **Supplementary Figure 5**, TBADT mainly have two absorbance peaks, 270 nm and 323 nm. Considering that the absorbance at 270 nm is easily saturated and affected by other reactants, we chose the absorbance at 323 nm to derive the calibration curve (**Supplementary Figure 6)**. For measurement purposes, experimental samples were diluted to achieve concentrations that would fall in the linearity region of the TBADT calibration curve.

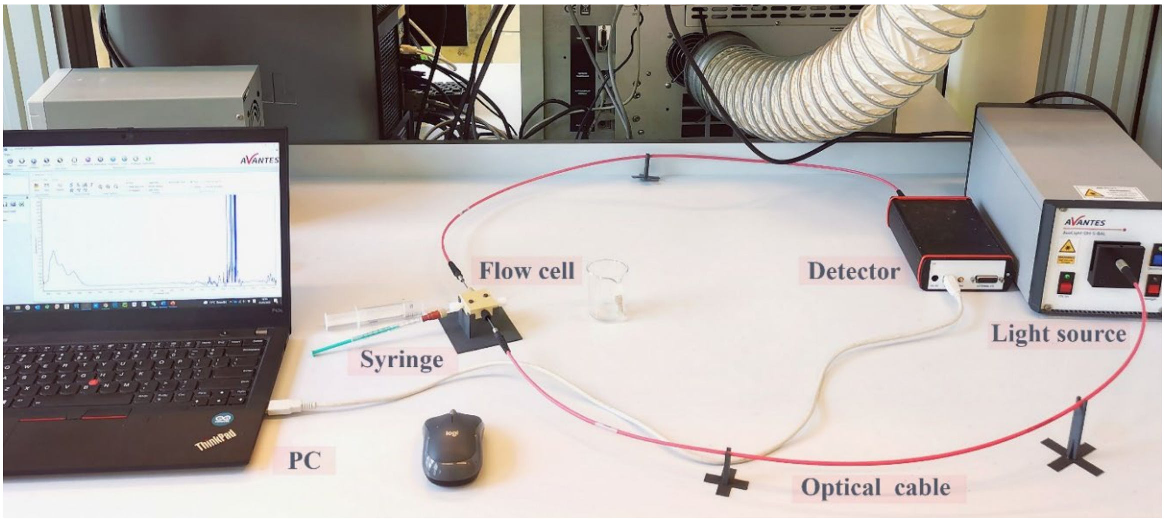

Supplementary Figure 4. Inline UV/Vis measurement setup.

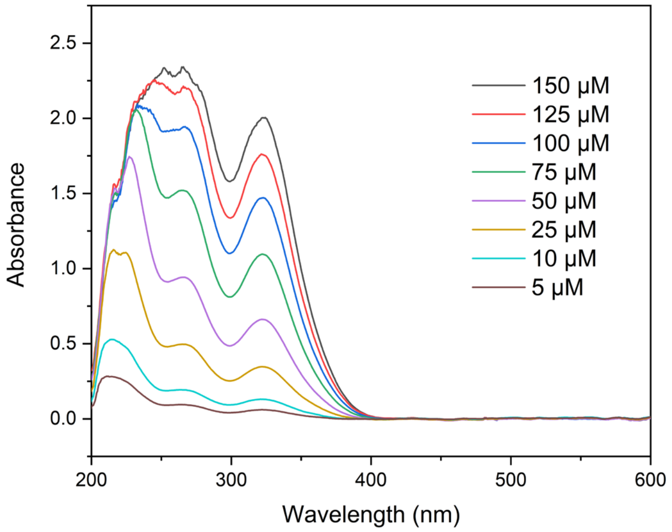

Supplementary Figure 5. Absorbance of TBADT solution with different concentrations.

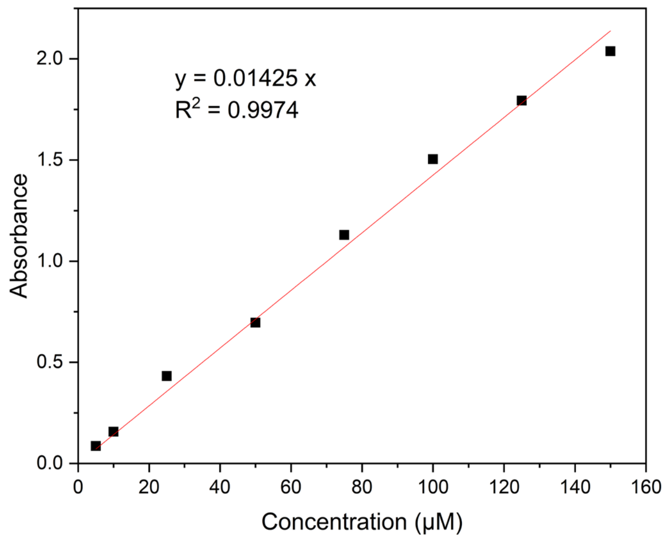

Supplementary Figure 6. Calibration curve of TBADT concentration vs UV/Vis absorbance at 323 nm

2.4 Definitions

Here is a list of the definitions of the items used in this paper. These definitions apply for both the main text and the Supporting Information.

Permeate flux[2]

Permeate flux equals to the volume of liquid that permeates the membrane per unit of time, per membrane area, as shown in **Equation S1**:

$$Permeate\ flux = \frac{V_{permeate}}{Time \times Area}\ (L \cdot m^{-2} \cdot h^{-1}) \tag{S1}$$

where *V* stands for volume (mL).

Permeate/retentate ratio (P/R)

The permeate/retentate ratio (P/R ratio) is defined as the mass of the permeate solution over the mass of retentate solution as shown in **Equation S2**:

$$P/R\ ratio\ = \frac{m_{permeate}}{m_{retentate}} \tag{S2}$$

where *m* stands for mass (g).

TBADT recovery

TBADT recovery equals to the amount of TBADT in retentate divided the input amount of TBADT, as shown in **Equation S3**:

$$TBADT\ recovery = \frac{n_{TBADT}^{R}}{n_{TBADT}^{0}} = \frac{c_{TBADT}^{R} \times V_R}{C_{TBADT}^{0} \times Q_{in} \times t}\ (\%) \tag{S3}$$

where R stands for retentate, Q_{in} is the inlet flow rate of TBADT solution (mL/min); t is the operation time (min).

Product recovery

Product recovery equals to the amount of product in permeate divided the input amount of product, as shown in **Equation S4**:

$$Product\ recovery = \frac{n_{pro}^{P}}{n_{pro}^{0}} = \frac{\sum C_{pro,i}^{P} \cdot V_{P,i}}{C_{pro}^{0} \times Q_{in} \times t}\ (\%) \tag{S4}$$

where P stands for permeate, $C_{pro,i}^{P}$ is product concentration of fraction *i* of permeate solution (M); $V_{P,i}$ is volume of the fraction *i* of permeate solution *(mL);* C_{pro}^{0} is the product concentration of the inlet solution pumped into the separator (M).

Fraction yield in permeate[3]

Fraction yield in permeate equals to the moles of product in permeate solution over the input moles of starting materials, as shown in **Equation S5**:

$$Fraction\ yield\ in\ permeate = \frac{\sum C_{pro,i} \cdot V_{P,i}}{C_{sub} \times Q_{in} \times t}\ (\%) \tag{S5}$$

where P stands for permeate, $C_{pro,i}$ is product concentration of fraction *i* (M); $V_{P,i}$ is volume of the permeate of fraction *i (mL).*

Turnover number in permeate (*TON_P*)[3]

Turnover number in permeate equals to the moles of desired product formed in permeate over the moles of TBADT used as shown in **Equation S6**:

$$TON_P = \frac{n_{pro}^{P}}{n_{TBADT}} = \frac{\sum C_{pro,i} \cdot V_{P,i}}{n_{TBADT}} \tag{S6}$$

Total turnover number (*TON_{total}*)[3]

Total turnover number equals to the moles of desired product formed in the whole process over the moles of TBADT used as shown in **Equation S7**:

$$TON_{total} = \frac{n_{pro}^{P}}{n_{TBADT}} = \frac{\sum C_{pro,i} \cdot V_{P,i} + C_{pro}^{sys} \cdot V_{sys}}{n_{TBADT}} \tag{S7}$$

where C_{pro}^{sys} is the concentration of product in the solution remaining in the system (M); V_{sys} is the volume of the solution remaining in the system (mL).

Turnover frequency (TOF)[4]

Turnover frequency equals to the moles of desired product formed in 1 hour over the moles of TBADT used as shown in **Equation S8**:

$$TOF = \frac{\Delta TON}{\Delta t} \tag{S8}$$

where Δt is the time interval for each sample.

2.5. Membrane screening

Follow the general procedure of membrane screening mentioned above (**Section 2.1**), five types of membranes were screened for TBADT recovery. The detailed information about these membranes is listed in **Supplementary table 1** below.

Supplementary table 1. Summary of the membranes tested for TBADT recovery. [a] The MWCO range indicated by the manufacturer is 500 – 1000 Da, but high rejection is obtained for compounds in the 750 – 800 range, thus indicating that in the current conditions the MWCO is in the range of 500 – 800 Da.

Membrane	Manufacturer	Material	molecular weight cut-off (Da)	Maximum pressure (bar)
PuraMem® Selective	Evonik	Silicone-coated PAN	n.a.	60
PuraMem® Performance	Evonik	Silicone-coated PAN	n.a.	60
PuraMem® Flux	Evonik	Silicone-coated PAN	n.a.	60
NF080105	Solsep	PDMS	500-1000	20
NF030306	Solsep	PDMS	500-1000[a]	40

Given the poor solubility of TBADT in the most common organic solvents, the only suitable solvent was Acetonitrile, which has limited compatibility with most commercial OSN membranes.[5, 6] We started with PuraMem® serial membrane first. Followed with two-hour pre-processing with CH_3CN, we tested those three type membranes with 0.15 mL/min input flow rate. Unfortunately, none of them could reach the desired pressure (14 bar). With the increasing input flow rate to 0.5 mL/min, only the experiment of PuraMem® selective membrane can maintain 5 bar, which was still lower than the desired pressure. As shown in **Supplementary Figure 7**, the permeate flux of PuraMem® selective membrane is 31.2 $L \cdot h^{-1} \cdot m^{-2}$. Additionally, the permeate volume contained a high concentration of TBADT (almost 500

μM), leading to a low TBADT recovery and highlighting the poor selectivity of the PuraMem membrane when used in acetonitrile solutions.

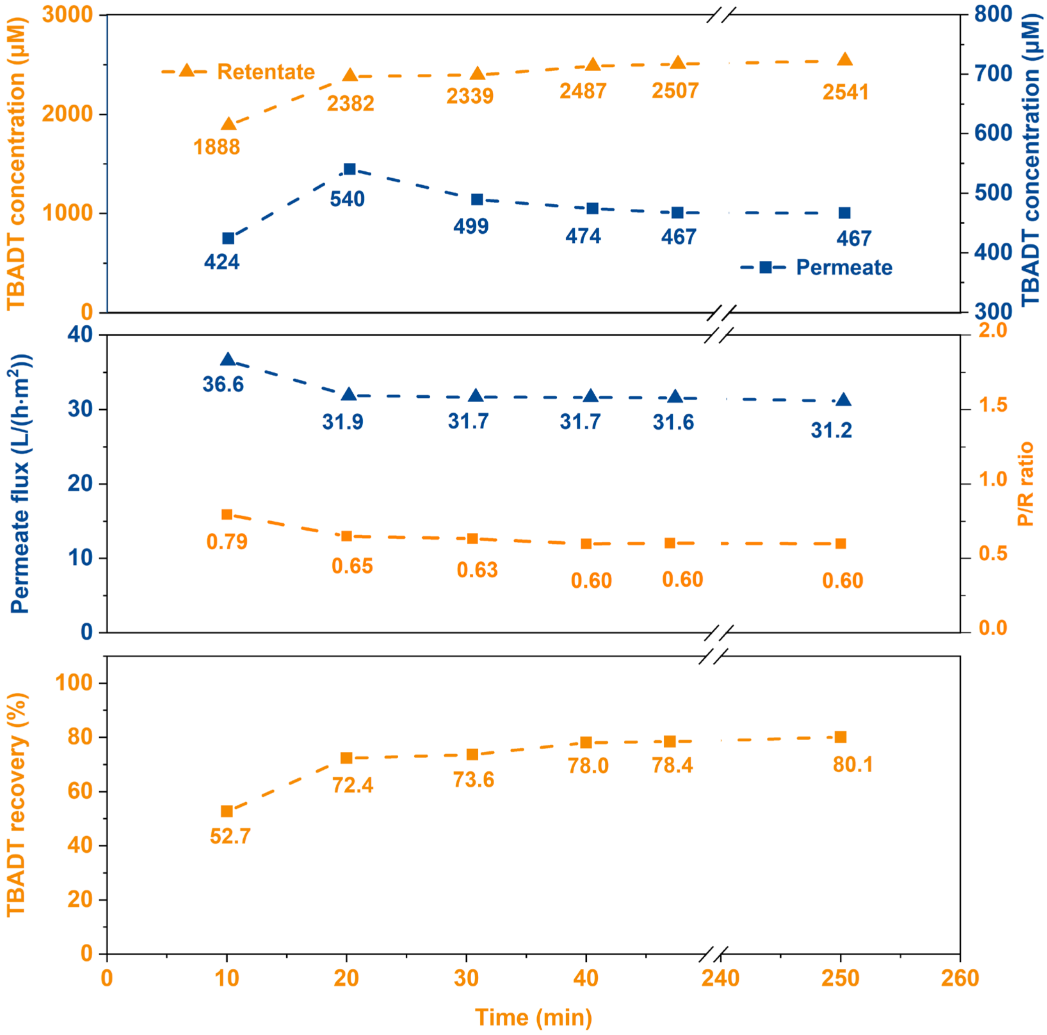

Supplementary Figure 7. Summary of TBADT filtration experiment with PuraMem® Selective membrane. Experiment condition: 0.5 mL/min input flow rate, 5 bar, 2 mM TBADT solution.

Subsequently, membranes from SolSep. B.V., NF080105 and NF030306, were also tested with Zaiput SEP-10 after two-hour pre-processing with CH_3CN. In **Supplementary Figure 8**, the TBADT concentration of permeate solution was relatively low compared to that of the retentate solution. However, the calculated TBADT recovery was below 70%. The low catalyst recovery paired with a mismatch between the input amount of TBADT and the measured output amount, led to the hypothesis of catalyst accumulation occurring in the membrane.

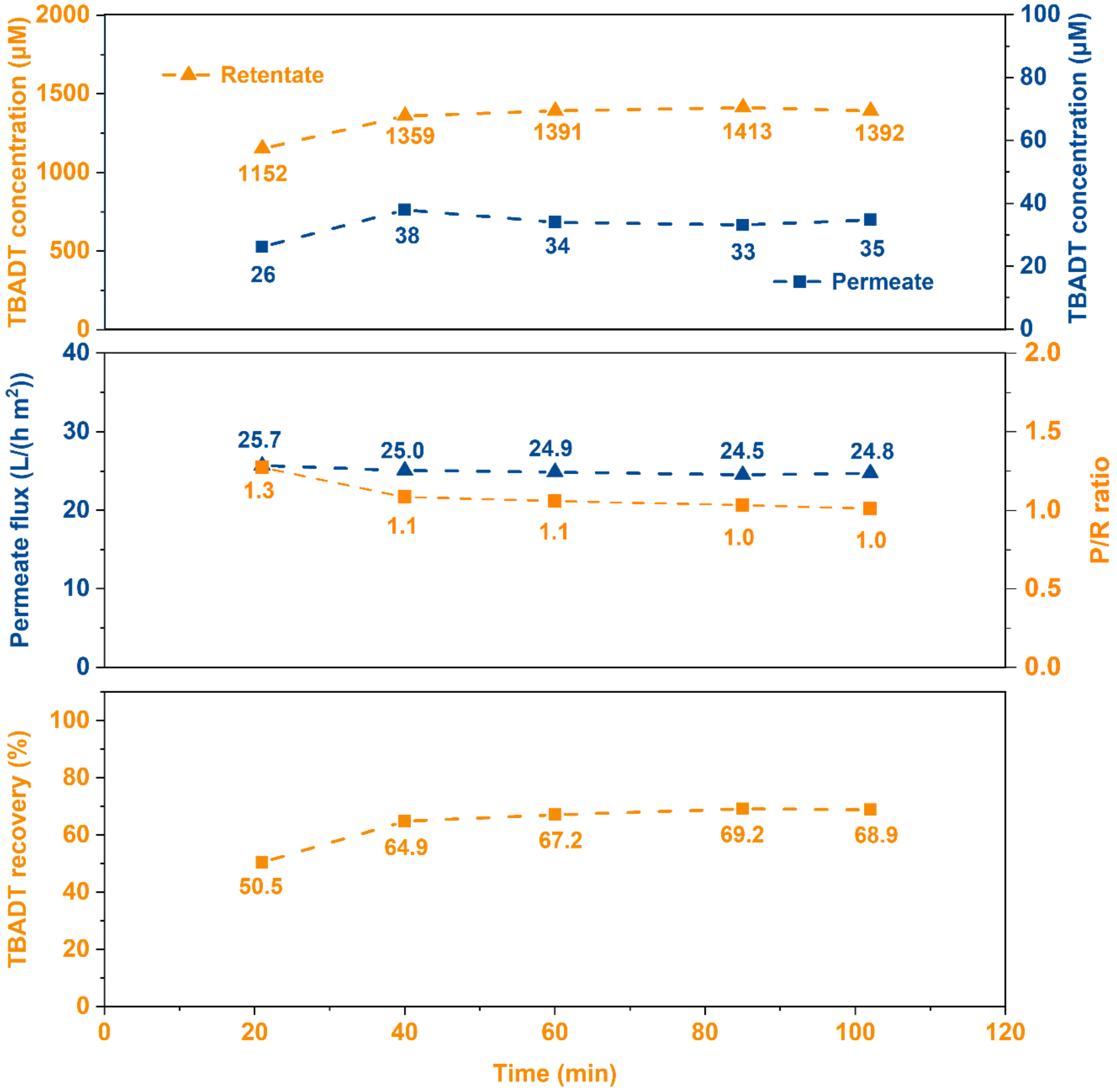

Supplementary Figure 8. Summary of TBADT filtration experiment with SolSep. NF080105 membrane. Experiment condition: 0.25 mL/min input flow rate, 14 bar, 1 mM TBADT solution.

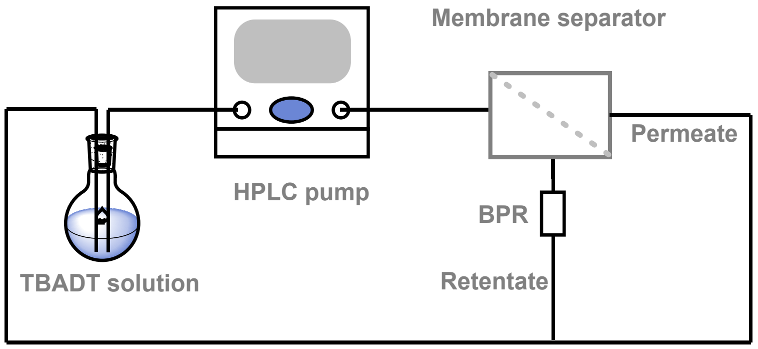

Supplementary Figure 9. Scheme of the setup to test if the TBADT stuck inside the membrane or not.

To verify the presence of catalyst accumulation in the NF080105 membrane, the experimental design depicted in **Supplementary Figure 9** was realized to recirculate the TBADT solution in the setup. After around 4-hours experiment, the concentration of TBADT of inlet solution decreased to around 500 µM, almost half of the initial concentration (See **Supplementary Figure 10**), thus confirming the hypothesis of catalyst loss in the membrane.

The same experiment was carried out with the SolSep. NF030306 membrane. It is clear to see that the TBADT concentration of the inlet solution remained the same after two-and-half-hour circulation (**Supplementary Figure 11**).

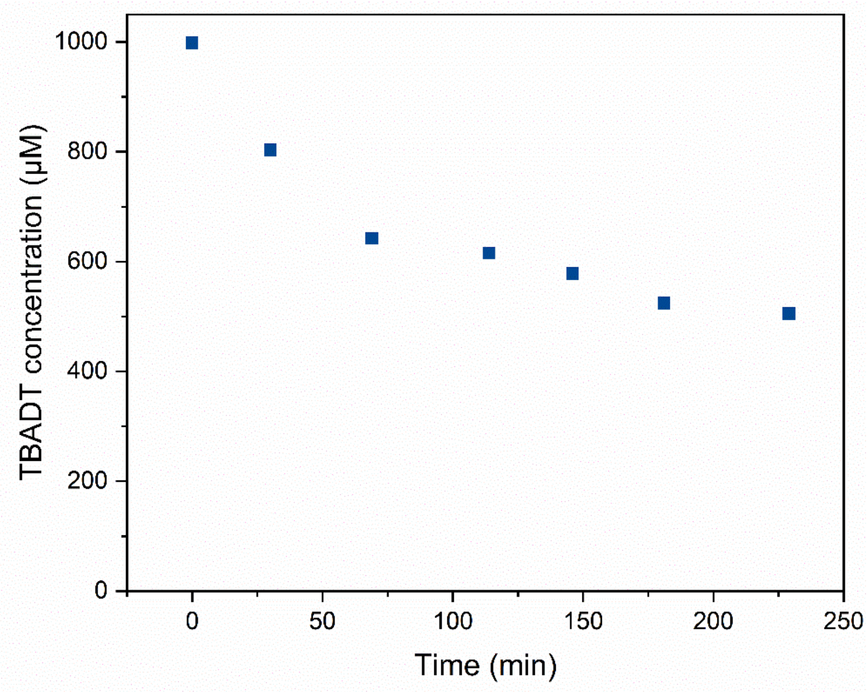

Supplementary Figure 10. Result of the recirculation experiment with Solsep. NF080105 membrane. Experiment condition: 0.25 mL/min recirculation flow rate, 14 bar, 1 mM TBADT solution.

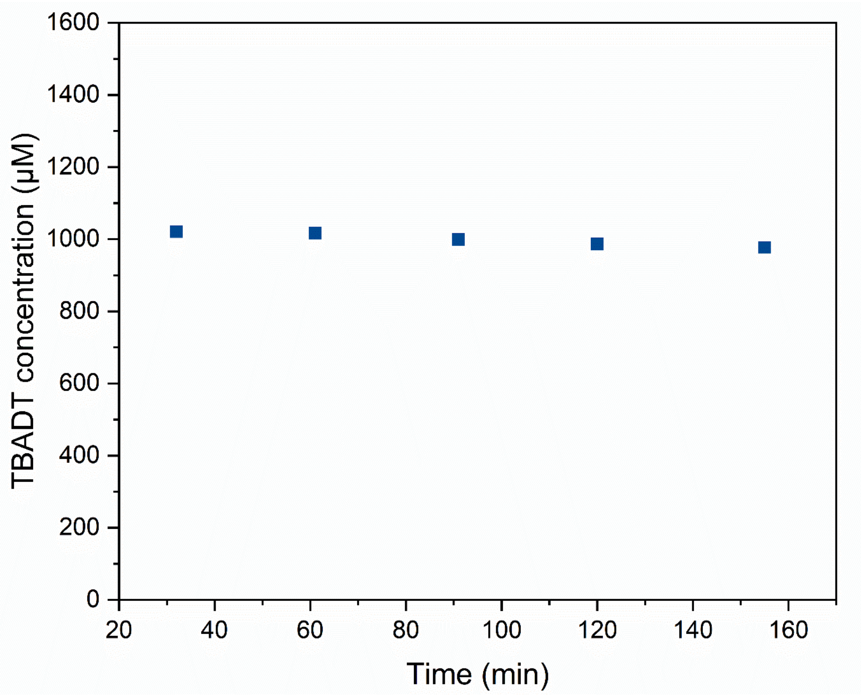

Supplementary Figure 11. Result of the recirculation experiment with Solsep. NF030306 membrane. Experiment condition: 0.25 mL/min recirculation flow rate, 14 bar, 1 mM TBADT solution.

Additionally, the nanofiltration experiment with NF030306 was performed. Although the permeate flux at 14 bar was only 4.3 $L{\cdot}h^{-1}{\cdot}m^{-2}$, the measured TBADT recovery was outstanding and approached ideal selectivity (**Supplementary Figure 12**). Another experiment with 19 bar offered us a 9.0 $L{\cdot}h^{-1}{\cdot}m^{-2}$ permeate flux, which indicated higher operating pressure is beneficial to the nanofiltration process. Considering the maximum operating pressure of Zaiput SEP-10 separator is 20 bar, we decided to design a membrane separator which can work with a high pressure (40 bar).

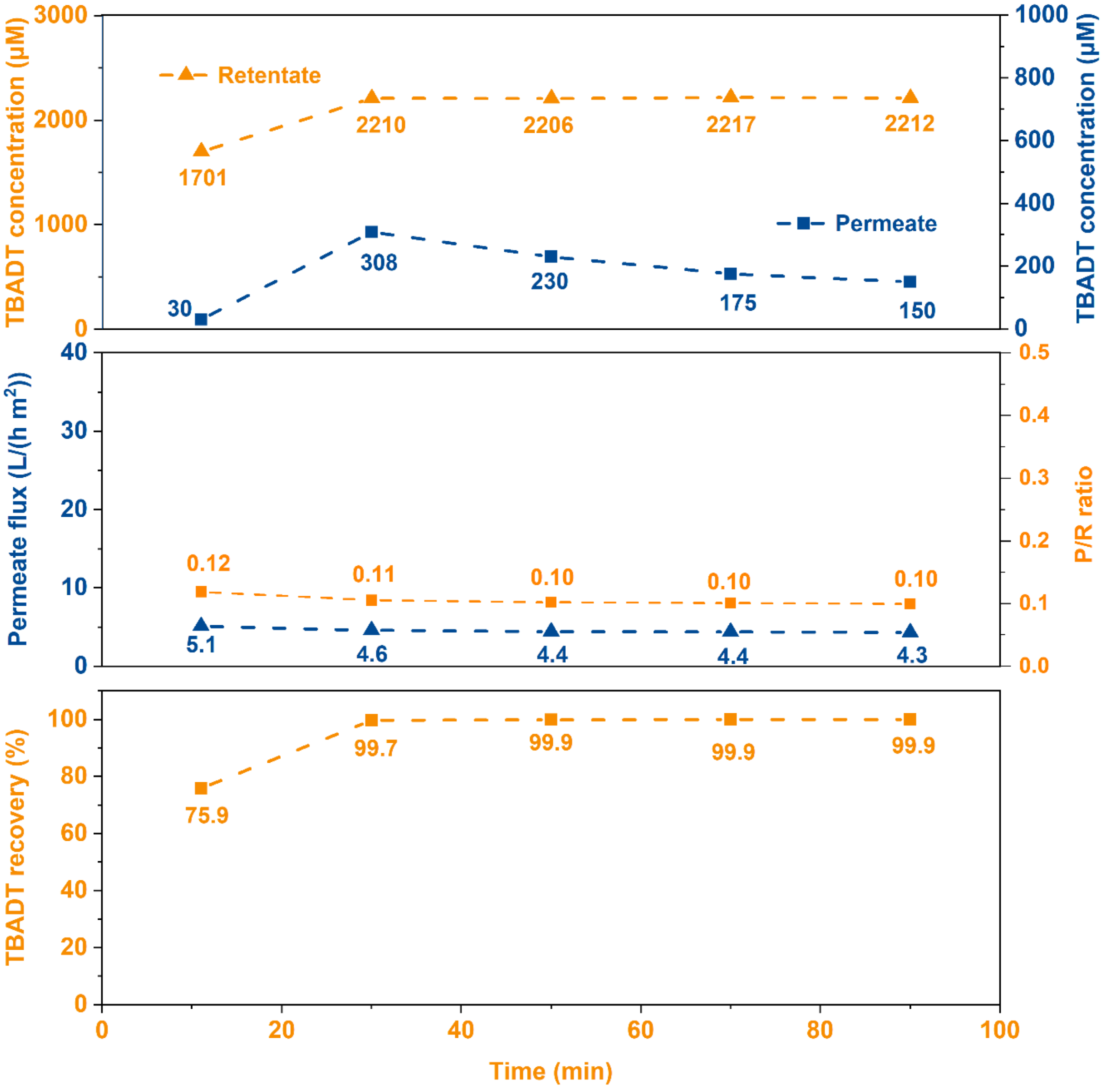

Supplementary Figure 12. Summary of TBADT filtration experiment with Solsep NF030306 membrane. Experiment condition: 0.25 mL/min input flow rate, 14 bar, 2 mM TBADT solution.

2.6 High-pressure membrane separator design

All the nanofiltration experiments with higher pressure (>20 bar) and inline nanofiltration were performed with our high-pressure membrane separator (**Supplementary Figure 13**), which was designed in-house. The cross-flow separator was made of stainless steel 316L and sealed with two EPDM O-rings. The pressure was applied on the retentate side with an IDEX back pressure regulator (BPR). The retentate-side volume of separator was 293 μL with a membrane area being only 2.35 cm^2. The detailed design is shown in **Supplementary Figure 14-16**.

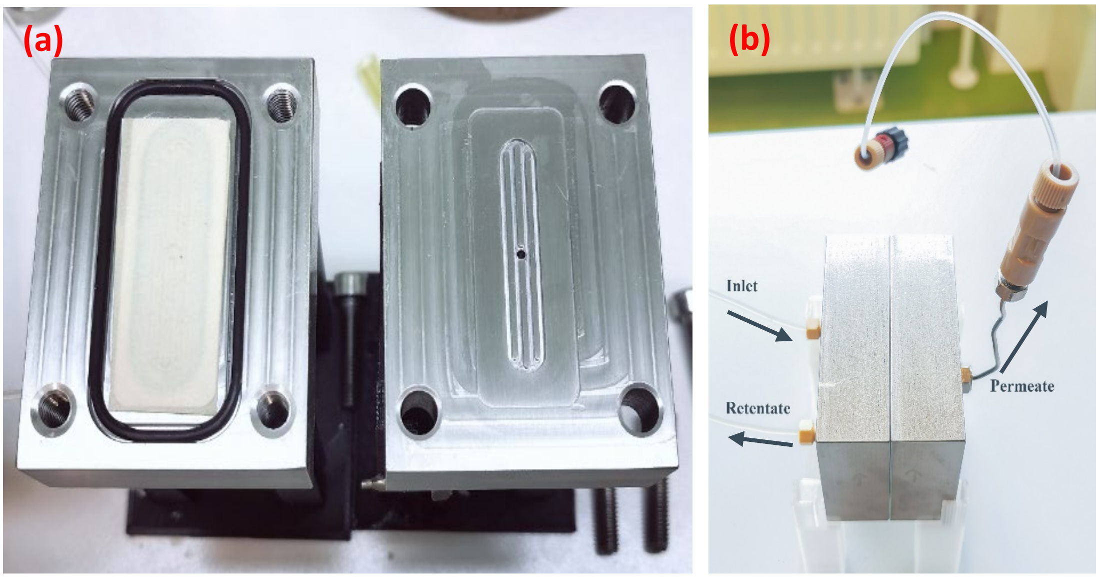

Supplementary Figure 13 Picture of homemade high-pressure membrane separator. (a) The picture of inner structure with nanofiltration membrane. (b) Picture of assembly high-pressure membrane separator.

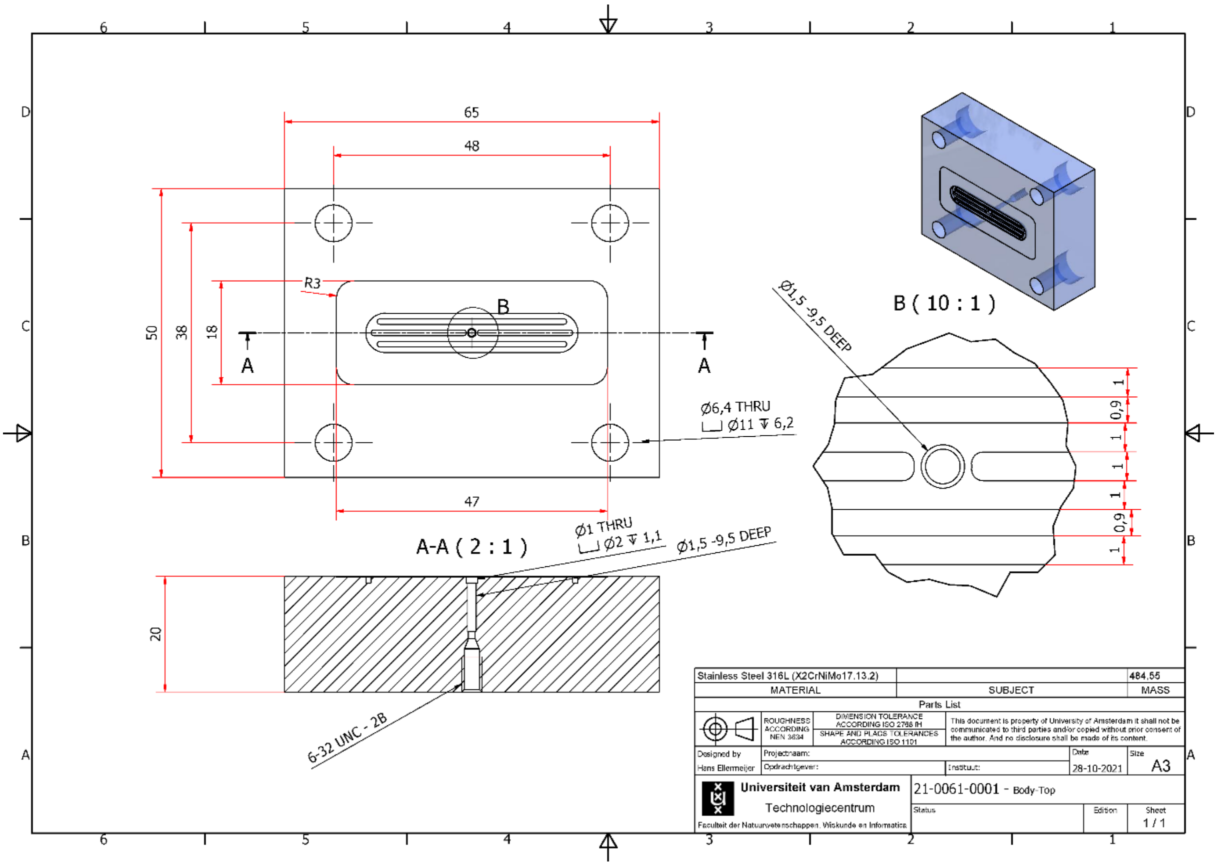

Supplementary Figure 14. Drawing of the permeate side of high-pressure membrane separator.

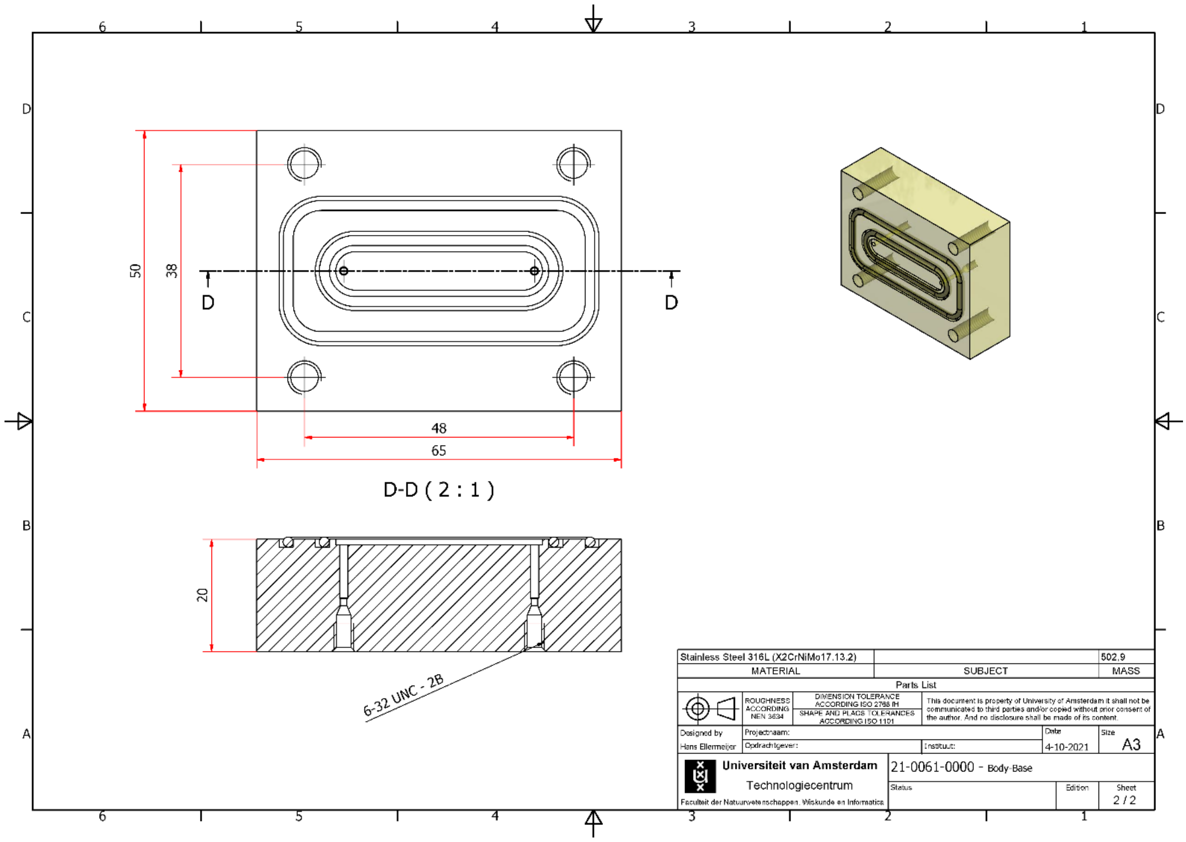

Supplementary Figure 15. Drawing of the retentate side of high-pressure membrane separator.

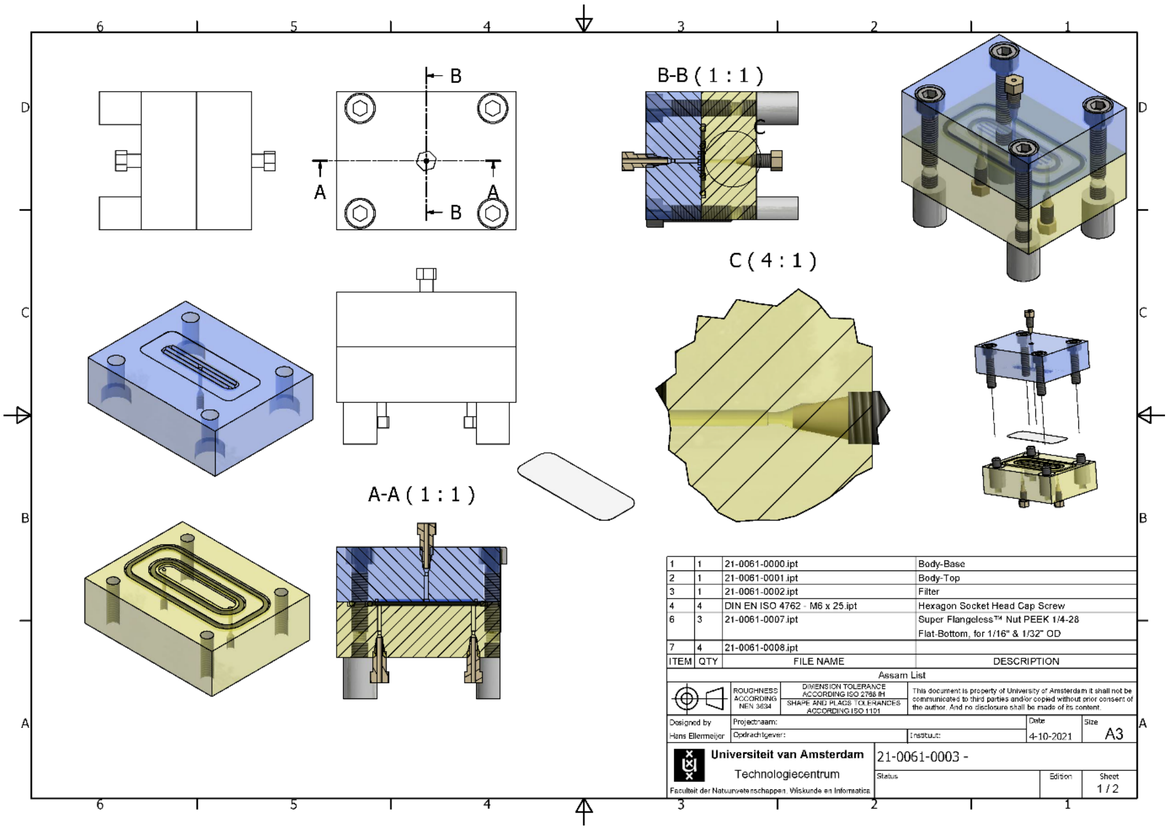

Supplementary Figure 16. Assembly instruction for the high-pressure membrane separator.

2.7 Operating pressure screening

With the high-pressure membrane separator in hand, we screened the operating pressure from 20 bar to 40 bar. The result was presented in **Supplementary Figure 17**. With the increasing operating pressure, the permeate flux increased from 15.3 $L \cdot h^{-1} \cdot m^{-2}$ to 24.4 $L \cdot h^{-1} \cdot m^{-2}$, without any membranes being broken through. However, there were some TBADT precipitated out (see **Supplementary Figure 18**) when working with 40 bar pressure which is the operating pressure limit for the NF030306, according to its specifications. Any fluctuations from HPLC pump at that pressure may cause the nucleation of TBADT, leading the TBADT precipitation and lowering the TBADT recovery. For that reason, the operating pressure of 35 bar was selected for further experiments.

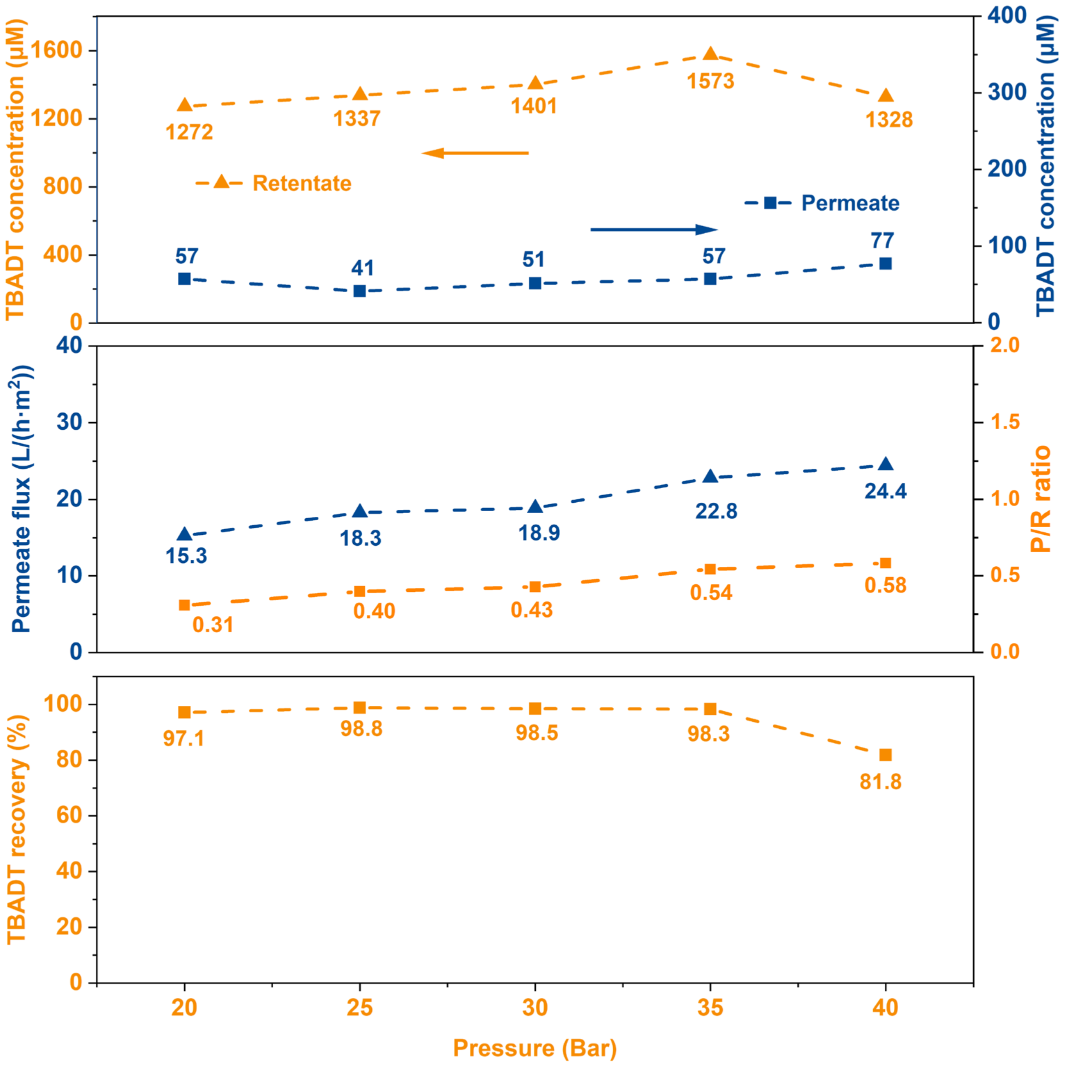

Supplementary Figure 17. Result of operating pressure screening experiment with high-pressure membrane separator. Experiment condition: Solsep NF030306 membrane, 0.25 mL/min input flow rate, 20-40 bar, 1 mM TBADT solution.

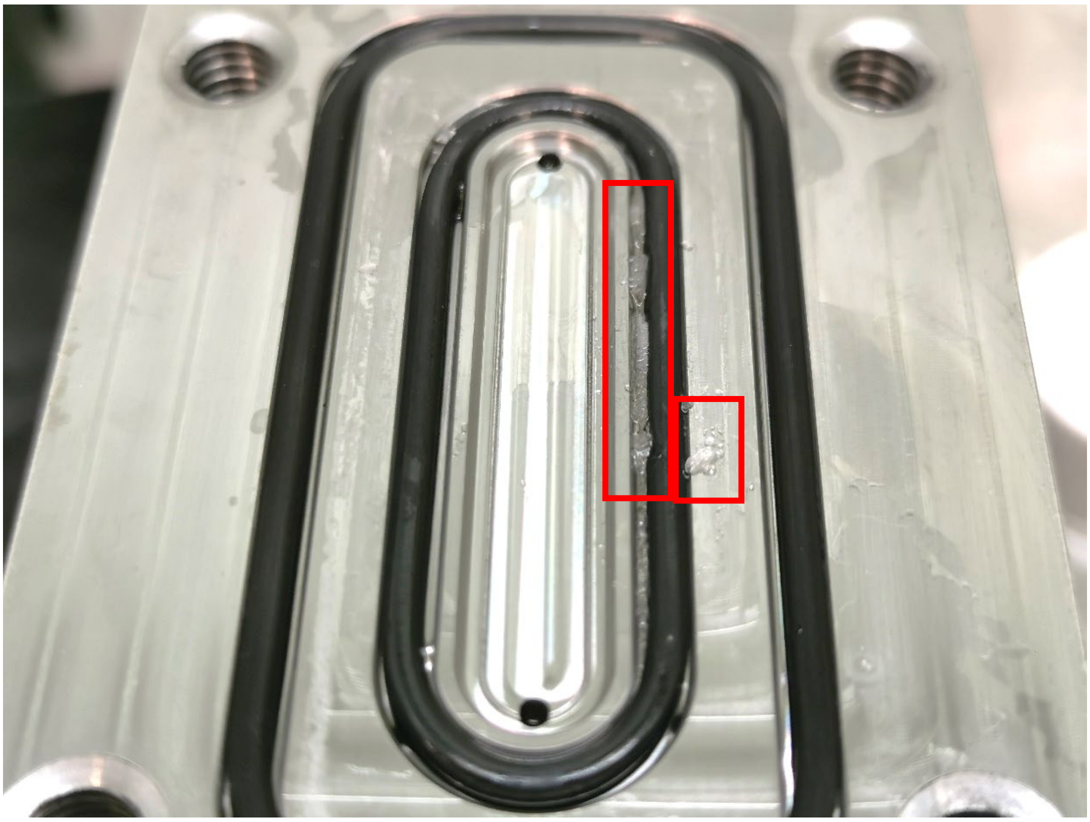

Supplementary Figure 18. Picture of opened membrane separator with TBADT precipitation occurring with 40 bar operating pressure.

2.8 Input flow rate screening

With the operating pressure of 35 bar settled, a series of input flows rates from 0.1 mL/min to 0.3 mL/min were evaluated (see **Supplementary Figure 19**). A lower input flow rate equals to a longer residence time in the separator, leading to a higher permeate-retentate volumetric ratio, which also minimized the amount of product coming back to the photoreactor. However, a decreased flux was observed for the lowest flow rate, which can be explained by reduced mixing in the cell and likely concentration polarization across the membrane. Meanwhile, although slightly higher input flow rate resulted more permeate flux, the amount of product coming back to the reactor also increased in this case. Therefore, the flow rate for catalyst recovery was fixed at 0.15 mL/min.

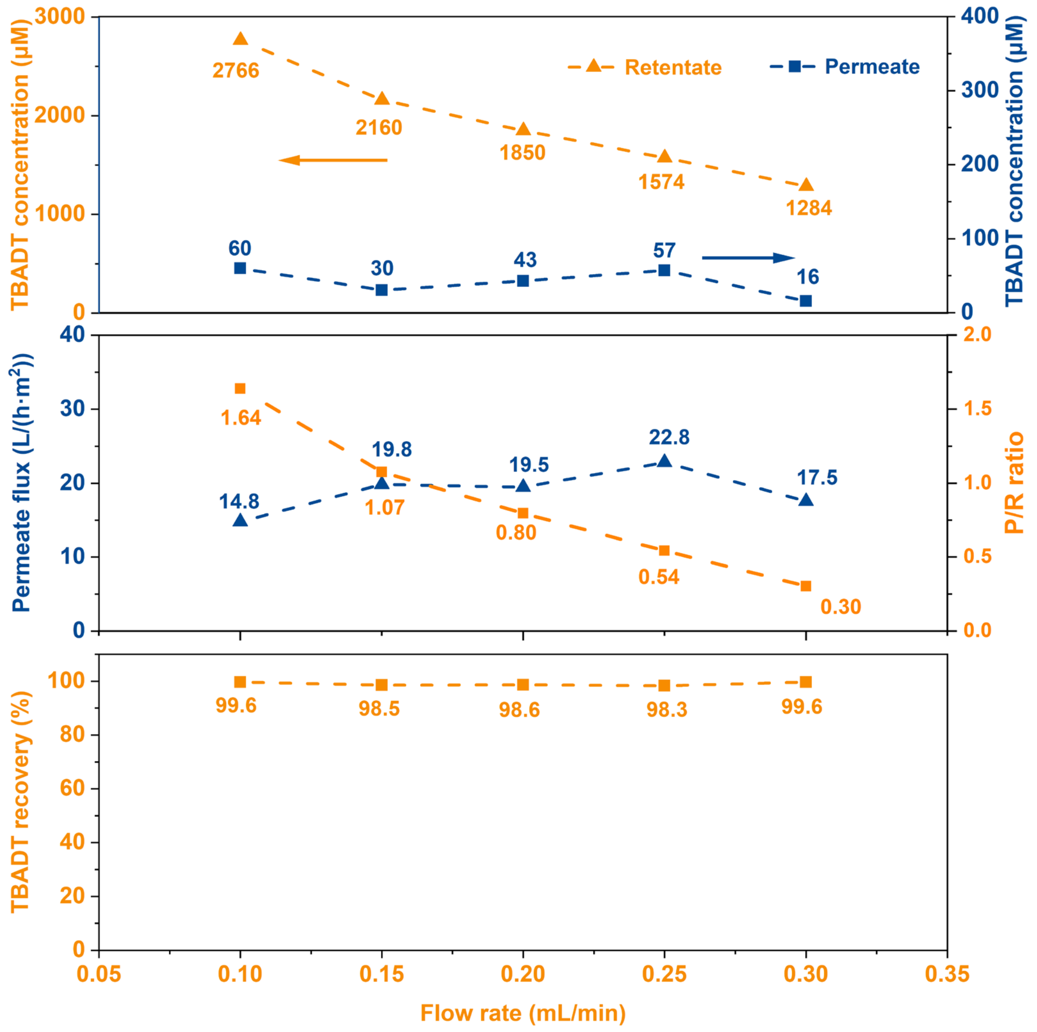

Supplementary Figure 19. Result of input flow rate screening experiment with high-pressure membrane separator. Experiment condition: Solsep NF030306 membrane, 0.1-0.3 mL/min input flow rate, 35 bar, 1 mM TBADT solution.

2.9 TBADT stability test

The potential negative impact on catalyst activity of the recycling process was taken into consideration and tested by performing alkylation reactions with TBADT that was obtained either via OSN or via non-solvent extraction and filtration. The C(sp^3)-H alkylation reaction was shown below. Firstly, the TBADT was filtered by drying the reaction solution in vacuum and then adding ethyl acetate. The recycled TBADT was tested 5 times without any noticeable loss of photocatalytic activity (**Supplementary Figure 20**). Besides, another experiment was done with the recycled TBADT via OSN. As presented in **Supplementary Table 2**, the product yield is comparable to the one achieved with pristine TBADT, which confirmed that the recycle process has no negative effect on TBADT activity.

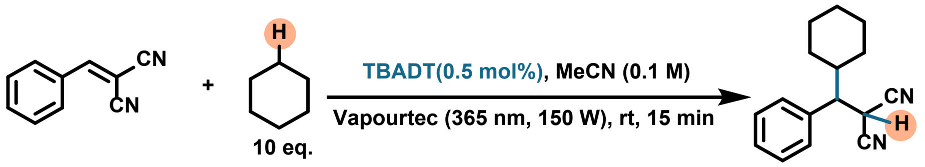

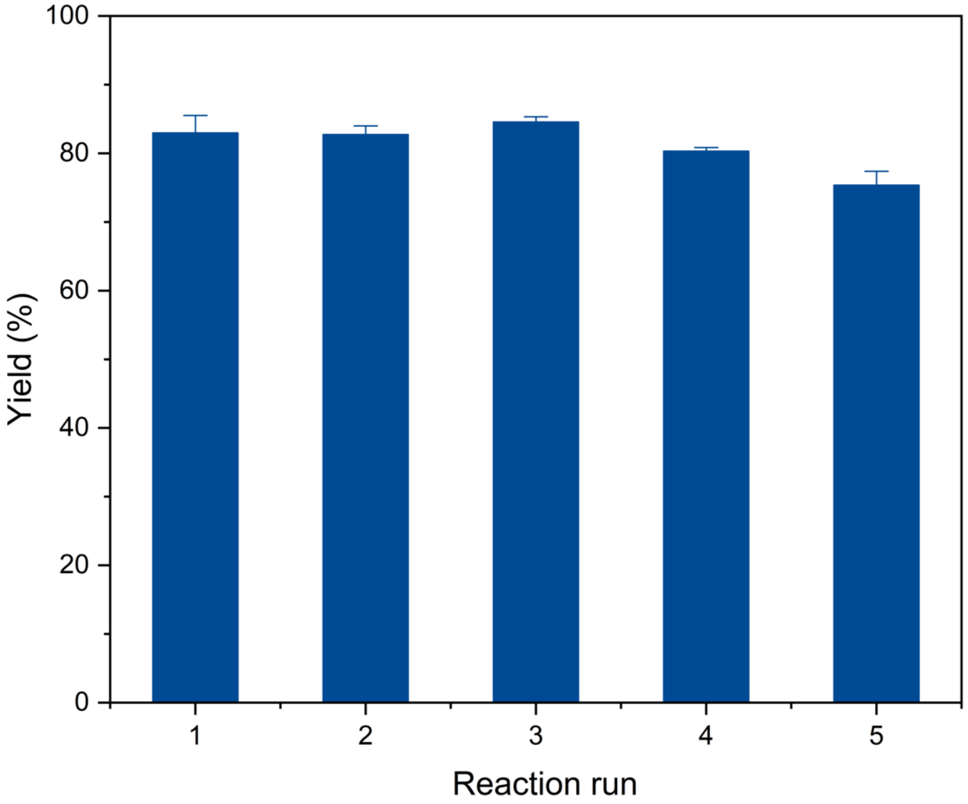

Supplementary Figure 20. TBADT stability test with recycled TBADT via non-solvent extraction and filtration. Reaction condition: 2-benzylidenemalononitrile (0.1 M), cyclohexane (10 equiv), TBADT (0.5 mol %) in CH_3CN. Irradiation source: λ = 365 nm (150 W), residence time: 15 min.

Supplementary Table 2. TBADT stability test with recycled TBADT via OSN [a]

Entry	Condition	Conversion (%) [b]	Yield (%) [c]
1	Fresh TBADT	100	86
2	Recycled TBADT from OSN [d]	100	82

[a] Reaction condition: 2-benzylidenemalononitrile (0.1 M), cyclohexane (10 equiv), TBADT (0.5 mol%) in CH_3CN. Irradiation source: λ = 365 nm (150 W), residence time: 15 min. [b] The conversion of dimethyl maleate was determined by ^{1}H-NMR using 1,2,4,5-tetramethylbenzene as external standard. [c] The yield of product was determined by ^{1}H-NMR using 1,2,4,5-tetramethylbenzene as external standard. [d] The OSN was performed with NF030306 membrane in high-pressure flow cell (35 bar).

2.10 Potentially undesired effect of membrane-reactant interactions

As the screening experiments performed above were carried out with single TBADT solution, we still need to see if the reactants may affect the nanofiltration process or not. Different inlet solutions, 1 mM TBADT solution mixed with 0.4 M. Cyclohexane solution and a reaction solution with dimethyl maleate (see **Section 3**), were injected to the separator via the HPLC pump. Permeate flux of the membrane was calculated to monitor the nanofiltration performance. It is evident to see from **Supplementary Figure 21** that comparable flux values were obtained with different inlet solution. Besides, almost no selection of dimethyl maleate and product over the membrane were observed during the nanofiltration experiment of reaction solution (**Supplementary Figure 22**).

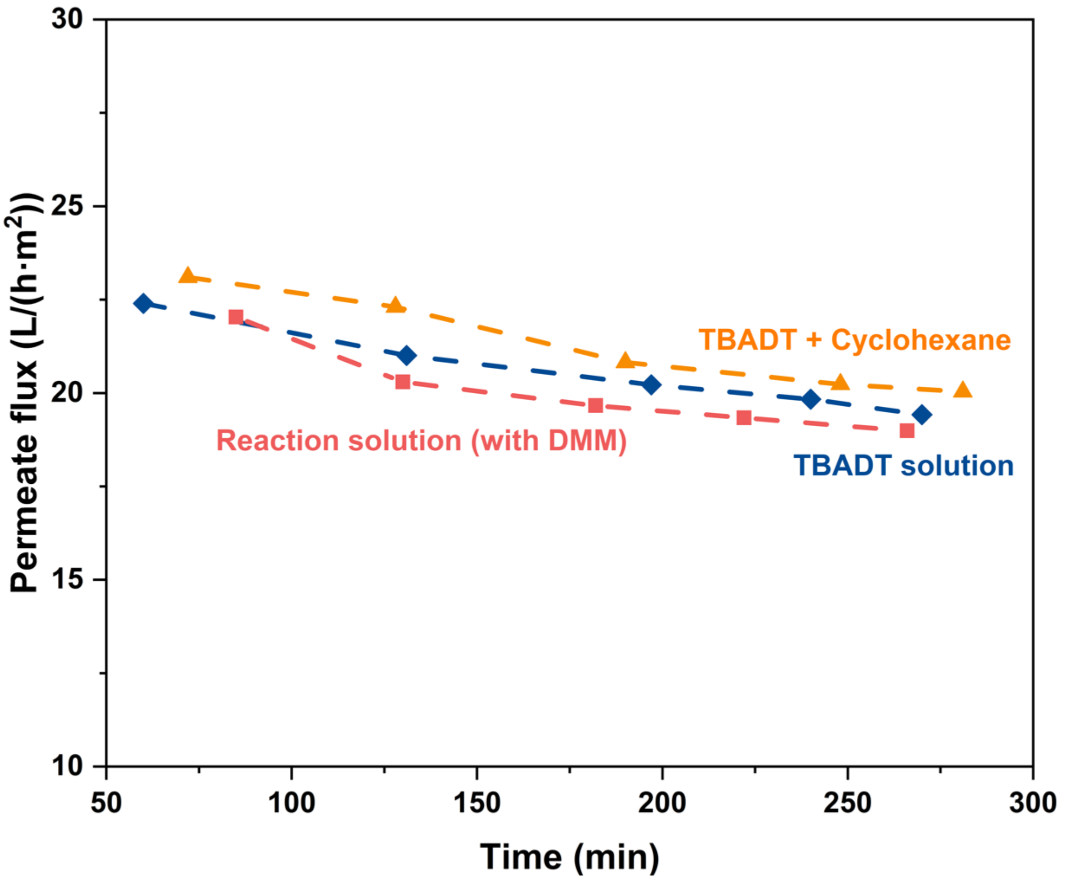

Supplementary Figure 21. Permeate flux of nanofiltration experiments with different inlet solution. Experiment condition: Solsep NF030306 membrane, 0.15 mL/min input flow rate, 35 bar, 1 mM TBADT solution (blue curve), 1 mM TBADT solution + 0.4 M cyclohexane solution (orange curve) and reaction solution with Dimethyl maleate (DMM) (magenta curve).

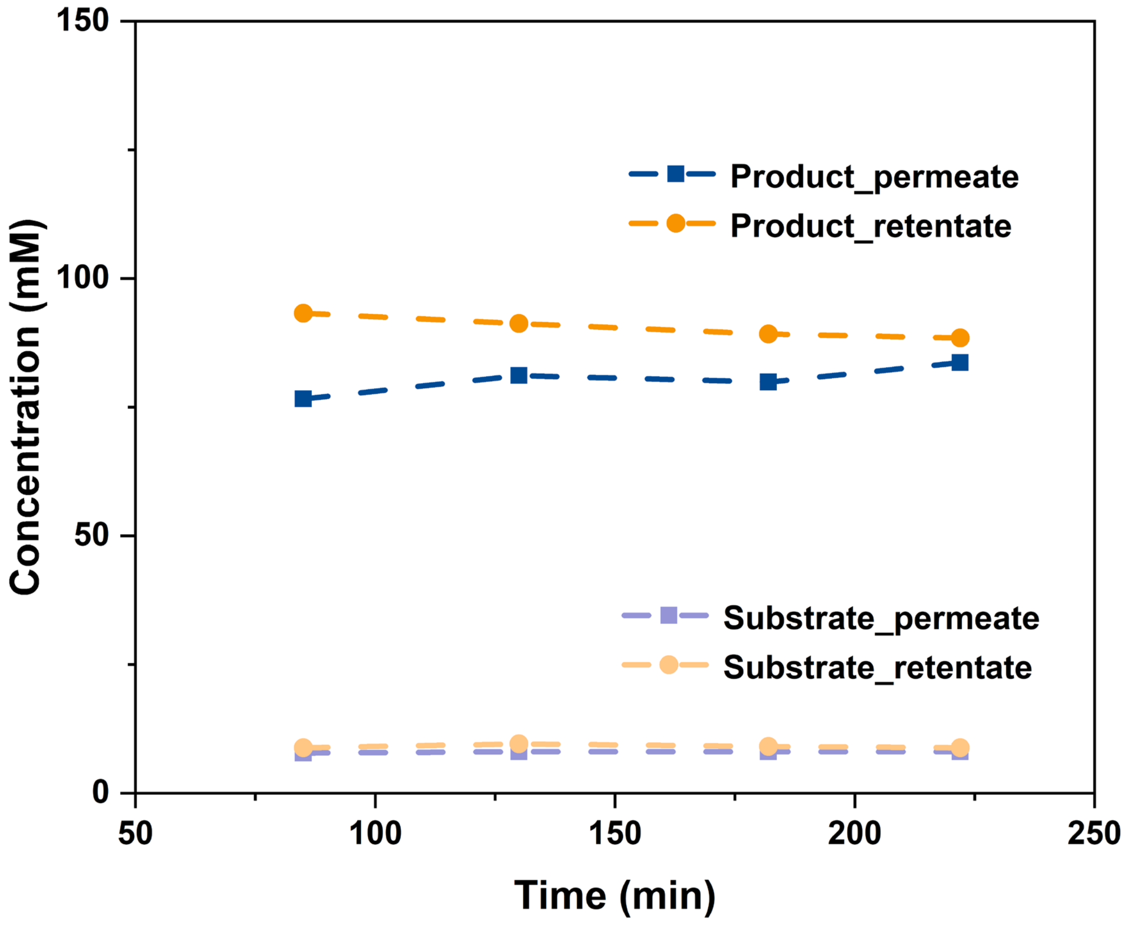

Supplementary Figure 22. Concentrations of dimethyl maleate (Substrate) and product in permeate and retentate solutions. Experiment condition: Solsep NF030306 membrane, 0.15 mL/min input flow rate, 35 bar, reaction solution with dimethyl maleate.

2.11 Estimation of how many NF stages needed to reach 95% product recovery

Considering the concentrations of product in permeate and retentate solution are almost the same, we only need to calculate the total permeate volume over the inlet solution volume. Let's assume that the P/R ratio is constant and take the P/R ratio we obtained at the first stage for the derivation of the number of stages required to achieve 95% product recovery. The P/R ratio is 0.86. After first stage, we obtain 0.86 of permeate and 1 of retentate. And then we will obtain$1 * (1/(1+0.86))^{N-1}$of retentate and $0.86 * (1/(1+0.86))^{N-1}$ of permeate at the Nth stage. We want that $\sum_1^N 0.86 * (1/(1+0.86))^{N-1} > 0.95 * 1.86$, so N should be larger than 5, which means we need 5 stage recirculation to obtain 95% recovery of product. In reality, the number may be lower given the increase in P/R for subsequent stages.

3. General reaction procedure

3.1 General procedure 1 (GP1) for single run of $C(sp^3)$-H alkylation:

A 5 mL volumetric flask was charged with olefin (0.1 M, 0.5 mmol, 1 equiv.), H-donors (1 M, 5 mmol, 10 equiv.) and tetrabutylammonium decatungstate (TBADT) (1 mM, 5 µmol, 1 mol%). Next, CH_3CN was added to acquire a total volume of 5 mL. The liquid was taken up with a syringe, which was then mounted on a syringe pump. The syringe was directly connected to a 3.06 mL Vapourtec Reactor (PFA capillary tubing, 750 µm inner diameter). Stock solution feed was pumped into the flow reactor with 0.153 mL/min flow rate, corresponding to 20 minutes residence time. When the syringe was fully empty, again CH_3CN was loaded into another syringe and injected to collect all product at the end of the reactor in a flask. The solvent was removed under reduced pressure and purified by flash column chromatography on a Biotage® Isolera Four system affording the product, which was characterized by ^{1}H NMR and ^{13}C NMR.

3.2 General procedure 2 (GP2) for single run of $C(sp^3)$-H amination:

A 5 mL volumetric flask was charged with diisopropyl azodicarboxylate (0.2 M, 1 mmol, 1 equiv.), tetrahydrofuran (2 M, 10 mmol, 10 equiv.) and tetrabutylammonium decatungstate (TBADT) (0.8 mM, 4 µmol, 0.4 mol%). Next, CH_3CN was added to acquire a total volume of 5 mL. The liquid was taken up with a syringe, which was then mounted on a syringe pump. The syringe was directly connected to a 3.06 mL Vapourtec Reactor (PFA capillary tubing, 750 µm inner diameter). Stock solution feed was pumped into the flow reactor with 0.153 mL/min flow rate, corresponding to 20 minutes residence time. When the syringe was fully empty, again CH_3CN was loaded into another syringe and injected to collect all product at the end of the reactor in a flask. The solvent was removed under reduced pressure and purified by flash column chromatography on a Biotage® Isolera Four system affording the product, which was characterized by ^{1}H NMR and ^{13}C NMR.

3.3 General procedure 3 (GP3) for inline nanofiltration:

The general procedure for inline nanofiltration experiment follows several steps below:

1. Assembly the membrane separator unit.
2. Integrate the photoreactor with the membrane separator unit.
3. Pump pure CH_3CN to the system until the desired pressure (35 bar) was reached.

4. Prepare a 50 mL reaction solution according to **GP1/2** and then transfer the solution to holding tank.
5. Start the reaction, collect the samples from permeate (P1 and P2) and retentate (R) and measure the TBADT concentration in the solutions until reach the equilibrium of the membrane (**Supplementary Figure 23**).
6. Prepare the substrate solution with H-donor inside, transfer it to an erlenmeyer flask and lower the volume of solution inside holding tank to 1 mL.
7. Put the retentate feed back to the holding tank. Meanwhile, start delivering substrate solution with Peristaltic pump (**Supplementary Figure 24**).
8. Collect the samples from two permeate feeds and analyse them with ^{1}H NMR to calculate the TON in permeate. The flow rate of the substrate solution and permeate flux were measured based on the weights of substrate solution pumped and permeate solutions collected at a certain time interval, respectively.
9. After certain experiment time, use pure CH_3CN to push out the solution left in the system and analyse the solution with ^{1}H NMR to calculate the TON in system.

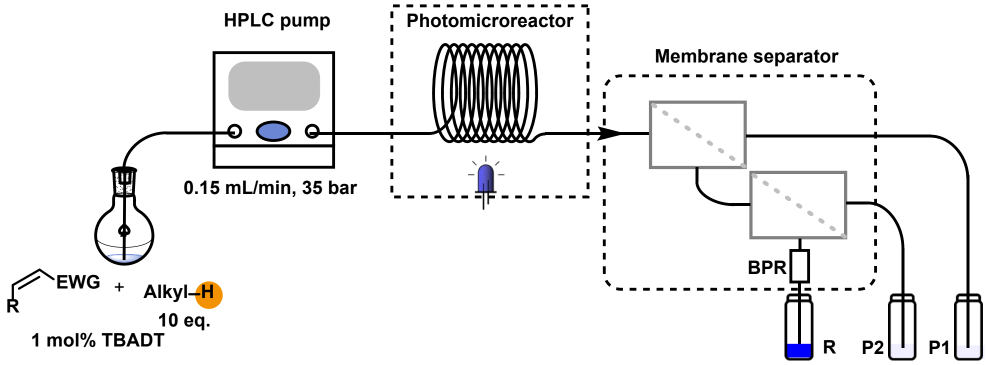

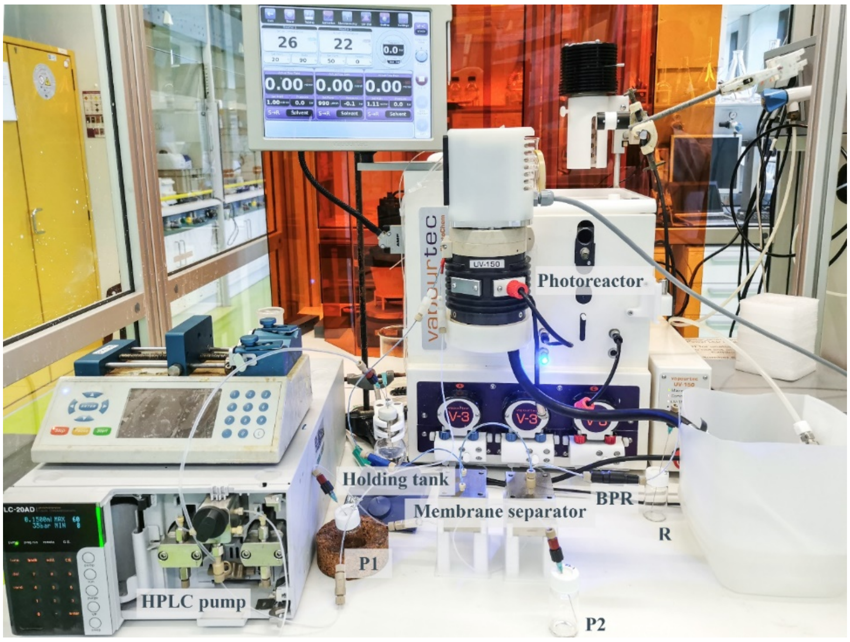

Supplementary Figure 23. Scheme (top) and picture (bottom) of the setup for membrane equilibrium.

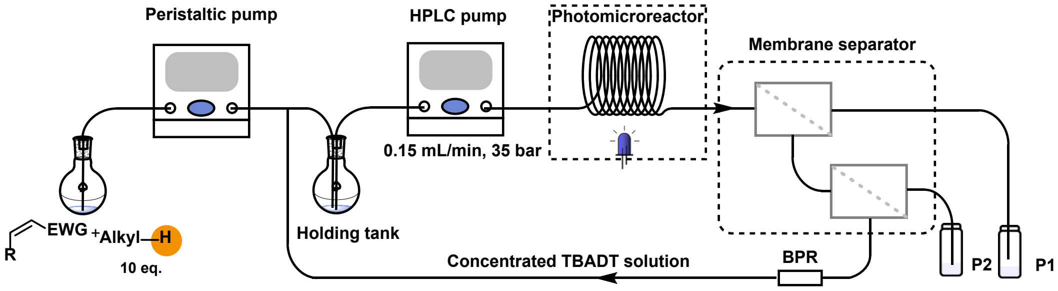

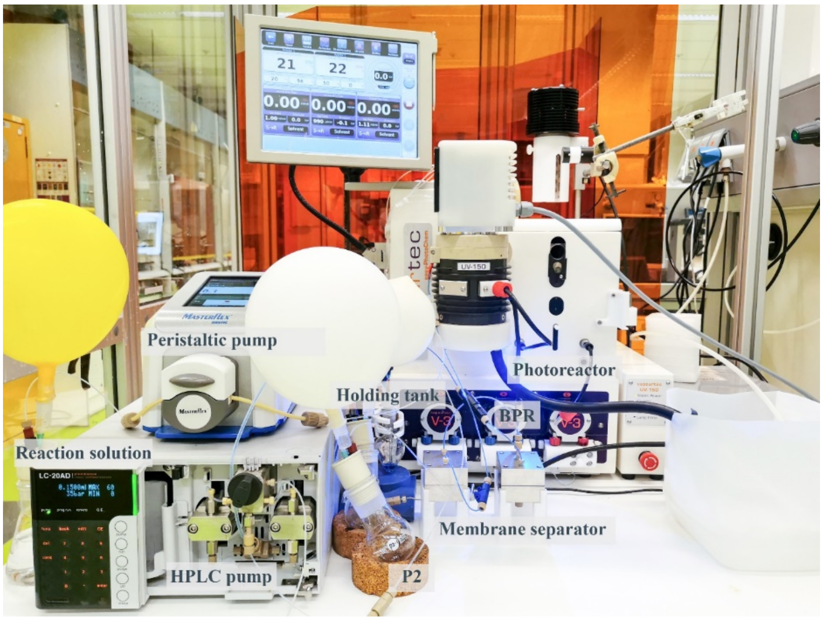

Supplementary Figure 24. Scheme (top) and picture (bottom) of the setup for inline nanofiltration.

4.Reaction Optimization

4.1 General experimental procedure for screening alkylation reaction:

The optimization of the reaction conditions was carried out by studying the alkylation of cyclohexane onto dimethyl maleate to give dimethyl 2-cyclohexylsuccinate on a 0.5 mmol scale.

Following the **GP1** (see section 3.1), a 5 mL volumetric flask was charged with dimethyl maleate (0.1 M, 0.5 mmol, 1 equiv.), cyclohexane and tetrabutylammonium decatungstate (TBADT). Next, CH_3CN was added to acquire a total volume of 5 mL. The stock solution feed was pumped into the flow reactor at the needed flow rate. After 1.5 reactor volume, a steady state was assumed and an aliquot of the outflow (100 µL) was collected, diluted with 200 µL CD_3CN along with 100 µL 1,2,4,5-tetramethylbenzene solution (in CH_3CN) as external standard. And the mixture was analysed by ^1H NMR to calculate the conversion and yield. For a representative NMR spectrum, see **Supplementary Figure 25**.

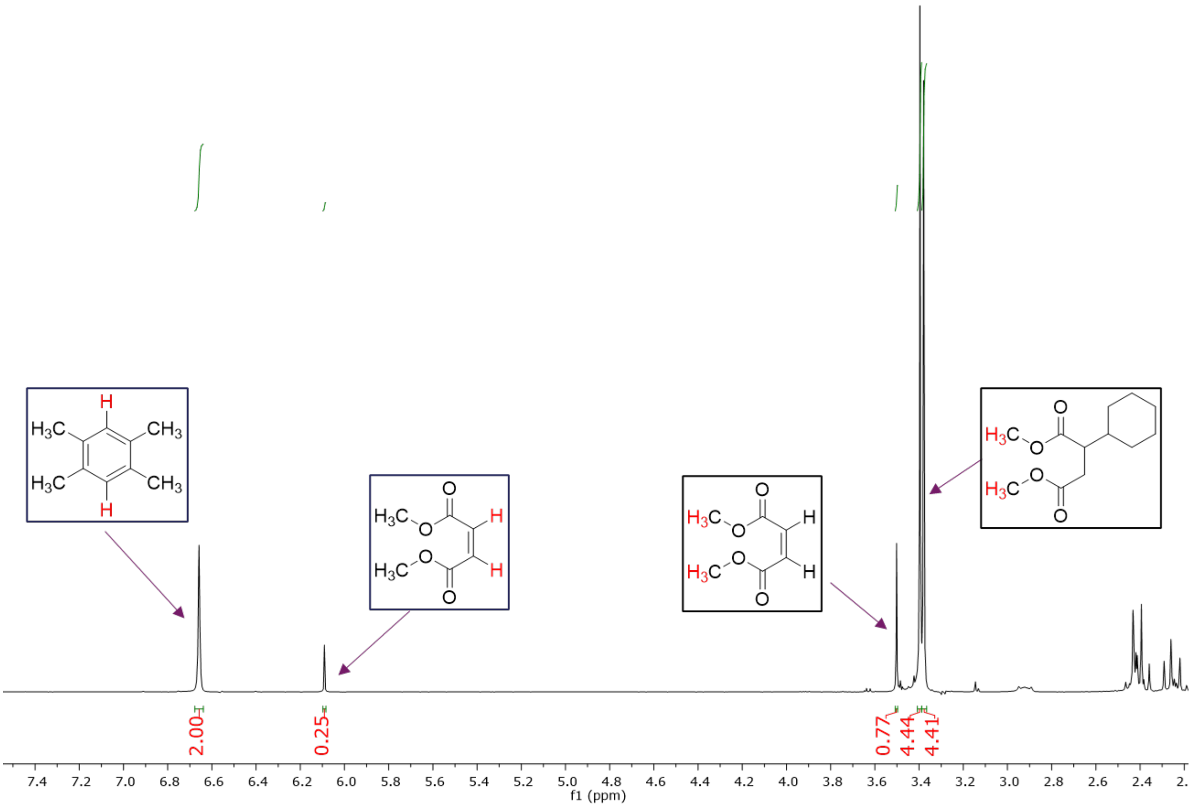

Supplementary Figure 25. Example of ^1H-NMR spectra for determining NMR yields.

4.2 TBADT loading screening

Supplementary table 3. TBADT loading screening [a]

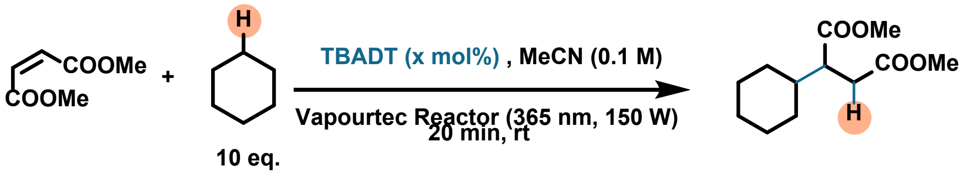

Entry	TBADT loading (mol%)	Conversion (%)[b]	Yield (%)[c]
1	1	93	87
2	0.5	83	70
3	0.2	52	40
4	-	7	0

[a] Reaction conditions (0.5 mmol scale): Dimethyl maleate (0.1 M), cyclohexane (10 equiv), TBADT (x mol %) in CH_3CN (5 mL). Irradiation source: λ = 365 nm (150 W), residence time: 20 min. [b] The conversion of dimethyl maleate was determined by ^{1}H-NMR using 1,2,4,5-tetramethylbenzene as external standard. [c] The yield was determined by ^{1}H-NMR using 1,2,4,5-tetramethylbenzene as external standard.

4.3 Cyclohexane equivalent screening

Supplementary table 4. Cyclohexane equivalent screening [a]

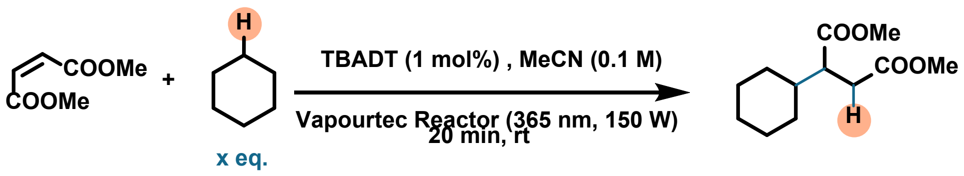

Entry	Cyclohexane loading (equiv.)	Conversion (%)[b]	Yield (%)[c]
1	10	93	87
2	7	87	79
3	5	79	73
4	3	72	51

[a] Reaction conditions (0.5 mmol scale): Dimethyl maleate (0.1 M), cyclohexane (x equiv), TBADT (1 mol %) in CH_3CN (5 mL). Irradiation source: λ = 365 nm (150 W), residence time: 20 min. [b] The conversion of dimethyl maleate was determined by ^{1}H-NMR using 1,2,4,5-tetramethylbenzene as external standard. [c] The yield was determined by ^{1}H-NMR using 1,2,4,5-tetramethylbenzene as external standard.

4.4 Residence time screening

Supplementary table 5. Residence time screening [a]

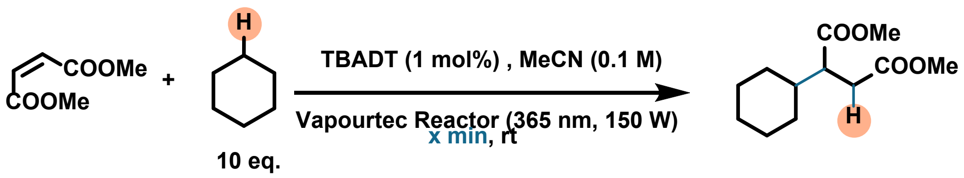

Entry	Residence time (min)	Conversion (%)[b]	Yield (%)[c]
1	30	96	94
2	20	93	87
3	15	89	72
4	10	68	50

[a] Reaction conditions (0.5 mmol scale): Dimethyl maleate (0.1 M), cyclohexane (10 equiv), TBADT (1 mol %) in CH_3CN (5 mL). Irradiation source: λ = 365 nm (150 W), residence time: x min. [b] The conversion of dimethyl maleate was determined by ^{1}H-NMR using 1,2,4,5-tetramethylbenzene as external standard. [c] The yield was determined by ^{1}H-NMR using 1,2,4,5-tetramethylbenzene as external standard.

4.5 Pressure screening

Supplementary table 6. Pressure screening [a]

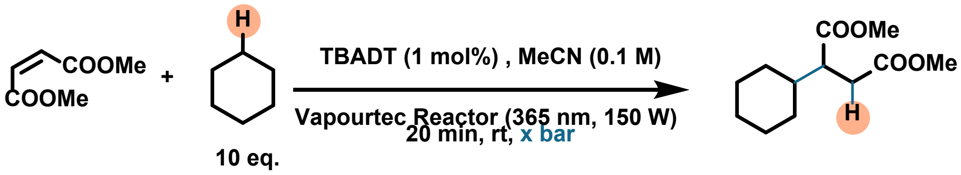

Entry	Reaction pressure (bar)	Conversion (%)[b]	Yield (%)[c]
1	1	93	87
2	35	91	89

[a] Reaction conditions (0.5 mmol scale): Dimethyl maleate (0.1 M), cyclohexane (10 equiv), TBADT (1 mol %) in CH_3CN (5 mL). Irradiation source: λ = 365 nm (150 W), residence time: 20 min, reaction pressure: x bar. [b] The conversion of dimethyl maleate was determined by ^{1}H-NMR using 1,2,4,5-tetramethylbenzene as external standard. [c] The yield was determined by ^{1}H-NMR using 1,2,4,5-tetramethylbenzene as external standard.

4.6 General experimental procedure for screening C(sp^3)-H amination reaction:

The optimization of the reaction conditions was carried out by studying the amination of tetrahydrofuran (THF) onto diisopropyl azodicarboxylate to give corresponded hydrazine on a 0.5-1 mmol scale.

Following the **GP2** (see section 3.2), a 5 mL volumetric flask was charged with diisopropyl azodicarboxylate (0.1-0.2 M, 0.5-1 mmol, 1 equiv.), THF and tetrabutylammonium decatungstate (TBADT). Next, CH_3CN was added to acquire a total volume of 5 mL. The stock solution feed was pumped into the flow reactor at the needed flow rate. After 1.5 reactor volume, a steady state was assumed and an aliquot of the outflow (100 µL) was collected, diluted with 850 µL CH_3CN along with 50 µL Biphenyl (0.1 M in CH_3CN) as external standard. And the mixture was analysed by GC-MS to calculate the yield. For a representative GC-MS spectra, see **Supplementary Figure 26**. A calibration curve (**Supplementary Figure 27**) of product *vs* biphenyl was prepared to calculate the yield.

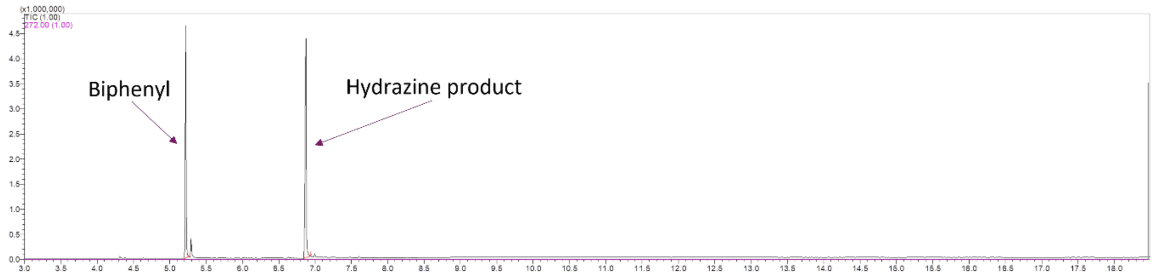

Supplementary Figure 26. Example of GC-MS spectra for determining the yield of hydrazine.

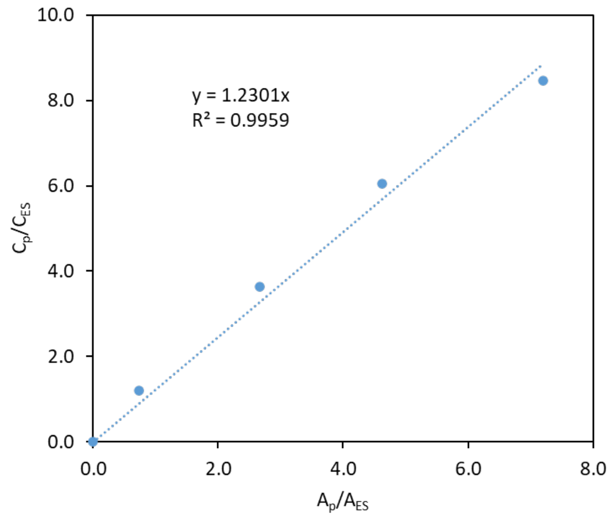

Supplementary Figure 27. Calibration curve of amination product (hydrazine, p) vs. biphenyl (external standard, ES) on GC-MS.

4.7 C(sp³)-H Amination reaction conditions screening

Supplementary table 7. C(sp³)-H Amination reaction conditions screening [a]

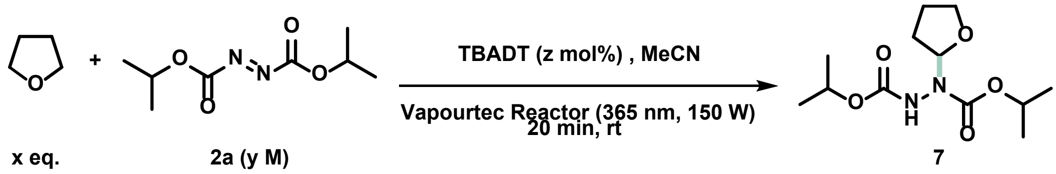

Entry	2a conc. (M)	THF equiv.	TBADT loading (mol%)	Yield[b] (%)
1	0.1	10	1	86
2	0.1	10	0.5	85
3	0.2	10	0.5	87
4	0.2	10	0.4	84 (81)[c]
5	0.2	10	0.2	71
6	0.2	5	0.5	66

[a] Reaction conditions (0.5 - 1 mmol scale): Diisopropyl azodicarboxylate (x M), tetrahydrofuran (y equiv), TBADT (z mol %) in CH_3CN (5 mL). Irradiation source: λ = 365 nm (150 W), residence time: 20 min. [b] The yield was determined by GC-MS using biphenyl as an external standard. [c] Isolated yield is given in parentheses.

5. Long-term experiment of C(sp³)-H alkylation

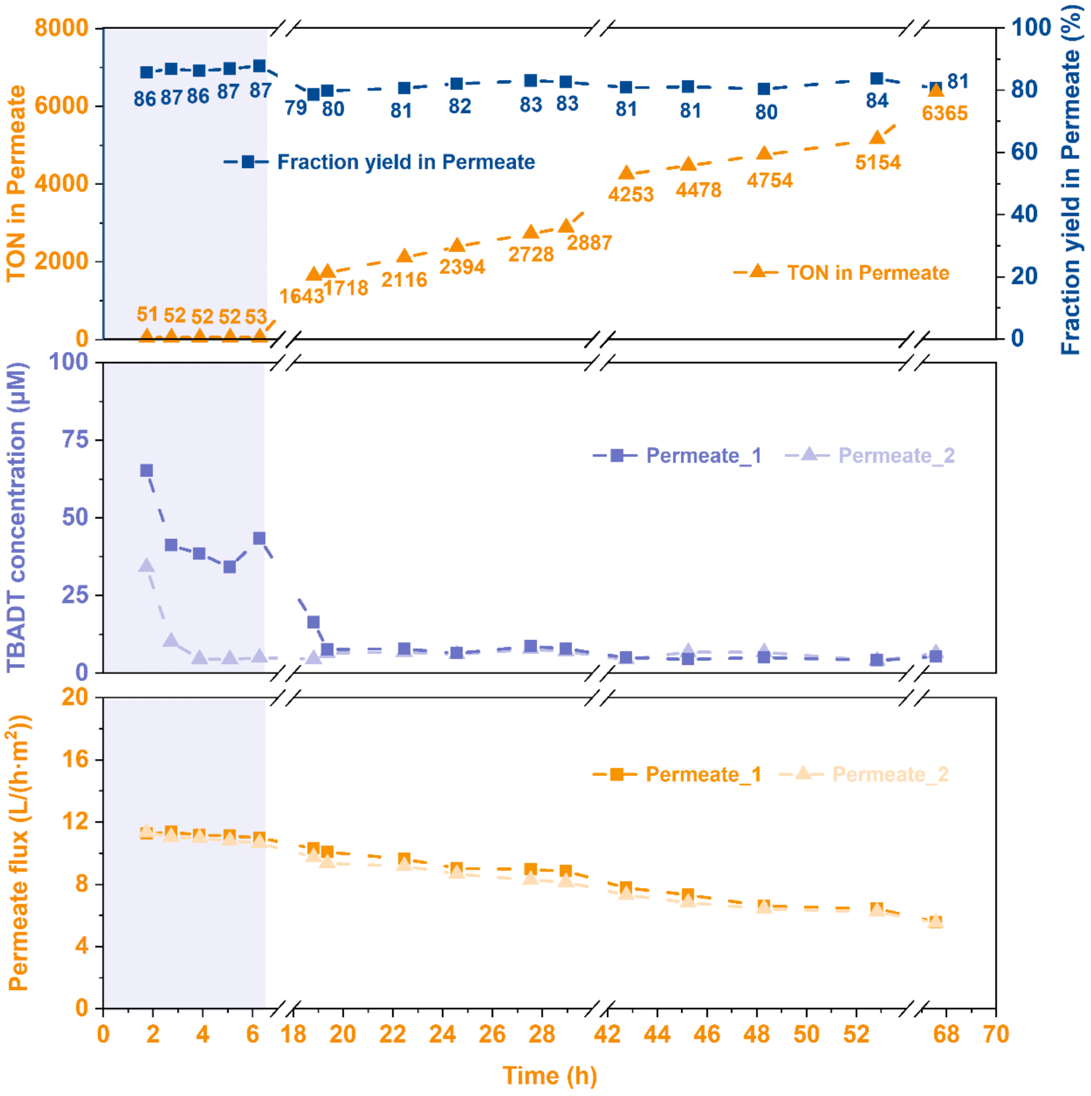

Supplementary Figure 28. Results of a 60-hour run of photocatalytic C(sp^3)-H alkylation with inline TBADT recovery. Light purple area means membrane equilibrium.

Following the **GP3** (see **Section 3.3**), a 60-hour long-term experiment of C(sp^3)-H alkylation was performed to further highlight the potential of this technology. During the whole experiment, a stable fraction yield in permeate (around 81 %) was obtained with the increasing turnover number (TON) over time. After 60.5 h, a TON of 6365 was obtained by counting only the product in the permeate. An additional 373 TONs was obtained by measuring the amount of product remaining in the system after the experiment, giving a total TON of 6738 (5 μmol TBADT used, 33.69 mmol product formed) from this experiment. Besides, the TBADT concentration inside the permeate feeds always stayed at a very low level (~ 5 μM), which provided an excellent TBADT recovery. Additionally, a decreasing permeate flux was

also observed in this long-term experiment, which leads a decreased TOF over time (**Supplementary Figure 29**). One assumption for this is the membrane fouling happened over time, which was confirmed by checking the membrane after the experiment (**Supplementary Figure 30(A)**). Interestingly, the fouling membrane can be easily cleaned with pure acetonitrile (**Supplementary Figure 30(B)**), which indicated a possible solution for future inline nanofiltration.

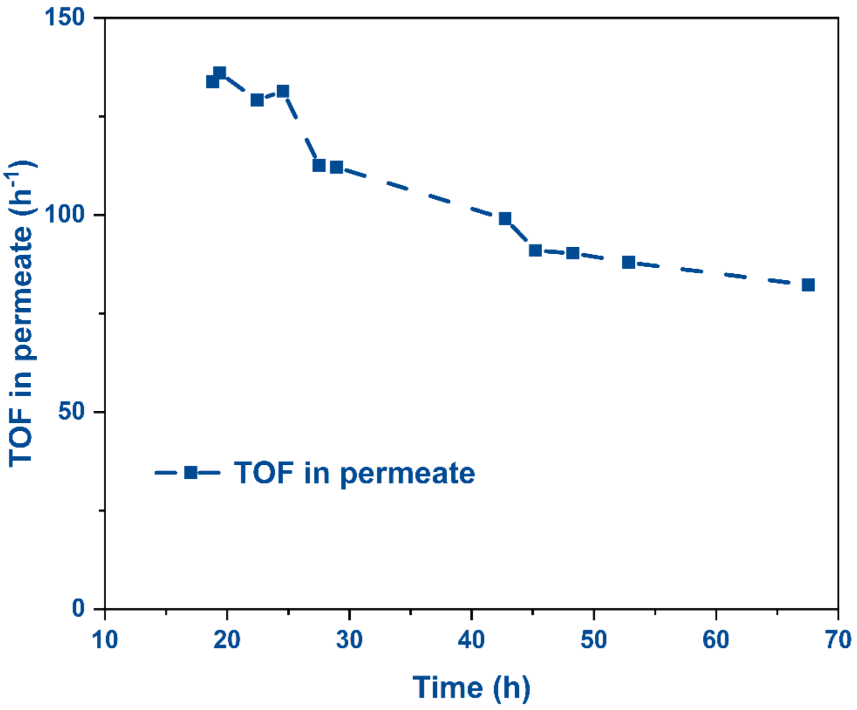

Supplementary Figure 29. TOF in permeate of the long-term run of photocatalytic C(sp^3)-H alkylation with inline TBADT recovery.

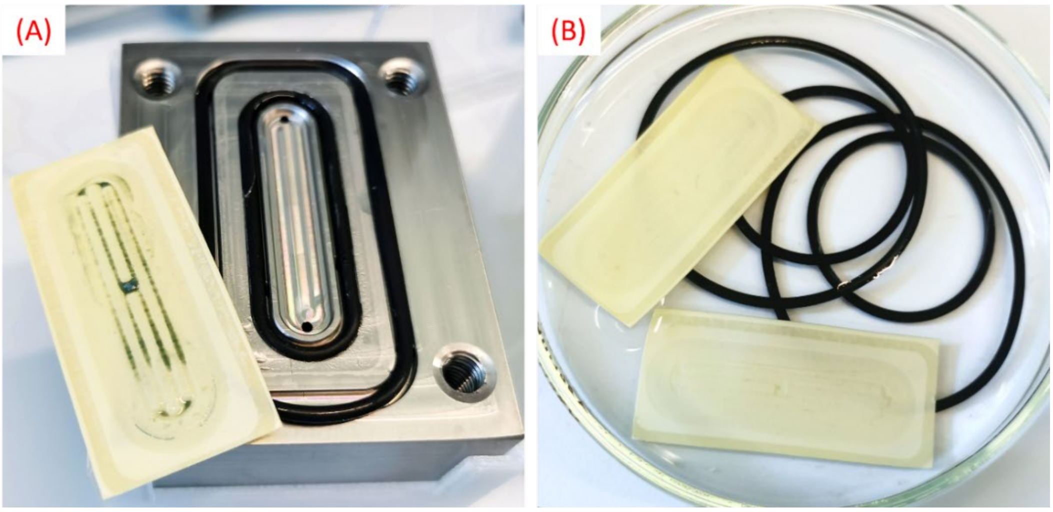

Supplementary Figure 30. Pictures of fouling membrane (A) and cleaned membrane (B) with clean CH_3CN.

6.Preliminary investigations of other photocatalysts recovery with Nanofiltration

To further highlight the potential of this technology, three commonly used photocatalysts were selected to see if it is practical to perform recovery with the nanofiltration process. The basic information about those photocatalysts is listed below (**Supplementary table 8**):

Supplementary table 8. Summary of the commercially available photocatalysts investigated with nanofiltration

Photocatalyst	CAS number	Price [a] (€/mmol)	Molecular weight (g/mol)
4CzIPN	1416881-52-1	4133.8	788.89
$Ru(bpy)_3(PF_6)_2$	60804-74-2	142.7	859.55
$(Ir[(dF(CF_3)ppy)]_2(dtbpy))PF_6$	870987-63-6	1537.0	1121.91

[a] The price data was calculated based on the Sigma-Aldrich website (16-08-2022)

Prior to starting the nanofiltration experiments, the UV spectra of each photocatalyst were recorded with an inline UV/Vis spectrometer. And a calibration curve for each photocatalyst was plotted versus the concentration of the photocatalyst. Besides, experimental samples were diluted to achieve concentrations that would fall in the linearity region of the photocatalyst calibration curve.

6.1 4CzIPN

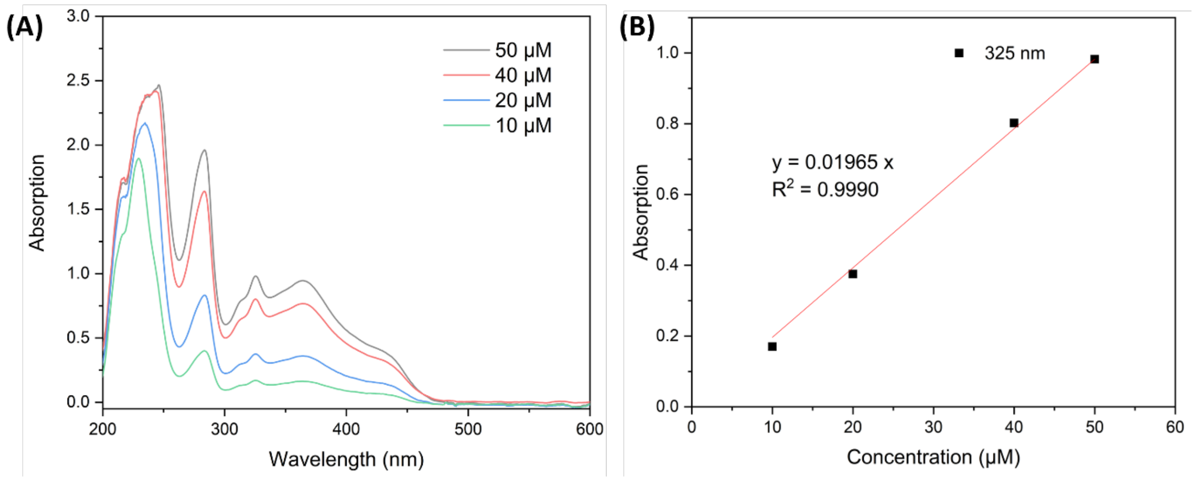

Supplementary Figure 31. Characterization of 4CzIPN with UV-Vis spectrometer. (A) Absorbance of 4CzIPN solution (in CH_3CN) with different concentrations. (B) Calibration curve of 4CzIPN concentration vs UV/Vis absorbance at 325 nm.

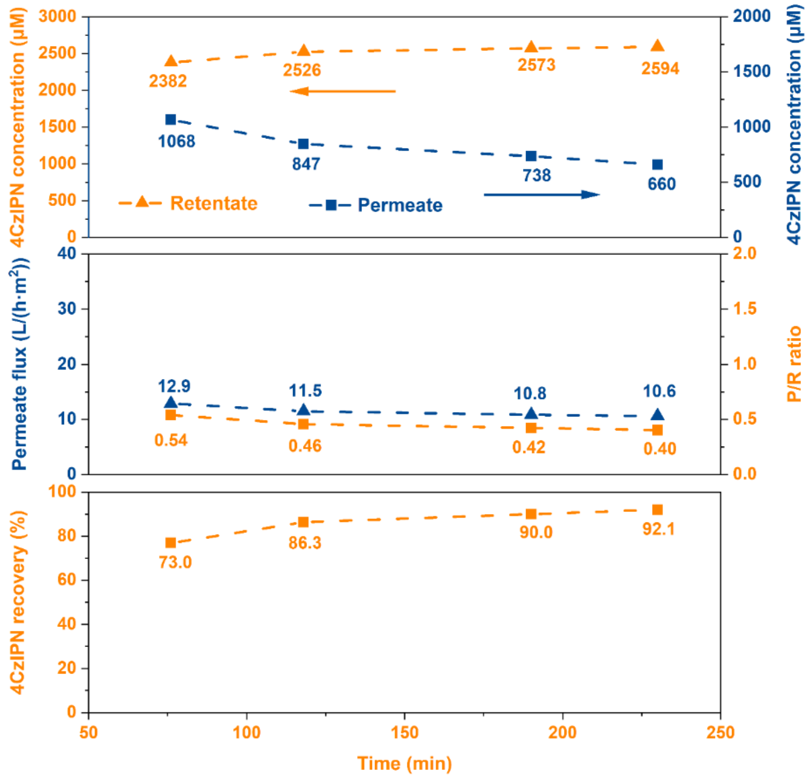

Supplementary Figure 32. Summary of 4CzIPN filtration experiment with Solsep NF030306 membrane. Experiment condition: 0.15 mL/min input flow rate, 35 bar, 2 mM 4CzIPN solution.

6.2 $Ru(bpy)_3(PF_6)_2$

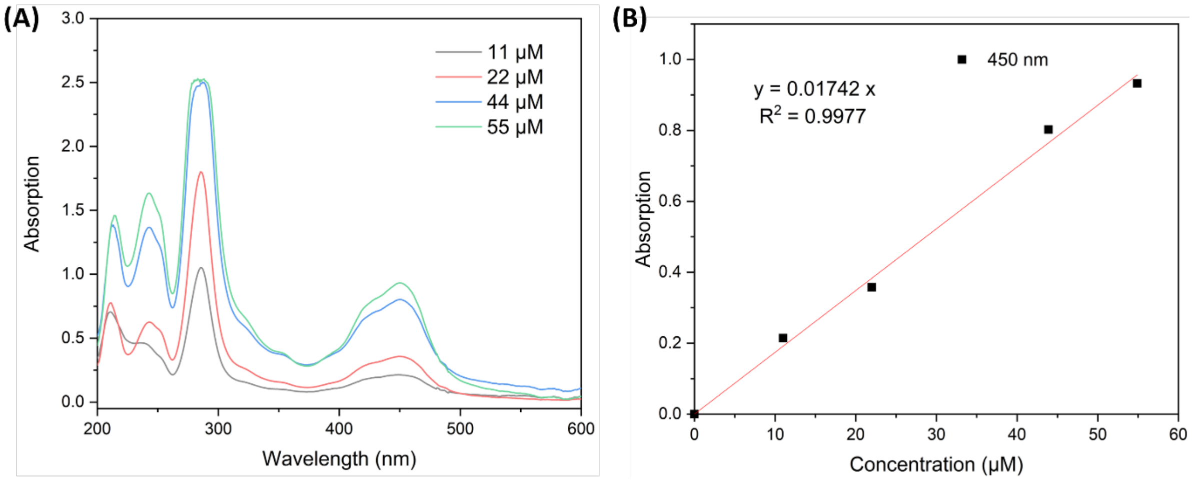

Supplementary Figure 33. Characterization of $Ru(bpy)_3(PF_6)_2$ with UV-Vis spectrometer. (A) Absorbance of $Ru(bpy)_3(PF_6)_2$ solution (in CH_3CN) with different concentrations. (B) Calibration curve of $Ru(bpy)_3(PF_6)_2$ concentration vs UV/Vis absorbance at 450 nm.

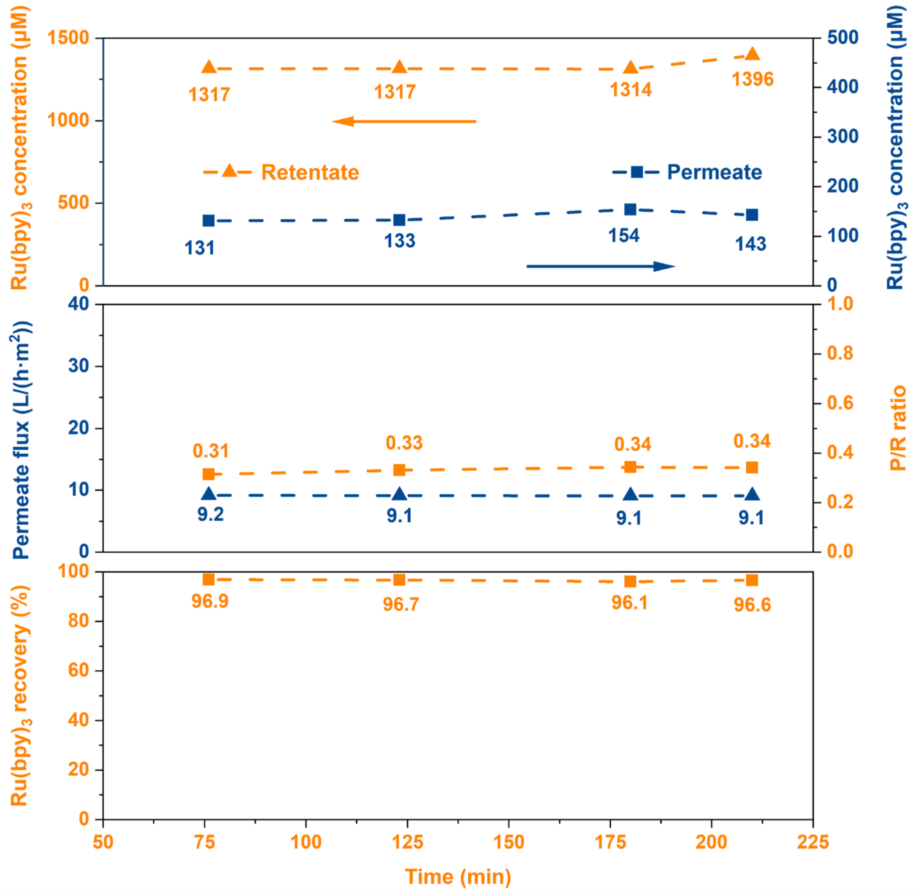

Supplementary Figure 34. Summary of $Ru(bpy)_3(PF_6)_2$ filtration experiment with Solsep NF030306 membrane. Experiment condition: 0.15 mL/min input flow rate, 35 bar, 1 mM $Ru(bpy)_3(PF_6)_2$ solution.

6.3 (Ir[(dF(CF_3)ppy)]$_2$(dtbpy))PF_6

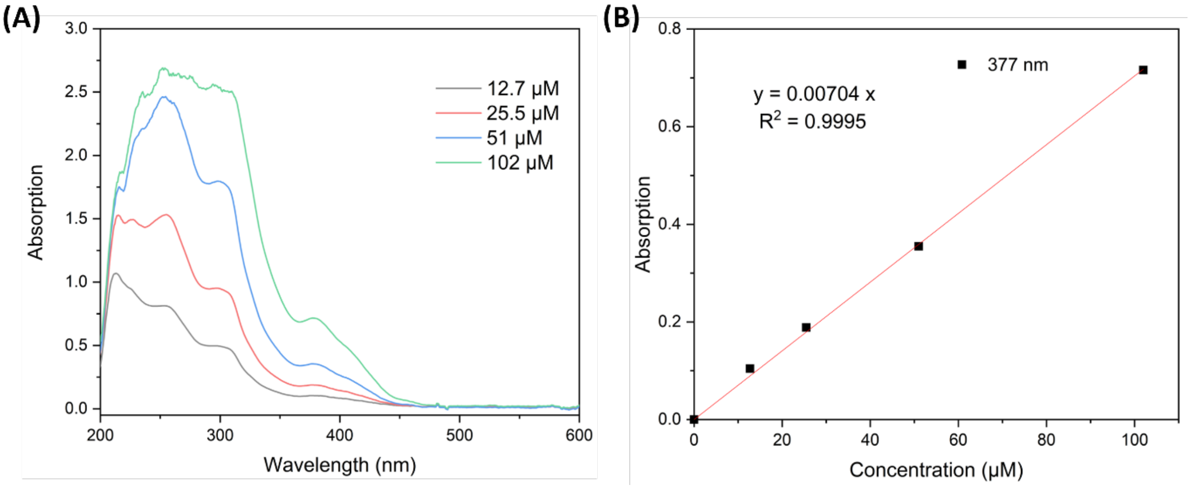

Supplementary Figure 35. Characterization of (Ir[(dF(CF_3)ppy)]$_2$(dtbpy))PF_6 with UV-Vis spectrometer. (A) Absorbance of (Ir[(dF(CF_3)ppy)]$_2$(dtbpy))PF_6 solution (in CH_3CN) with different concentrations. (B) Calibration curve of (Ir[(dF(CF_3)ppy)]$_2$(dtbpy))PF_6 concentration vs UV/Vis absorbance at 377 nm.

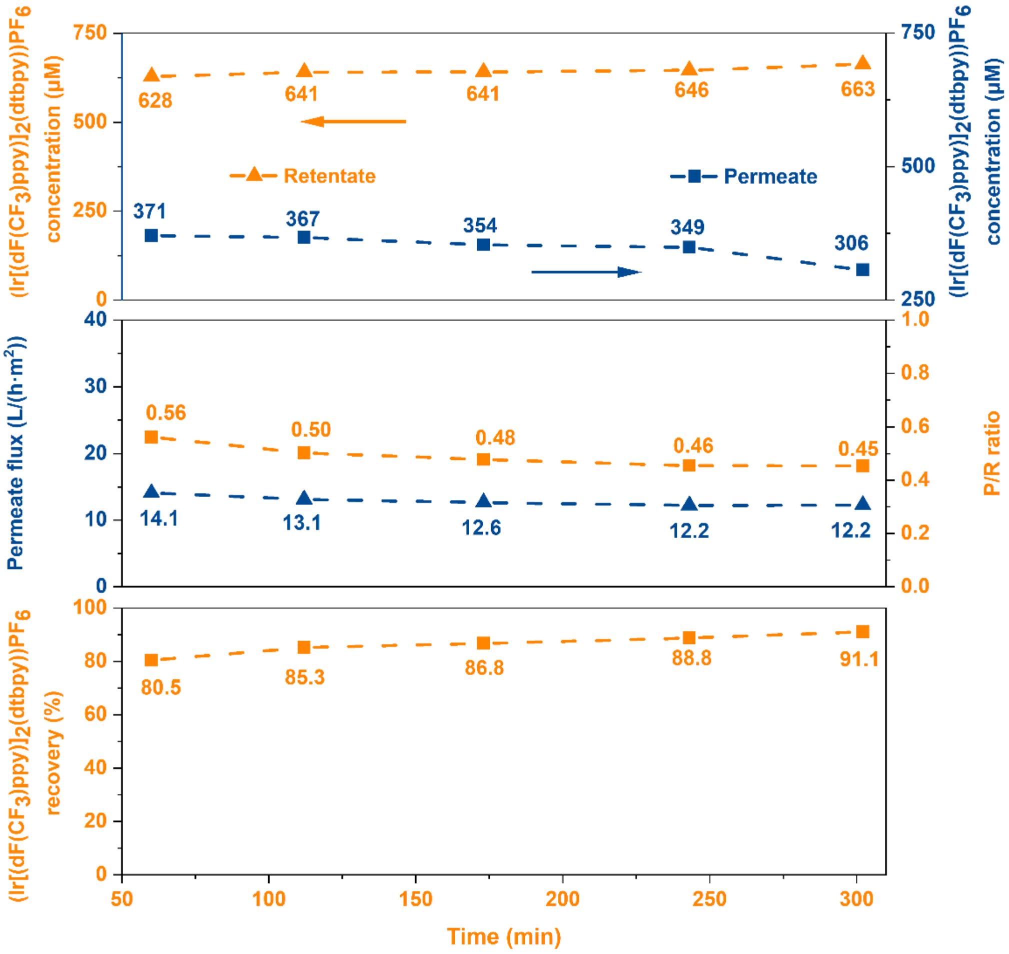

Supplementary Figure 36. Summary of (Ir[(dF(CF_3)ppy)]$_2$(dtbpy))PF_6 filtration experiment with Solsep NF030306 membrane. Experiment condition: 0.15 mL/min input flow rate, 35 bar, 0.5 mM (Ir[(dF(CF_3)ppy)]$_2$(dtbpy))PF_6 solution.

7. Characterization data

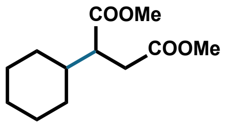

Dimethyl 2-cyclohexylsuccinate (1). The product was prepared following the general procedure **GP1** (20 mins residence time) and was purified by flash column chromatography on silica gel (cyclohexane: ethyl acetate 93:7) to afford the product as a colourless liquid (95.8 mg, 84 %).

The experiment with inline TBADT recovery was preformed following the general procedure **GP2** (20 mins residence time, 18.7 hours experimental time). A total TON of 2659 (5 µmol TBADT used, 13.30 mmol product formed) was obtained, including TON of 2359 from product in permeate solution and additional 300 TONs remaining in the system.

^{1}H NMR (300 MHz, $CDCl_3$) δ 3.68 (s, 3H), 3.65 (s, 3H), 2.78 - 2.65 (m, 2H), 2.44 (dt, J = 13.2, 8.9 Hz, 1H), 1.77 - 1.68 (m, 2H), 1.68 - 1.56 (m, 4H), 1.28 - 1.14 (m, 2H), 1.13 - 0.92 (m, 3H).

^{13}C NMR (75 MHz, $CDCl_3$) δ 175.1, 173.1, 51.9, 51.7, 47.1, 40.1, 33.4, 30.8, 30.3, 26.4, 26.3.

HRMS (FI) *m/z* calcd. for $C_{12}H_{20}O_4$ 228.1362, found: 228.1362.

The spectroscopic data are in agreement with the literature.[7]

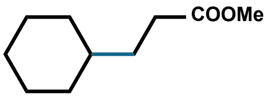

Methyl 3-cyclohexylpropanoate (2). The product was prepared following the general procedure **GP1** (20 mins residence time) and was purified by flash column chromatography on silica gel (cyclohexane: ethyl acetate 91:9) to afford the product as a colourless liquid (73.2 mg, 86 %).

The experiment with inline TBADT recovery was preformed following the general procedure **GP2** (20 mins residence time, 12.1 hours experimental time). A total TON of 1592 (5 µmol TBADT used, 7.96 mmol product formed) was obtained, including TON of 1357 from product in permeate solution and additional 235 TONs remaining in the system.

^{1}H NMR (300 MHz, $CDCl_3$) δ 3.66 (s, 3H), 2.36 - 2.26 (m, 2H), 1.79 - 1.60 (m, 5H), 1.60 - 1.46 (m, 2H), 1.30 - 1.11 (m, 4H), 0.96 - 0.80 (m, 2H).

^{13}C NMR (75 MHz, $CDCl_3$) δ 174.8, 51.6, 37.4, 33.1, 32.5, 31.8, 26.7, 26.4.

HRMS (EI) *m/z* calcd. for $C_{10}H_{18}O_2$ 170.1307, found: 170.1314.

The spectroscopic data are in agreement with the literature.[8]

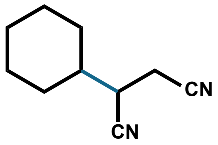

2-Cyclohexylsuccinonitrile (3). The product was prepared following the general procedure **GP1** (20 mins residence time) and was purified by flash column chromatography on silica gel (cyclohexane: ethyl acetate 80:20) to afford the product as a light yellow oil (57.5 mg, 71 %).

The experiment with inline TBADT recovery was preformed following the general procedure **GP2** (20 mins residence time, 13 hours experimental time). A total TON of 1177 (5 µmol TBADT used, 5.89 mmol product formed) was obtained, including TON of 865 from product in permeate solution and additional 312 TONs remaining in the system.

^{1}H NMR (300 MHz, $CDCl_3$) δ 2.86 - 2.64 (m, 3H), 1.96 - 1.65 (m, 5H), 1.39 - 1.10 (m, 6H). **^{13}C NMR** (75 MHz, $CDCl_3$) δ 118.3, 116.0, 38.5, 34.8, 31.0, 28.9, 25.7, 25.6, 25.5, 18.8.

HRMS (EI) *m/z* calcd. for $C_{10}H_{14}N_2$ 162.1157, found: 162.1144.

The spectroscopic data are in agreement with the literature.[9]

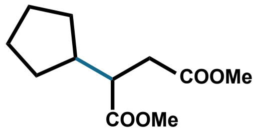

Dimethyl 2-cyclopentylsuccinate (4). The product was prepared following the general procedure **GP1** (20 mins residence time) and was purified by flash column chromatography on silica gel (cyclohexane: ethyl acetate 93:7) to afford the product as a colourless liquid (73.9 mg, 69 %).

The experiment with inline TBADT recovery was preformed following the general procedure **GP2** (20 mins residence time, 10.7 hours experimental time). A total TON of 1004 (5 µmol TBADT used, 5.02 mmol product formed) was obtained, including TON of 821 from product in permeate solution and additional 183 TONs remaining in the system.

^{1}H NMR (300 MHz, $CDCl_3$) δ 3.66 (s, 3H), 3.62 (s, 3H), 2.78 - 2.67 (m, 1H), 2.67 - 2.59 (m, 1H), 2.54 - 2.41 (m, 1H), 2.01 - 1.85 (m, 1H), 1.81 - 1.71 (m, 1H), 1.70 - 1.55 (m, 3H), 1.55 - 1.44 (m, 2H), 1.32 - 1.21 (m, 1H), 1.21 - 1.07 (m, 1H).

^{13}C NMR (75 MHz, $CDCl_3$) δ 175.4, 172.7, 51.8, 51.7, 46.3, 42.5, 35.5, 30.6, 30.5, 25.0, 24.9.

HRMS (FI) *m/z* calcd. for $C_{11}H_{18}O_4$ 214.1205, found: 214.1184.

The spectroscopic data are in agreement with the literature.[7]

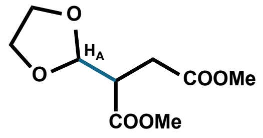

5, H_A

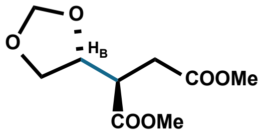

5', H_B-*anti*

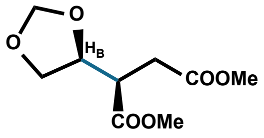

5', H_B-*syn*

Dimethyl 2-(1,3-dioxolan-2-yl)succinate (5, H_A), dimethyl (*R*)-2-((*R*)-1,3-dioxolan-4-yl)succinate (5', H_B-*anti*) and dimethyl (*R*)-2-((*S*)-1,3-dioxolan-4-yl)succinate (5', H_B-*syn*). The products were prepared following the general procedure **GP1** (20 mis residence time, 1.2 mol% TBADT) and were purified by flash column chromatography on silica gel (cyclohexane: ethyl acetate 85:15) to afford the products as colourless liquids (87.2 mg, 80 %). The ratio of different products is calculated by GC-MS analysis of the crude reaction mixture.

The experiment with inline TBADT recovery was preformed following the general procedure **GP2** (20 mins residence time, 1.2 mol% TBADT, 14 hours experimental time). A total TON of 1201 (6 μmol TBADT used, 7.21 mmol product formed) was obtained, including TON of 831 from product in permeate solution and additional 369 TONs remaining in the system.

5, H_A: ^{1}**H NMR** (300 MHz, $CDCl_3$) δ 5.19 (d, *J* = 4.2 Hz, 1H), 4.01 – 3.93 (m, 2H), 3.93 – 3.85 (m, 2H), 3.74 (s, 3H), 3.68 (s, 3H), 3.25 (ddd, *J* = 9.1, 4.9, 4.1 Hz, 1H), 2.80 (dd, *J* = 16.9, 9.2 Hz, 1H), 2.62 (dd, *J* = 16.9, 4.9 Hz, 1H).

^{13}**C NMR** (75 MHz, $CDCl_3$) δ 172.5, 171.6, 102.9, 65.5, 52.5, 52.0, 45.8, 30.2.

HRMS (EI) *m/z* calcd. for $C_9H_{14}O_6$ 218.0790, found: 218.0724.

5', H_B-*anti*: ^{1}**H NMR** (300 MHz, $CDCl_3$) δ 4.99 (s, 1H), 4.82 (s, 1H), 4.24 (ddd, *J* = 8.2, 6.6, 5.4 Hz, 1H), 4.02 (dd, *J* = 8.7, 6.5 Hz, 1H), 3.78 (dd, *J* = 8.7, 5.4 Hz, 1H), 3.72 (s, 3H), 3.69 (s, 3H), 2.98 (td, *J* = 7.9, 5.0 Hz, 1H), 2.90 - 2.80 (m, 1H), 2.75 (dd, *J* = 16.9, 5.1 Hz, 1H).

^{13}**C NMR** (75 MHz, $CDCl_3$) δ 172.5, 172.4, 95.3, 74.9, 69.1, 52.4, 52.1, 45.1, 32.6.

HRMS (FI) *m/z* calcd. for $C_9H_{14}O_6$ 218.0790, found: 218.0758.

5', H_B-*syn*: ^{1}**H NMR** (300 MHz, $CDCl_3$) δ 5.05 (s, 1H), 4.80 (s, 1H), 4.29 (dt, *J* = 6.7, 5.6 Hz, 1H), 3.88 - 3.84 (m, 1H), 3.81 (dd, *J* = 8.6, 5.5 Hz, 1H), 3.73 (s, 3H), 3.68 (s, 3H), 3.19 (ddd, *J* = 9.7, 4.9, 4.0 Hz, 1H), 2.80 (dd, *J* = 16.6, 9.6 Hz, 1H), 2.51 (dd, *J* = 16.8, 4.8 Hz, 1H).

^{13}**C NMR** (75 MHz, $CDCl_3$) δ 172.5, 172.1, 95.7, 75.2, 67.2, 52.4, 52.1, 44.4, 32.0.

HRMS (FI) *m/z* calcd. for $C_9H_{14}O_6$ 218.0790, found: 218.0748.

The spectroscopic data are in agreement with the literature.[10]

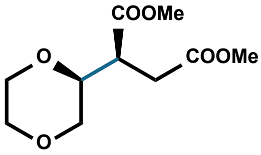

6-*syn*

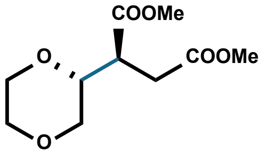

6'-*anti*

dimethyl (*R*)-2-((*S*)-1,4-dioxan-2-yl)succinate (6-*syn*) and dimethyl (*R*)-2-((*R*)-1,4-dioxan-2-yl)succinate (6'-*anti*). The products were prepared following the general procedure **GP1** (20 mins residence time, 1.2 mol% TBADT) and were purified by flash column chromatography on silica gel (cyclohexane: ethyl acetate 80:20) to afford the products as colourless liquids (99.9 mg, 86 %). The ratio of different products is calculated by GC-MS analysis of the crude reaction mixture.

The experiment with inline TBADT recovery was preformed following the general procedure **GP2** (20 mins residence time, 1.2 mol% TBADT, 15.8 hours experimental time). A total TON of 2148 (6 µmol TBADT used, 12.89 mmol product formed) was obtained, including TON of 1716 from product in permeate solution and additional 432 TONs remaining in the system.

6-*syn*: ^{1}H NMR (300 MHz, $CDCl_3$) δ 3.77 - 3.57 (m, 11H), 3.49 (td, *J* = 11.3, 2.9 Hz, 1H), 3.39 (dd, *J* = 11.4, 10.0 Hz, 1H), 2.95 (ddd, *J* = 9.9, 5.4, 4.6 Hz, 1H), 2.76 (dd, *J* = 16.9, 10.0 Hz, 1H), 2.46 (dd, *J* = 16.9, 4.6 Hz, 1H).

^{13}C NMR (75 MHz, $CDCl_3$) δ 172.4, 172.1, 75.4, 68.8, 67.3, 66.4, 52.3, 52.0, 43.6, 32.4.

HRMS (EI) *m/z* calcd. for $C_{10}H_{16}O_6$ 232.0947, found: 232.0949.

6'-*anti*: ^{1}H NMR (300 MHz, $CDCl_3$) δ 3.81 - 3.62 (m, 11H), 3.60 - 3.49 (m, 1H), 3.37 (dd, *J* = 11.9, 10.2 Hz, 1H), 2.91 (ddd, *J* = 8.8, 7.1, 4.9 Hz, 1H), 2.84 - 2.63 (m, 2H).

^{13}C NMR (75 MHz, $CDCl_3$) δ 172.7, 172.6, 74.7, 69.6, 67.2, 66.5, 52.4, 52.0, 43.7, 32.0.

HRMS (EI) *m/z* calcd. for $C_{10}H_{16}O_6$ 232.0947, found: 232.0951.

The spectroscopic data are in agreement with the literature.[10]

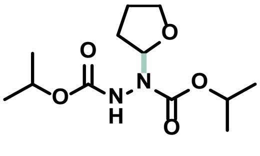

diisopropyl (*R*)-1-(tetrahydrofuran-2-yl)hydrazine-1,2-dicarboxylate (7)

The product was prepared following the general procedure **GP2** (1 mmol scale, 20 mins residence time, 0.4 mol% TBADT) and was purified by flash column chromatography on silica

gel (cyclohexane: ethyl acetate 80:20) to afford the product as colourless liquid (222.1 mg, 81 %).

The experiment with inline TBADT recovery was preformed following the general procedure **GP2** (20 mins residence time, 0.4 mol% TBADT, 27.1 hours experimental time). A total TON of 8423 (4 µmol TBADT used, 35.47 mmol product formed) was obtained, including TON of 8018 from product in permeate solution and additional 405 TONs remaining in the system.

^{1}H NMR (300 MHz, $CDCl_3$) δ 6.52 – 6.26 (m, 1H), 5.96 (brs, 1H), 5.03 – 4.79 (m, 2H), 3.95 (q, *J* = 6.7 Hz, 1H), 3.72 (q, *J* = 7.2 Hz, 1H), 2.14 – 1.73 (m, 4H), 1.23 – 1.17 (m, 12H).

^{13}C NMR (75 MHz, $CDCl_3$) δ 156.5, 155.1, 87.4, 70.6, 70.0, 69.8, 68.7, 28.3, 25.3, 22.1, 22.0.

HRMS (ESI) *m/z* calcd. for $C_{12}H_{22}N_2O_5$ 297.1426, found: 297.1423.

The spectroscopic data are in agreement with the literature.[11]

8. NMR spectra

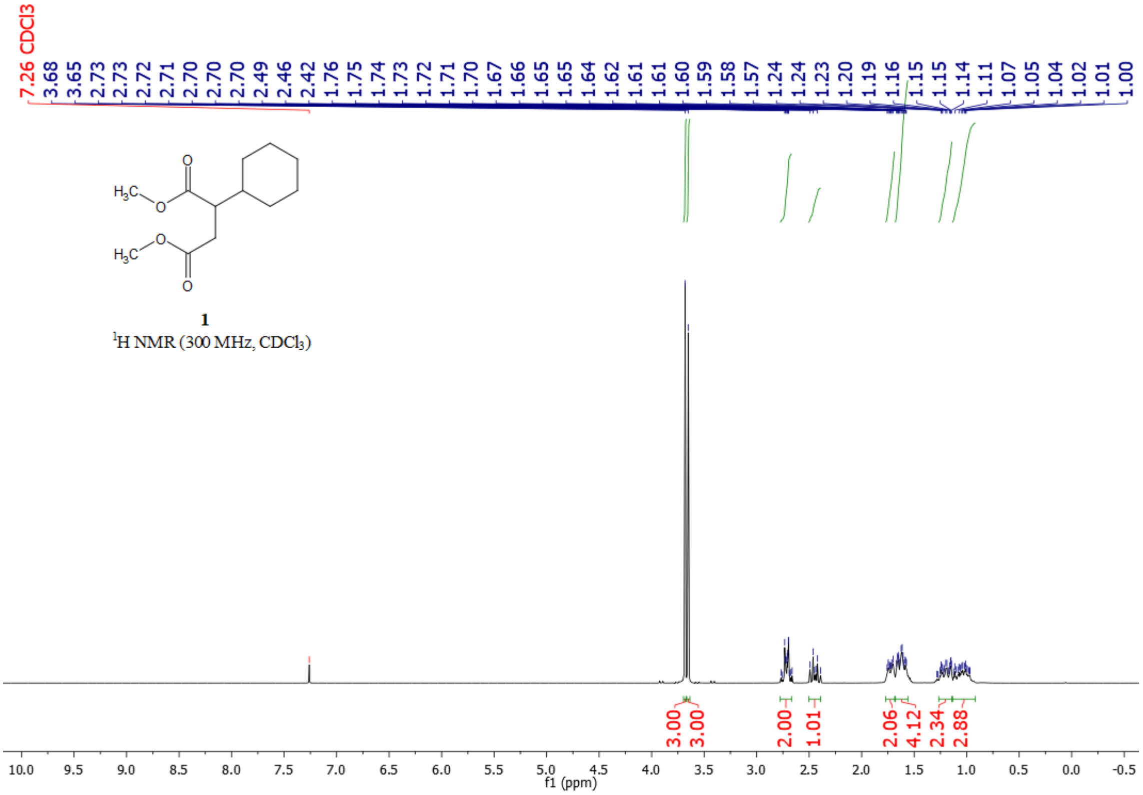

Supplementary Figure 37. ^{1}H NMR (300 MHz, $CDCl_3$) spectrum of compound **1**.

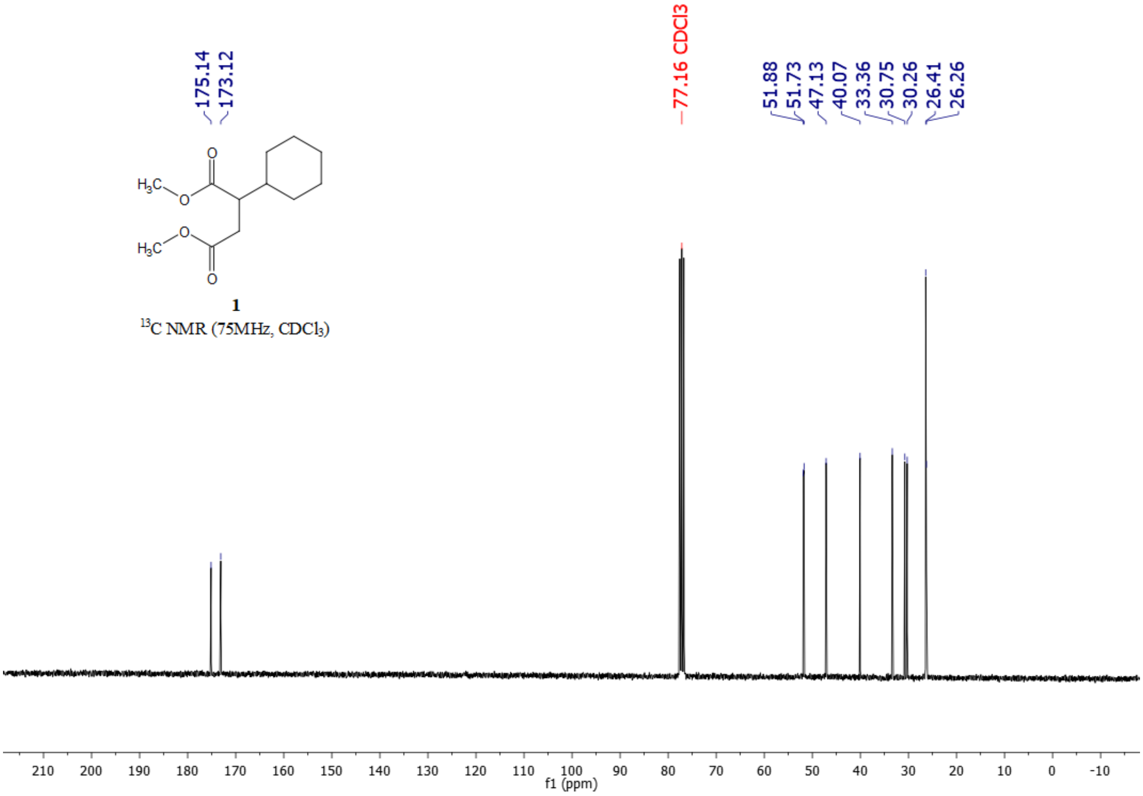

Supplementary Figure 38. ^{13}C NMR (75 MHz, $CDCl_3$) spectrum of compound **1**.

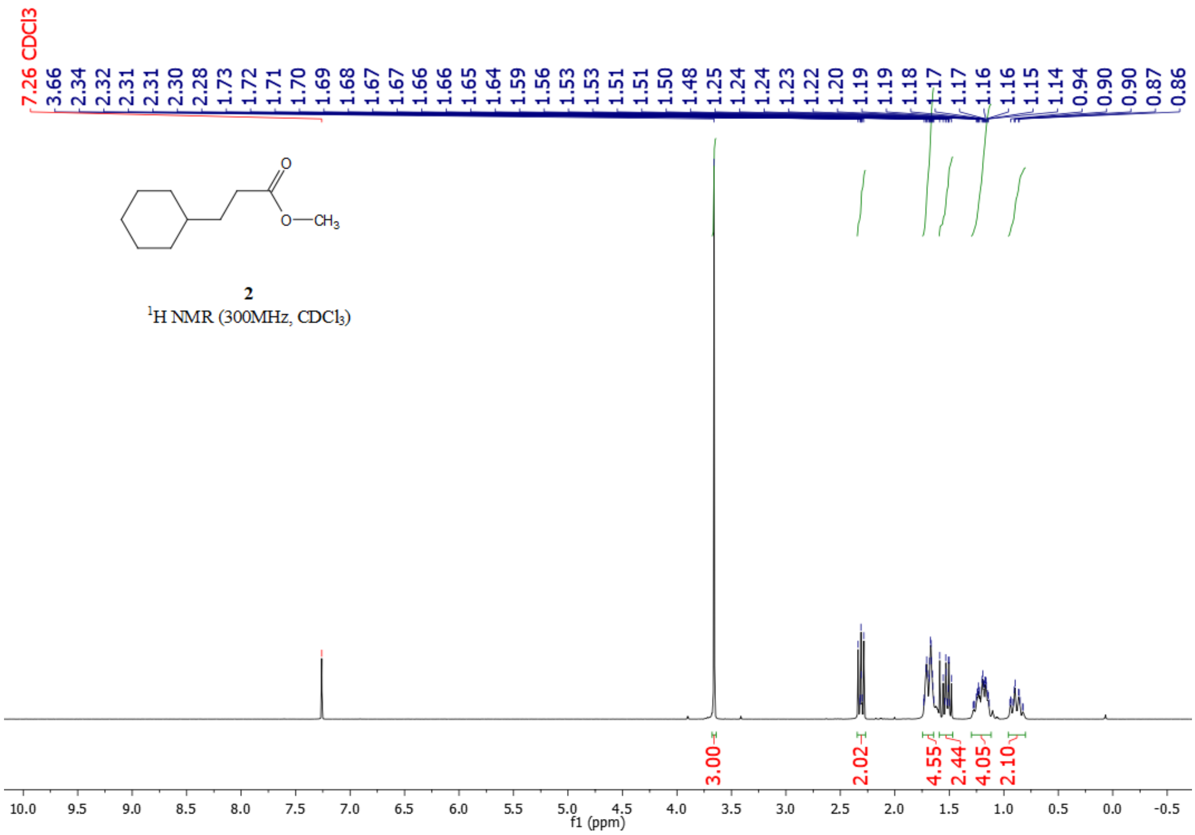

Supplementary Figure 39. ^{1}H NMR (300 MHz, $CDCl_3$) spectrum of compound **2**.

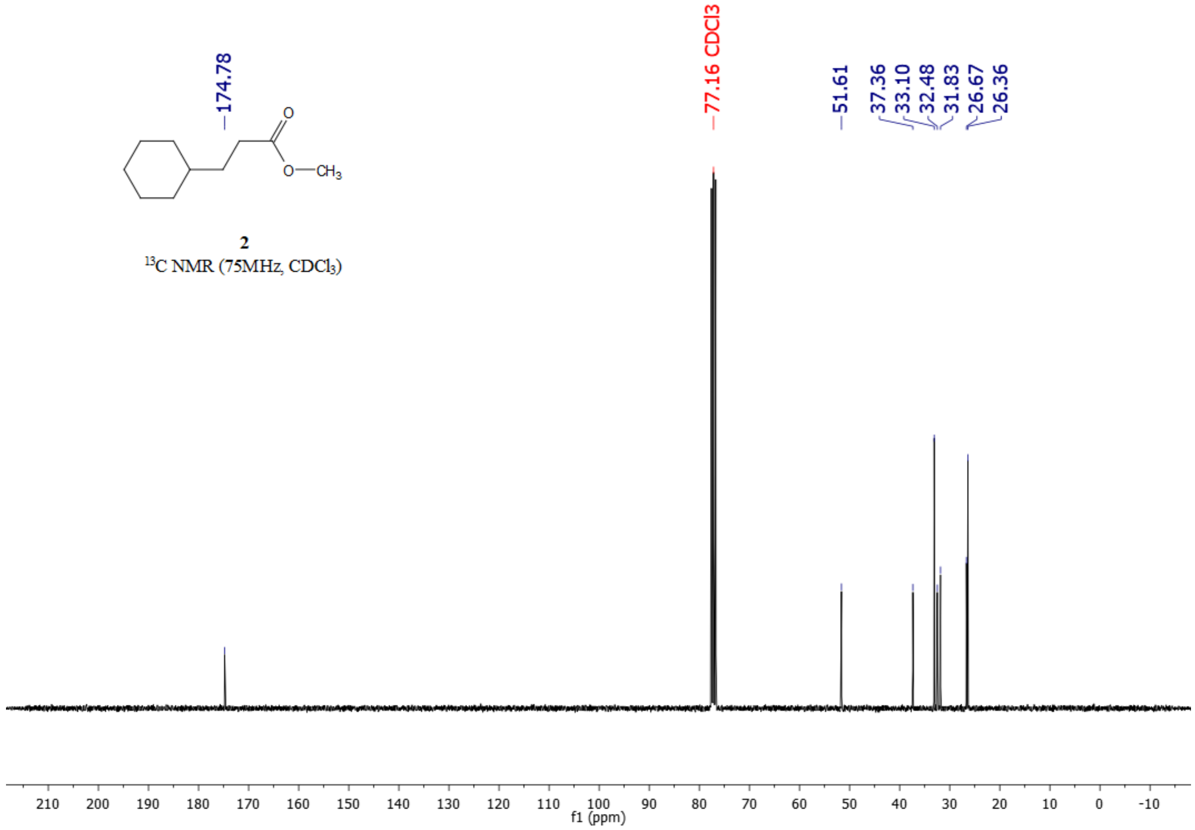

Supplementary Figure 40. ^{13}C NMR (75 MHz, $CDCl_3$) spectrum of compound **2**.

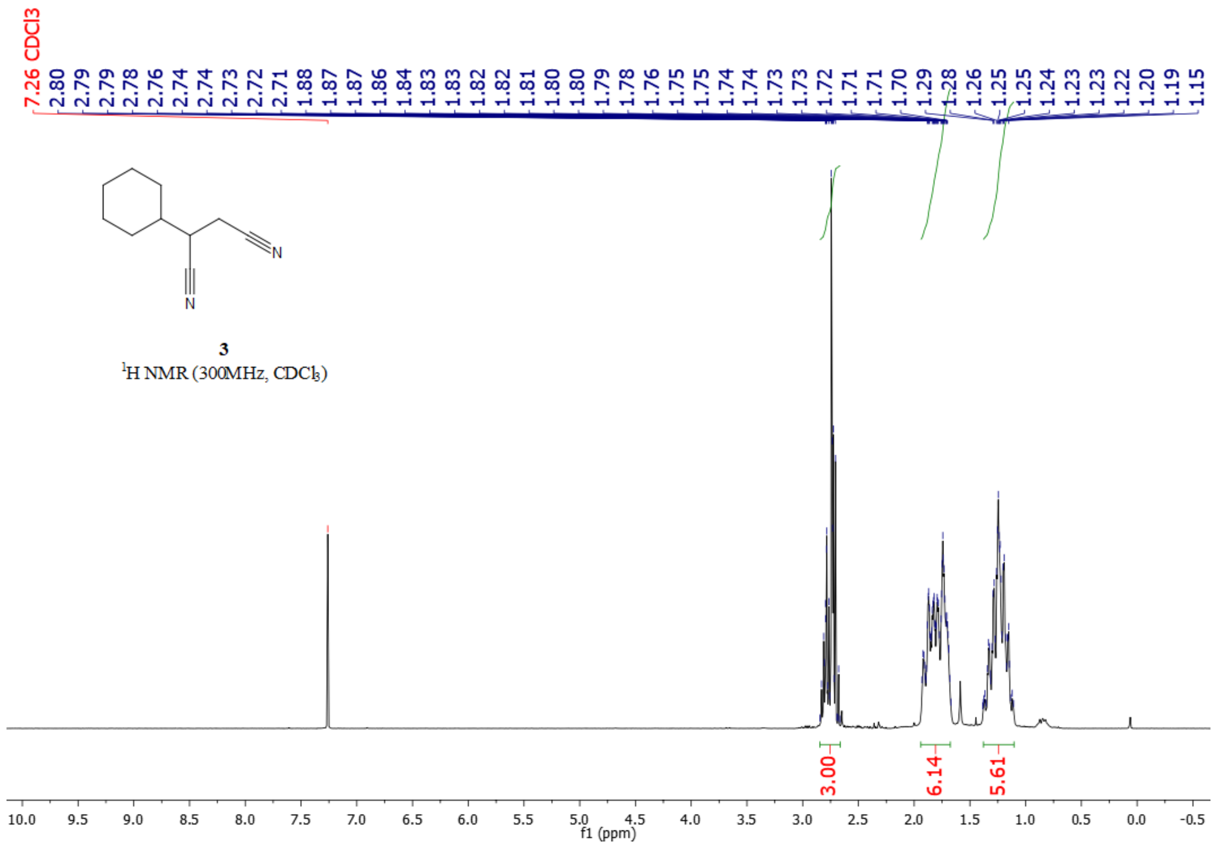

Supplementary Figure 41. ^{1}H NMR (300 MHz, $CDCl_3$) spectrum of compound **3**.

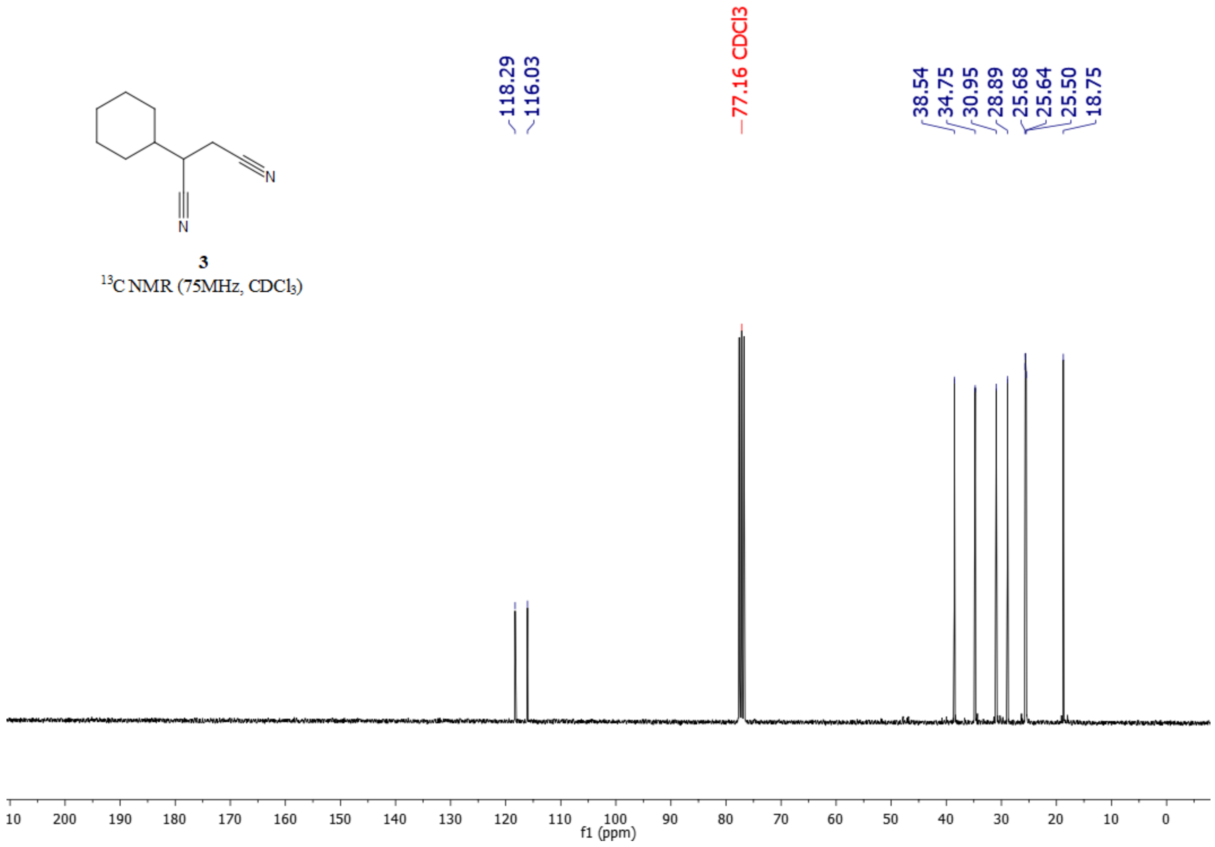

Supplementary Figure 42. ^{13}C NMR (75 MHz, $CDCl_3$) spectrum of compound **3**.

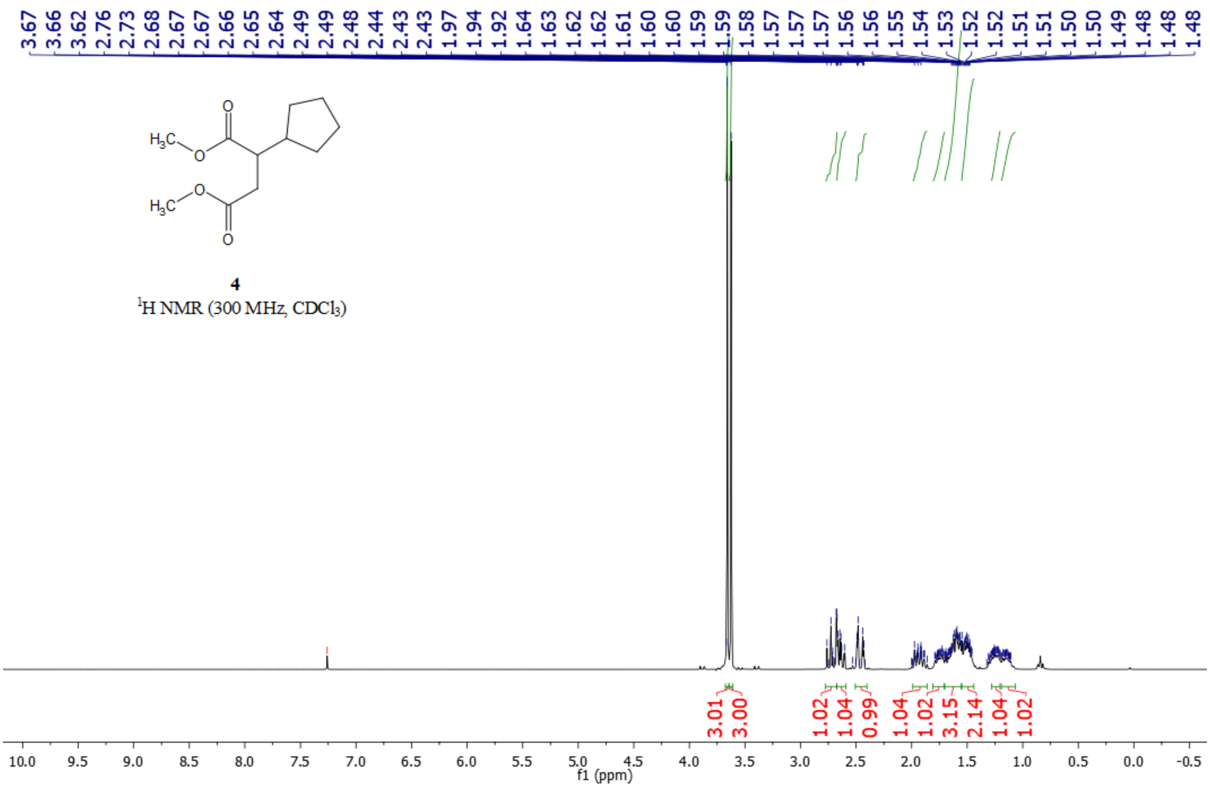

Supplementary Figure 43. ^{1}H NMR (300 MHz, $CDCl_3$) spectrum of compound **4**.

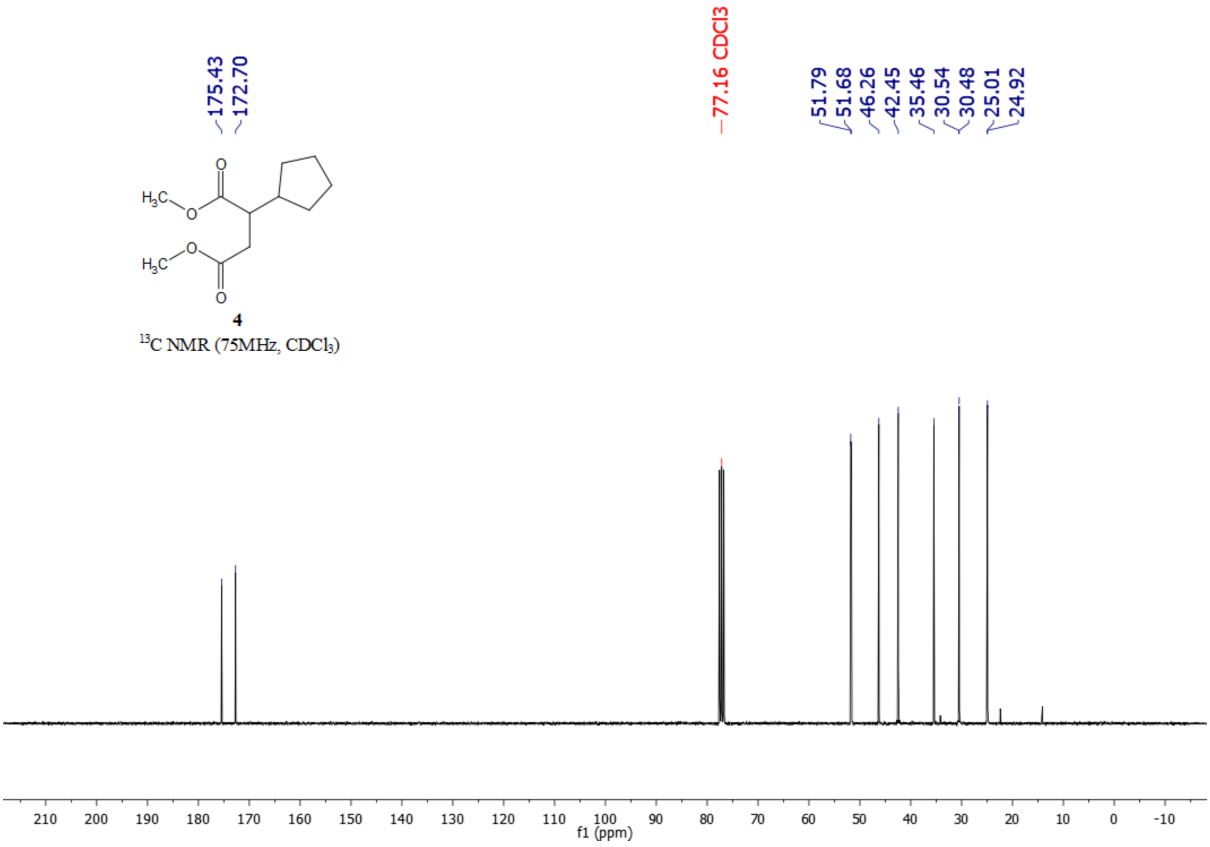

Supplementary Figure 44. ^{13}C NMR (75 MHz, $CDCl_3$) spectrum of compound **4**.

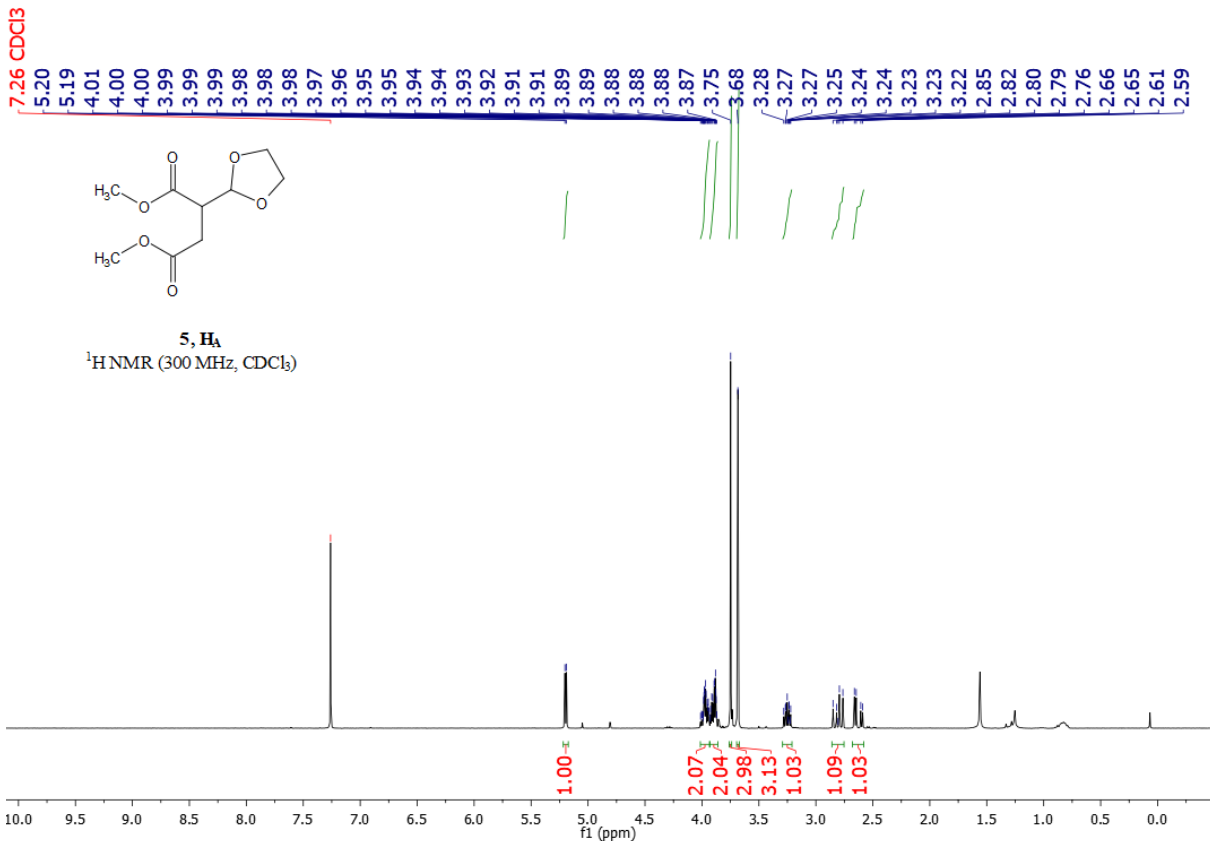

Supplementary Figure 45. ^{1}H NMR (300 MHz, $CDCl_3$) spectrum of compound **5-H$_A$**.

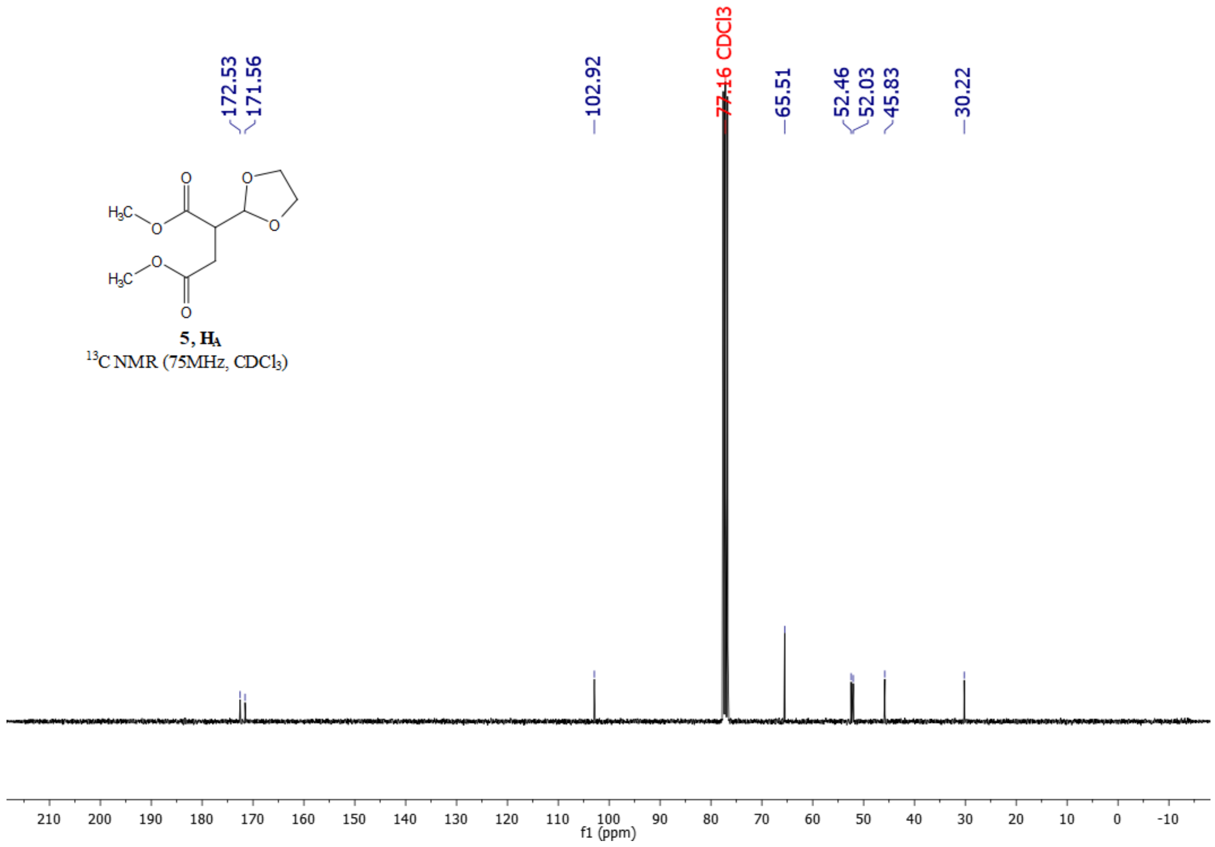

Supplementary Figure 46. ^{13}C NMR (75 MHz, $CDCl_3$) spectrum of compound **5-H$_A$**.

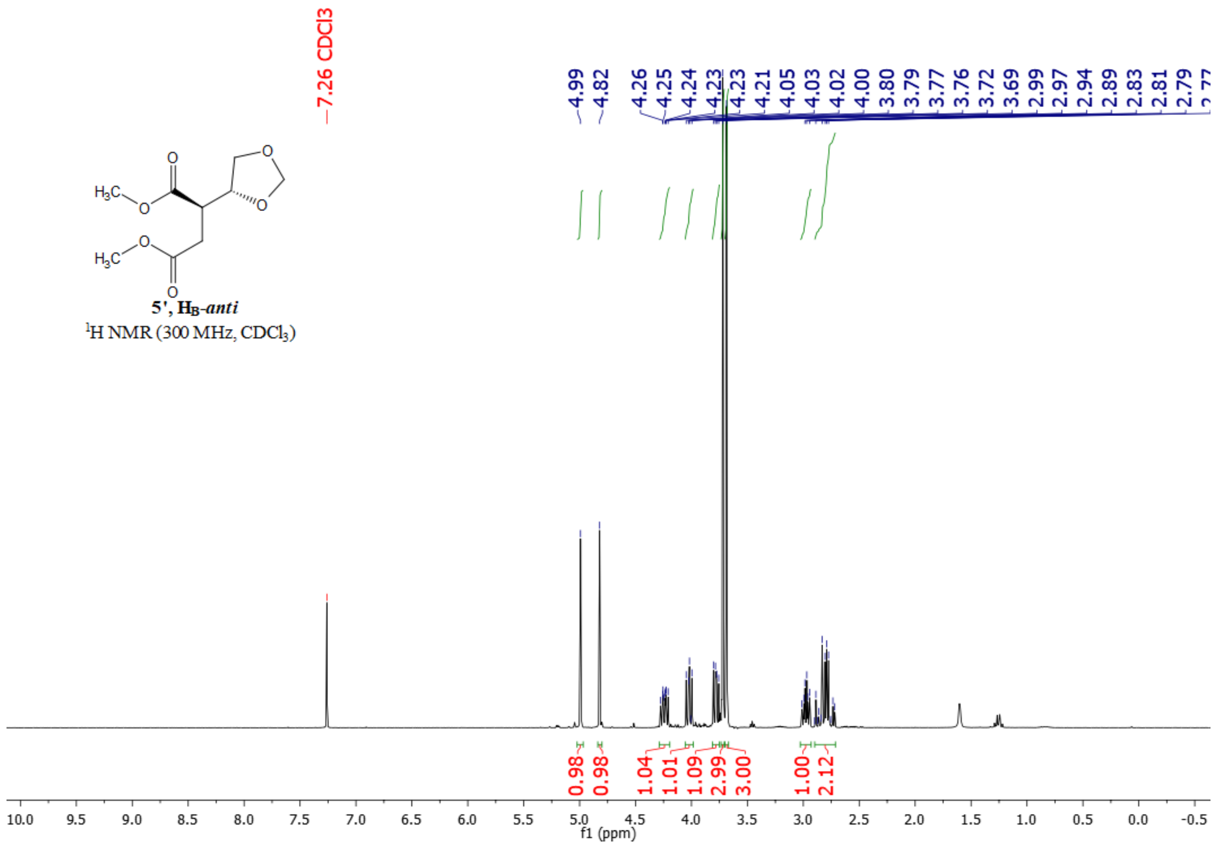

Supplementary Figure 47. ^{1}H NMR (300 MHz, $CDCl_3$) spectrum of compound **5'-H$_B$-*anti***.

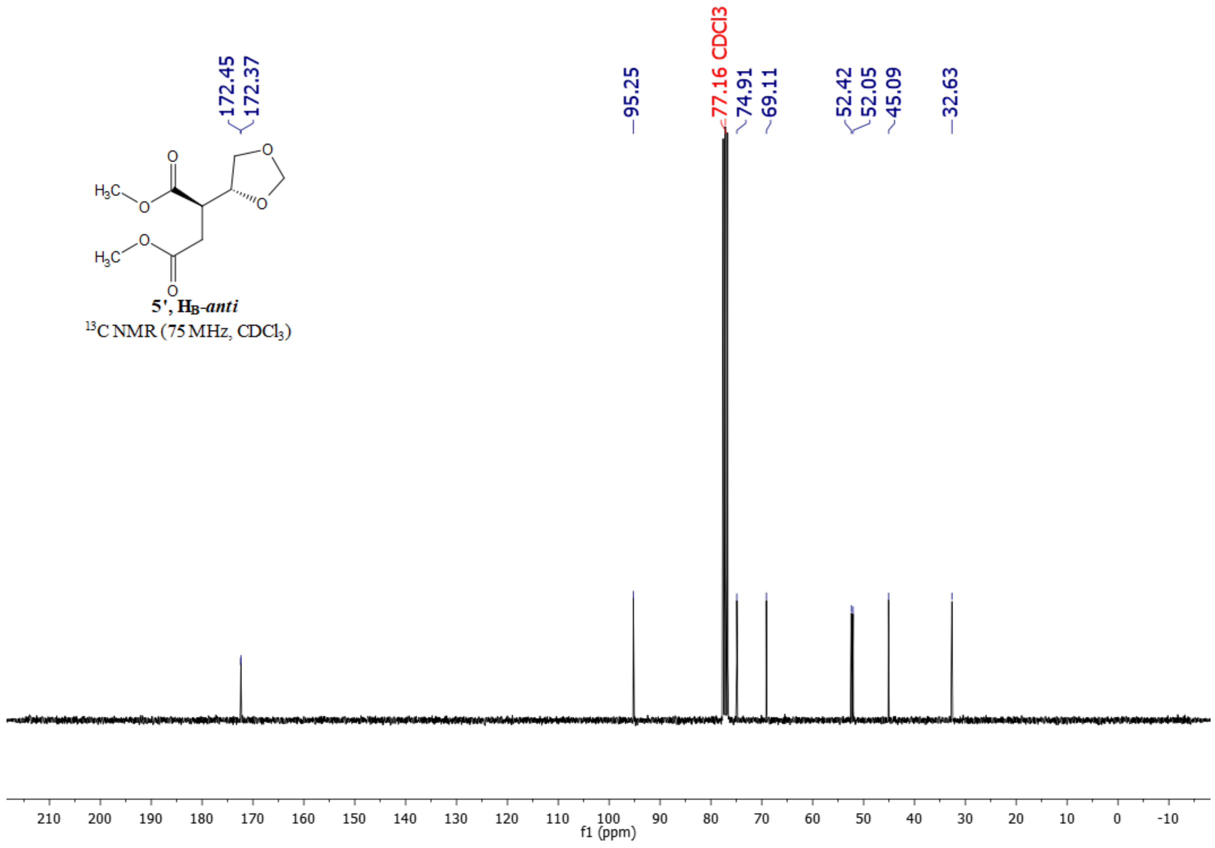

Supplementary Figure 48. ^{13}C NMR (75 MHz, $CDCl_3$) spectrum of compound **5'-H$_B$-*anti***.

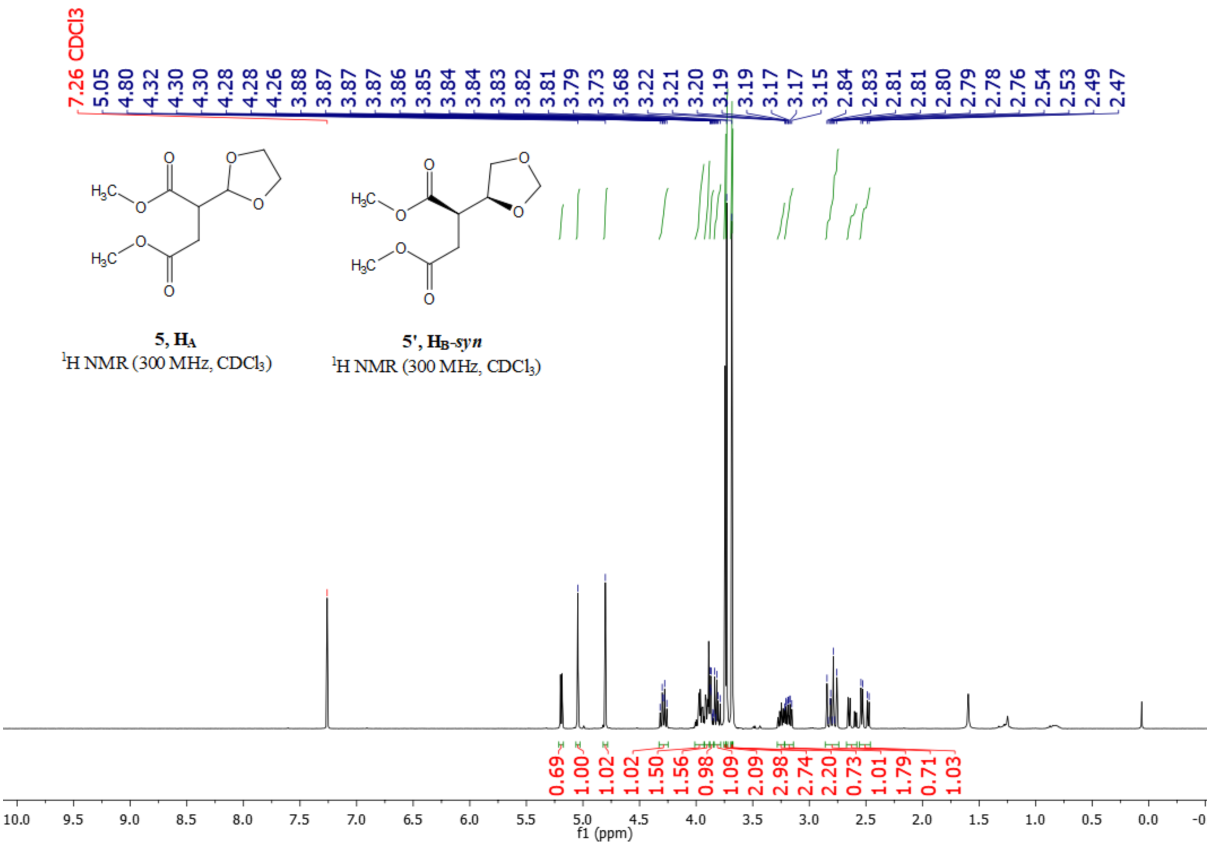

Supplementary Figure 49. ^{1}H NMR (300 MHz, CDCl$_3$) spectrum of **Mixture of 5-H$_A$ and 5'-H$_B$-*syn***.

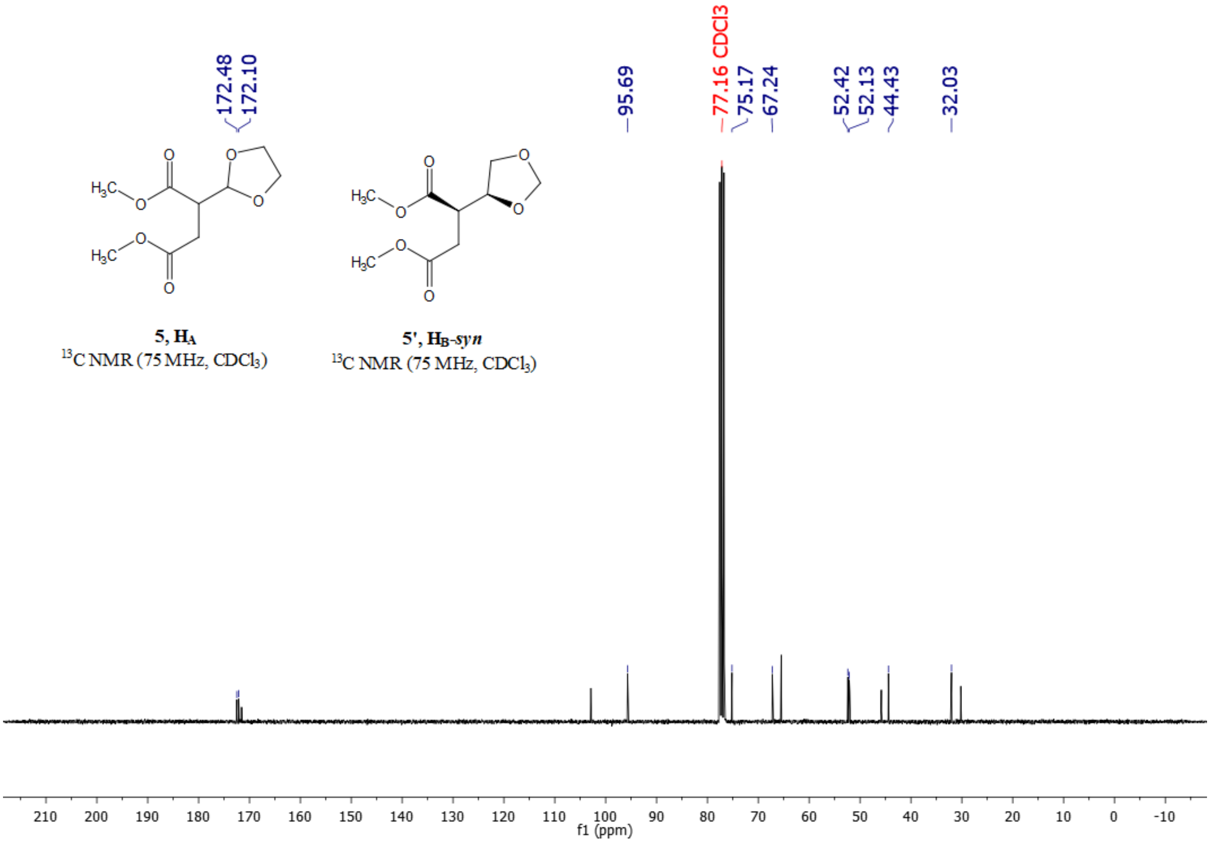

Supplementary Figure 50. ^{13}C NMR (75 MHz, CDCl$_3$) spectrum of **Mixture of 5-H$_A$ and 5'-H$_B$-*syn***.

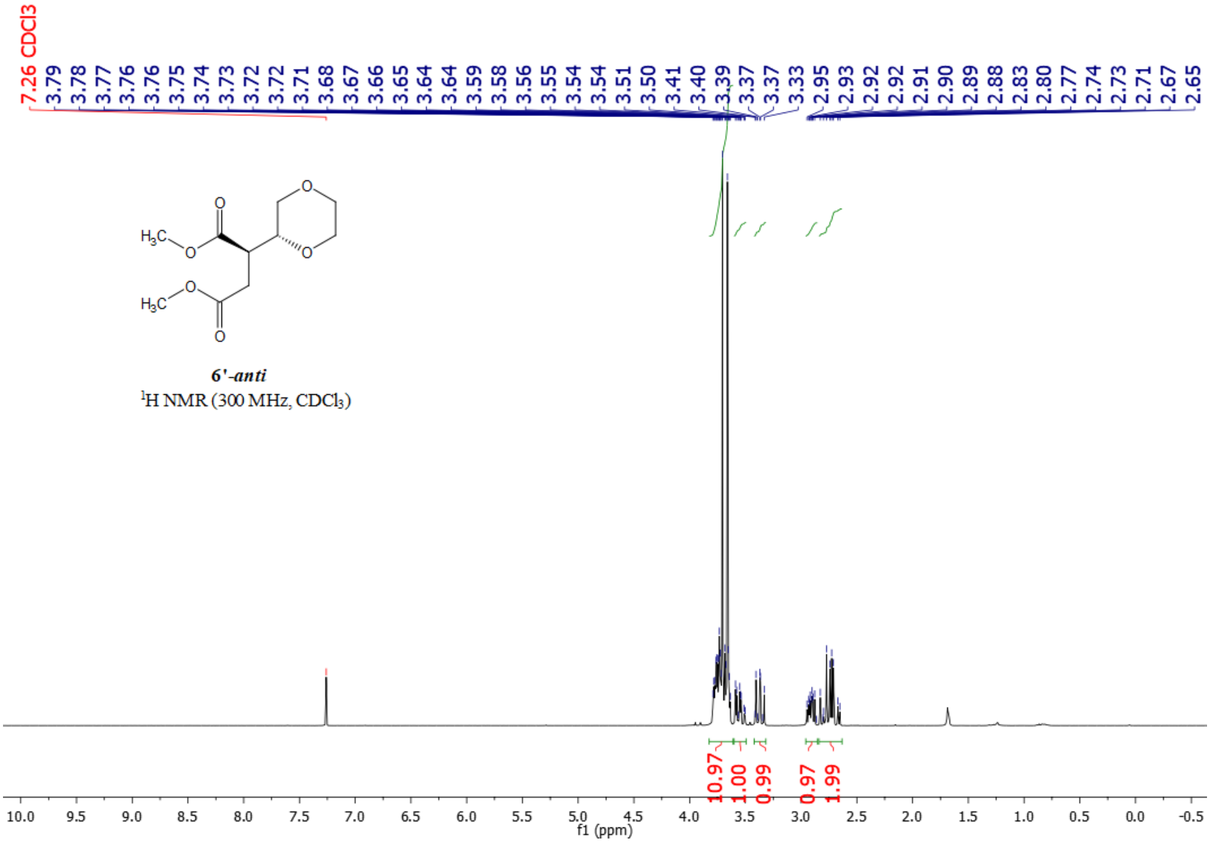

Supplementary Figure 51. ^{1}H NMR (300 MHz, $CDCl_3$) spectrum of compound **6-*anti***.

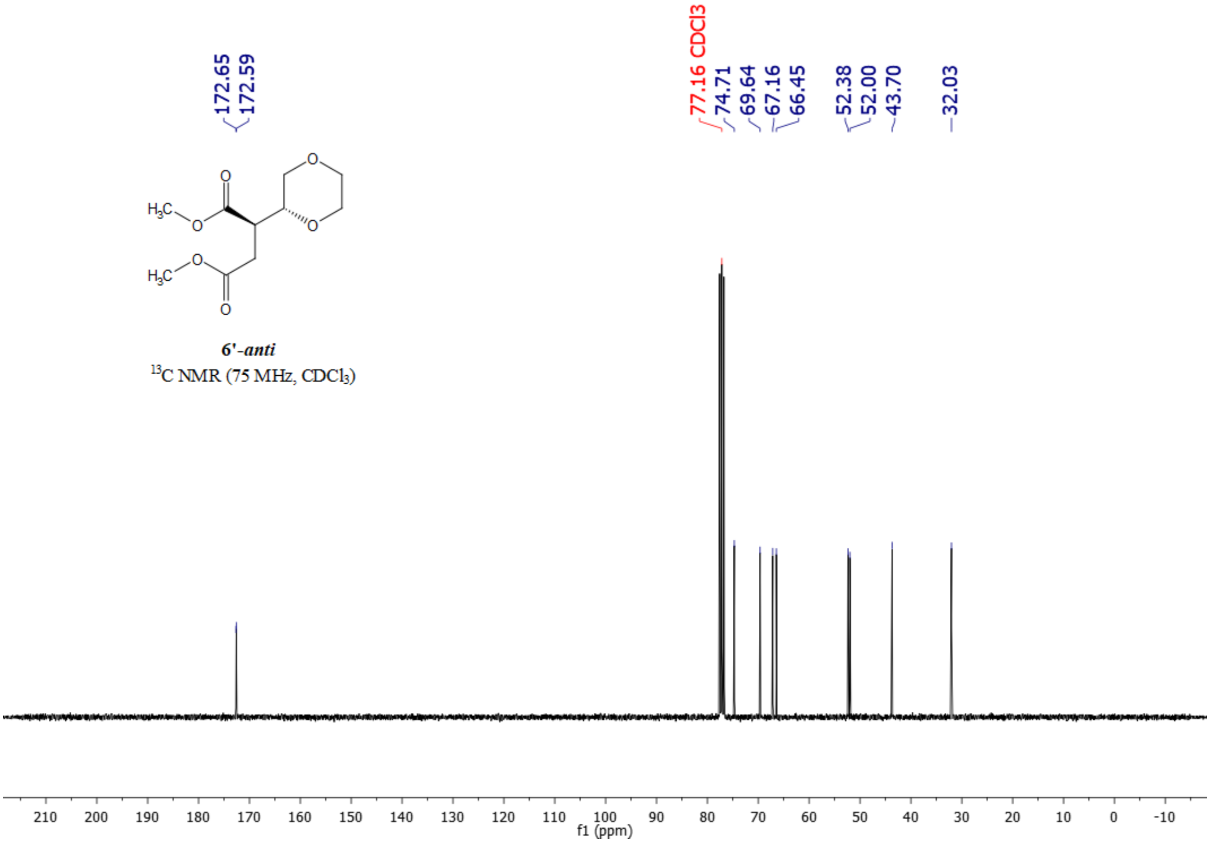

Supplementary Figure 52. ^{13}C NMR (75 MHz, $CDCl_3$) spectrum of compound **6-*anti***.

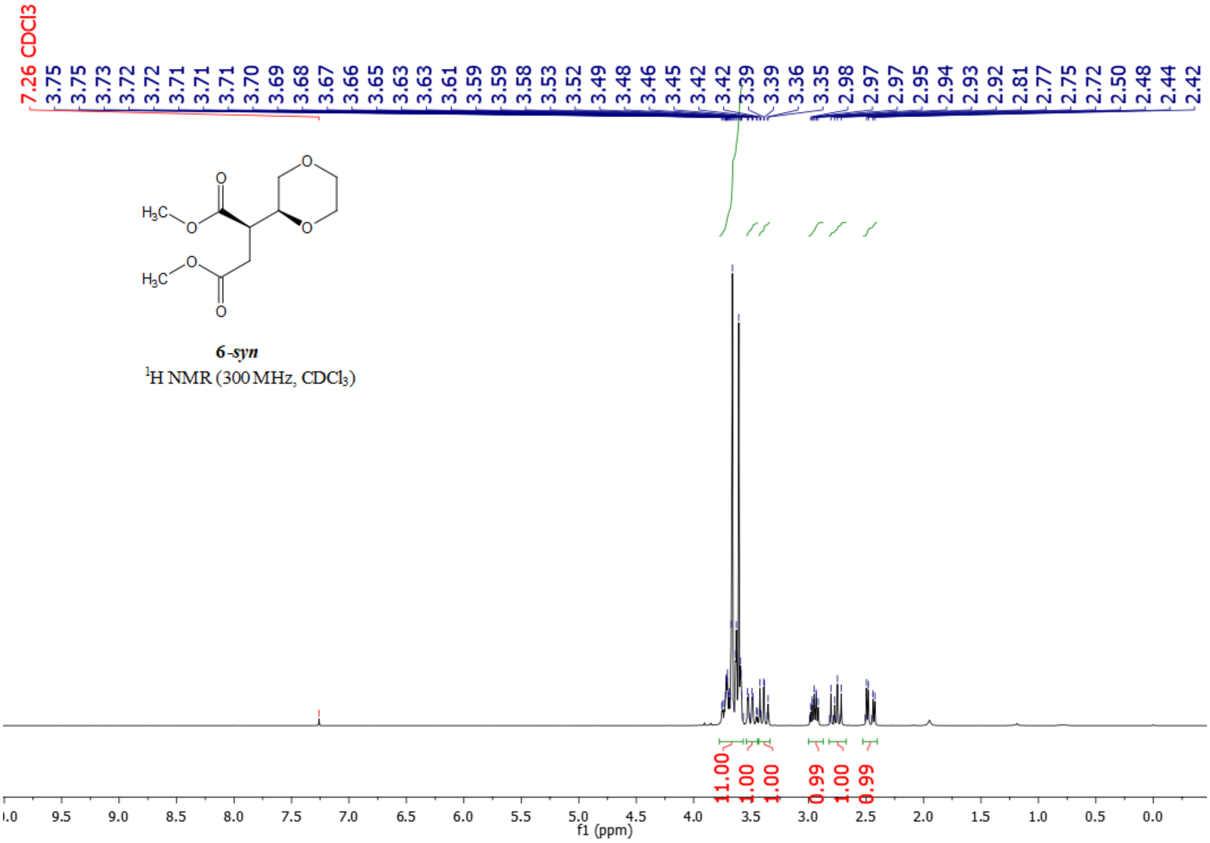

Supplementary Figure 53. ^{1}H NMR (300 MHz, $CDCl_3$) spectrum of compound **6-*syn***.

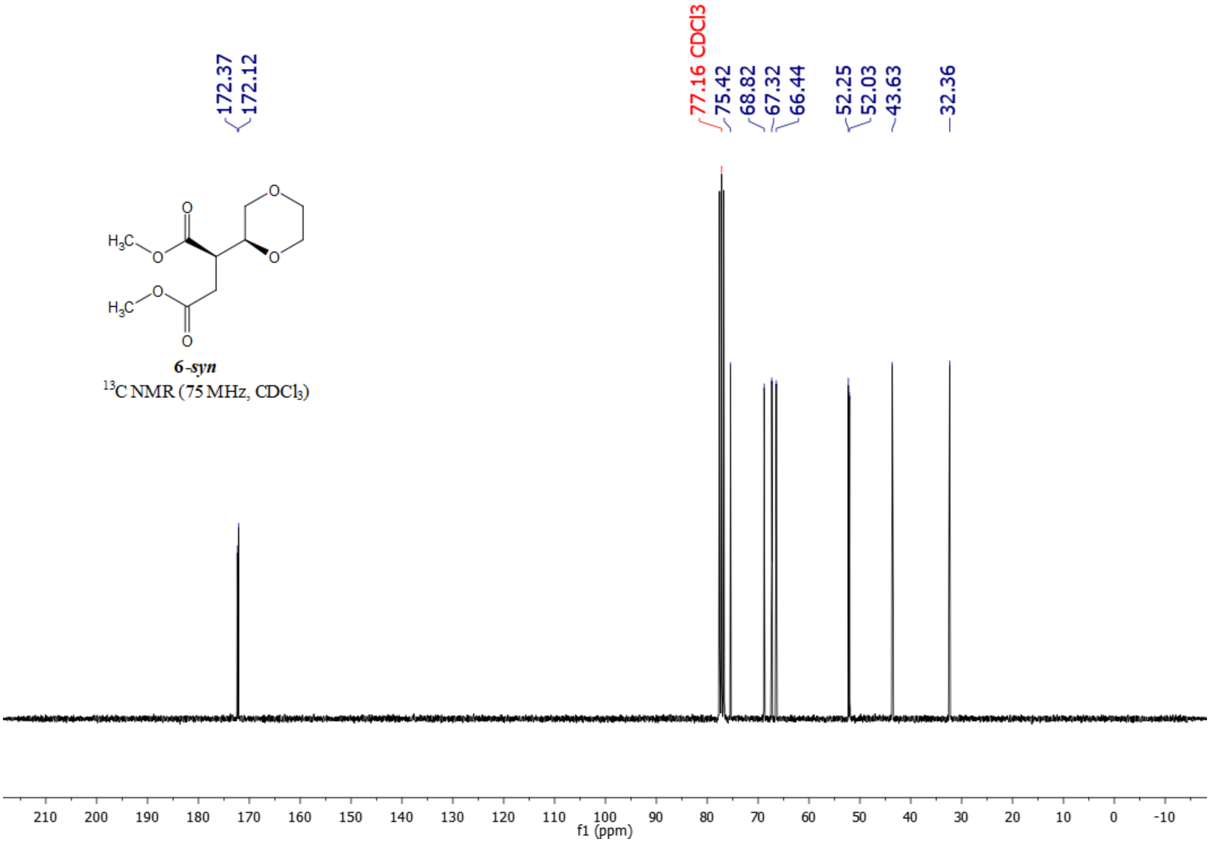

Supplementary Figure 54. ^{13}C NMR (75 MHz, $CDCl_3$) spectrum of compound **6-*syn***.

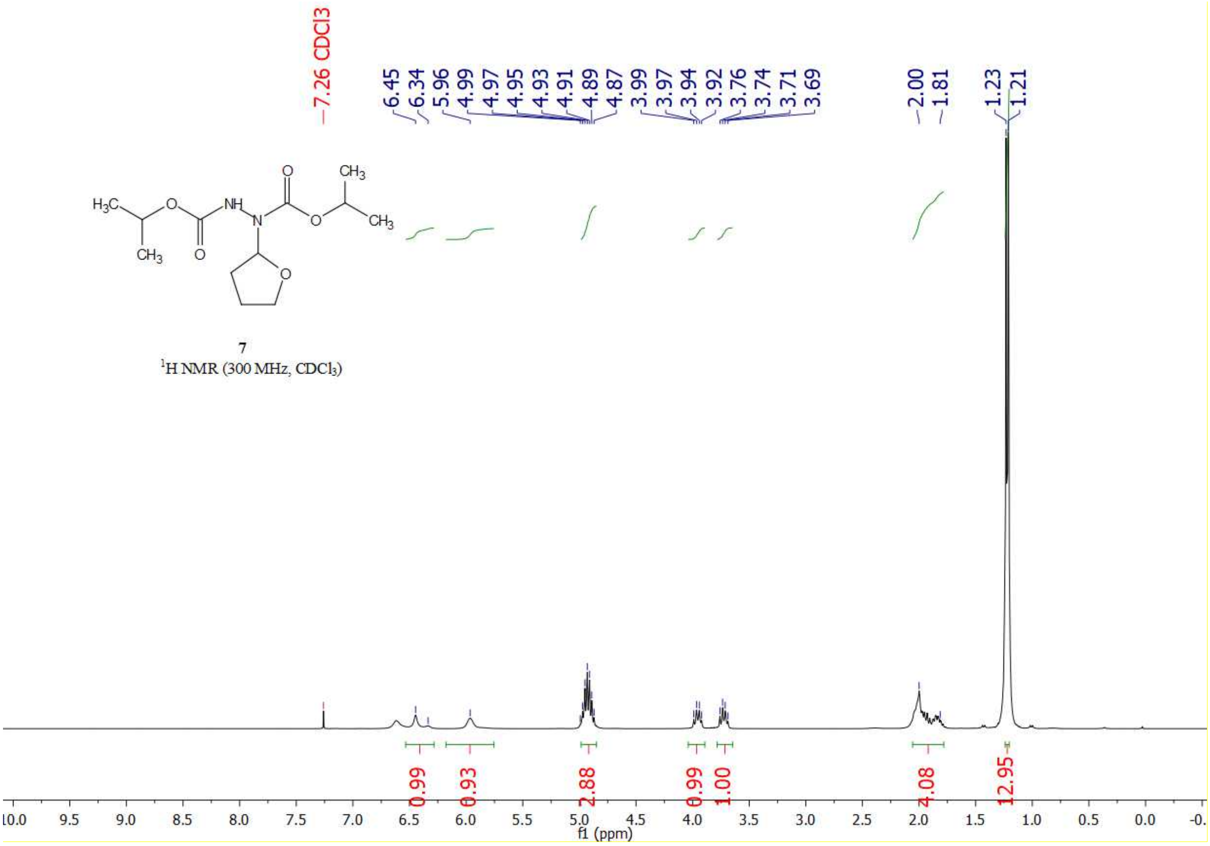

Supplementary Figure 55. ^{1}H NMR (300 MHz, $CDCl_3$) spectrum of compound **7**.

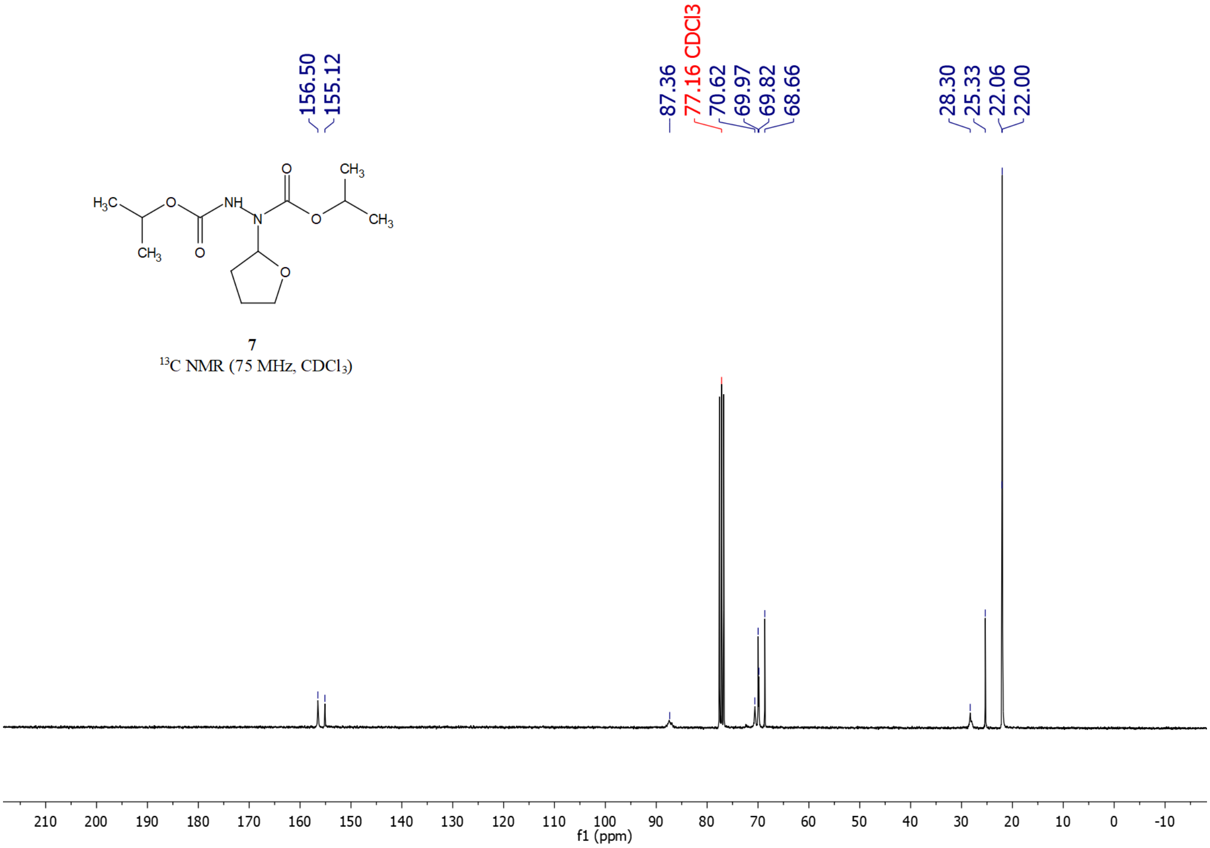

Supplementary Figure 56. ^{13}C NMR (75 MHz, $CDCl_3$) spectrum of compound **7**.

Supplementary reference

1. Protti S, Ravelli D, Fagnoni M, Albini A. Solar light-driven photocatalyzed alkylations. Chemistry on the window ledge. *Chem Commun*, 7351-7353 (2009).
2. Marchetti P, Jimenez Solomon MF, Szekely G, Livingston AG. Molecular Separation with Organic Solvent Nanofiltration: A Critical Review. *Chem Rev* **114**, 10735-10806 (2014).
3. O'Neal EJ, Jensen KF. Continuous Nanofiltration and Recycle of a Metathesis Catalyst in a Microflow System. *ChemCatChem* **6**, 3004-3011 (2014).
4. Fodi T, Didaskalou C, Kupai J, Balogh GT, Huszthy P, Szekely G. Nanofiltration-Enabled In Situ Solvent and Reagent Recycle for Sustainable Continuous-Flow Synthesis. *ChemSusChem* **10**, 3435-3444 (2017).
5. Razali M, Didaskalou C, Kim JF, Babaei M, Drioli E, Lee YM, Szekely G. Exploring and Exploiting the Effect of Solvent Treatment in Membrane Separations. *ACS Appl Mater Interfaces* **9**, 11279-11289 (2017).
6. Low Z-X, Shen J. Determining stability of organic solvent nanofiltration membranes by cross-flow aging. *Sep Purif Technol* **256**, 117840 (2021).
7. Rohe S, Morris AO, McCallum T, Barriault L. Hydrogen Atom Transfer Reactions via Photoredox Catalyzed Chlorine Atom Generation. *Angew Chem Int Ed* **57**, 15664-15669 (2018).
8. Yasu Y, Koike T, Akita M. Visible Light-Induced Selective Generation of Radicals from Organoborates by Photoredox Catalysis. *Adv. Synth.Catal.* **354**, 3414-3420 (2012).
9. Xu Q-H, Wei L-P, Xiao B. Alkyl-GeMe3: Neutral Metalloid Radical Precursors upon Visible-Light Photocatalysis. *Angew Chem Int Ed* **61**, e202115592 (2022).
10. Hayakawa M, Shimizu R, Omori H, Shirota H, Uchida K, Mashimo H, Xu H, Yamada R, Niino S, Wakame Y, Liu C, Aoyama T, Ouchi A. Photochemical addition of cyclic ethers/acetals to olefins using tBuOOtBu: Synthesis of masked ketones/aldehydes and diols. *Tetrahedron* **76**, 131557 (2020).
11. Wan T, Wen Z, Laudadio G, Capaldo L, Lammers R, Rincón JA, García-Losada P, Mateos C, Frederick MO, Broersma R, Noël T. Accelerated and Scalable C(sp^3)–H Amination via Decatungstate Photocatalysis Using a Flow Photoreactor Equipped with High-Intensity LEDs. *ACS Cent Sci* **8**, 51-56 (2022).
